# Return to Sports: A Risky Business? A Systematic Review with Meta-Analysis of Risk Factors for Graft Rupture Following ACL Reconstruction

**DOI:** 10.1007/s40279-022-01747-3

**Published:** 2022-08-24

**Authors:** Anna Cronström, Eva Tengman, Charlotte K. Häger

**Affiliations:** 1 Department of Community Medicine and Rehabilitation, Physiotherapy, Umeå University, Umeå, Sweden; 2 Department of Health Sciences, Lund University, Lund, Sweden

## Abstract

**Background:**

The risk of sustaining a graft rupture after anterior cruciate ligament reconstruction (ACLR) is high. Contributing risk factors are, however, still not clearly identified.

**Objective:**

The aim of this systematic review was to identify and quantify risk factors for graft rupture after ACLR.

**Methods:**

A systematic review with meta-analysis (PROSPERO CRD42020140129) based on PRISMA guidelines was performed. MEDLINE, CINAHL and EMBASE were searched from inception to September 2021. Prospective and retrospective studies addressing risk factors for graft rupture after ACLR in males/females of all ages were considered. Meta-analyses using a random effect model (effect measure: odds ratio [OR] with 95% confidence interval [CI]) were performed. The GRADE tool was used to assess evidence quality.

**Results:**

Following full-text screening of 310 relevant papers, 117 were eventually included, incorporating up to 133,000 individuals in each meta-analysis. Higher Tegner activity level (≥ 7 vs < 7) at primary injury (OR 3.91, 95% CI 1.69–9.04), increased tibial slope (degrees) (OR 2.21, 95% CI 1.26–3.86), lower psychological readiness to return to sport (RTS) (OR 2.18, 95% CI 1.32–3.61), early surgery (< 12 vs ≥ 12 months) (OR 1.87, 95% CI 1.58–2.22), RTS (pre-injury level) (OR 1.87, 95% CI 1.21–2.91) and family history of ACL injury (OR 1.76, 95% CI 1.34–2.31) were all associated with increased odds of graft rupture. Higher age (OR 0.47, 95% CI 0.39–0.59), female sex (OR 0.88, 95% CI 0.79–0.98), fewer self-reported knee symptoms pre-reconstruction (OR 0.81, 95% CI 0.69–0.95) and concomitant cartilage injuries (OR 0.70, 95% CI 0.62–0.79) instead decreased the odds. Meta-analysis revealed no association between body mass index, smoking, joint laxity, RTS time, knee kinematics, muscle strength or hop performance and graft rupture.

**Conclusion:**

Conspicuous risk factors for graft rupture were mainly sports and hereditary related. Few studies investigated function-related modifiable factors or included sports exposure data.

**Supplementary Information:**

The online version contains supplementary material available at 10.1007/s40279-022-01747-3.

## Key Points

**Table:** 

This systematic review with meta-analysis provides evidence that high activity level, young age, lower psychological readiness, and increased tibial slope are risk factors for graft ruptures following anterior cruciate ligament reconstruction.
Females seem to have lower risk of sustaining a graft rupture compared with males.
Having been little explored, future studies should focus on neuromuscular function and psychological aspects as potential risk factors, since these may be modifiable by training or other interventions.

## Background

Injury to the anterior cruciate ligament (ACL) is common among athletes [1, 2] and often leads to functional impairments, failure to return to sport (RTS) and terminated athletic careers [3, 4]. Surgical reconstruction of the ACL (ACLR) is an increasingly common treatment after injury with an increase in incidence in the US from 40.9 per 10,000 patients in 2004 to 47.8 in 2009 [5]. However, the risk of sustaining a graft rupture after ACLR remains high. Wiggins et al., reported in a systematic review and meta-analysis that approximately 10% of the individuals aged < 25 years who returned to their pre-injury activity level after primary ACLR suffered a secondary ACL injury to their ipsi-lateral knee [6]. A subsequent graft rupture may lead to further substantial decline in function and in quality of life, as well as to increased risk of early onset of knee osteoarthritis [7–10]. Still, risk factors associated with graft rupture remain largely unknown.

Prominent risk factors for primary ACL injury are female sex, increased joint laxity as well as aberrant neuromuscular and biomechanical movement patterns, such as deficits in neuromuscular control of the trunk and lower extremity and higher ground reaction forces during landing [11]. Further, as we reported in a recent systematic review, RTS is the risk factor with the strongest association with sustaining a secondary injury to the ACL of the contralateral leg (C-ACL). Notably, athletes who returned to a high activity level (International Knee Documentation Committee [IKDC] questionnaire, level 1–2) or sports including cutting and pivoting were more likely to sustain a C-ACL injury than those who did not return at all or returned to a lower activity level (odds ratio [OR] 3.3) [12]. Other risk factors for future C-ACL injuries included female sex, age 18 years or younger, family history of ACL injury, and early (≤ 3 months) primary ACL reconstruction. However, it is not known if and if so, to what extent, the factors that contribute to graft ruptures overlap with the risk factors for primary and C-ACL injury. Even when passing certain criteria for sensorimotor function following rehabilitation, there may still be an increased risk of C-ACL injury, while the risk for graft rupture is reduced [13]. Also, while females are reported to have a higher risk of primary and C-ACL injury compared with men [11, 12], they seem to have lower risk of graft rupture [14]. It is important to further disentangle specific risk factors for graft rupture after primary ACL injury in order to identify high-risk individuals. Such knowledge will further facilitate the design of training and rehabilitation protocols aiming at risk reduction for secondary injuries following ACL rupture. Previous narrative [15] and systematic reviews [6, 14, 16–18] on risk factors for graft rupture focus either on specific risk factors, such as sex [14, 17], or only include specific subgroups of studies, such as younger participants [16] or registry studies [18]. To our knowledge, there are no previous studies synthesizing all risk factors for graft rupture without population restrictions. Hence, the aim of this systematic review was to identify and quantify risk factors related to demographics/characteristics, injury, timing of surgery, activity, biomechanics, joint geometry/skeletal maturity, function and patient-reported outcomes that are associated with sustaining a future graft rupture.

## Methods

### Literature Search and Study Selection

This systematic review was reported in accordance with the Preferred Reporting Items for Systematic Reviews and Meta-Analyses (PRISMA) guidelines [19, 20] and was pre-registered (PROSPERO: CRD42020140129).

#### Search Strategy

A systematic search was performed from inception to January 2020 (updated in September 2021) in the following databases: MEDLINE (PubMed), CINAHL and EMBASE with search terms incorporating different aspects of secondary ACL injuries and associated risk factors, previously published in full [12] (Online resource 1, see electronic supplementary material [ESM]). Reference lists of all relevant articles were subsequently manually searched for additional studies.

#### Eligibility Criteria

All studies meeting the following criteria were considered for inclusion: (1) prospective or retrospective studies with any follow-up duration; (2) inclusion of males and/or females of any age with primary ACLR (any graft/surgery technique); (3) assessment of any factor related to demographics/characteristics, injury, timing of surgery, activity/sport, biomechanics, joint geometry/skeletal maturity, function and patient-reported outcomes at baseline; and (4) recording of at least three graft ruptures, defined as clinically verified, MRI verified or self-reported graft rupture or as revision surgery during the study period. Exclusion criteria were (1) animal studies and in vitro studies; (2) case studies, conference abstracts, review papers and editorials; (3) external risk factors, such as weather, equipment, playing surface or possible risk factors related to type of graft and/or surgery technique; and (4) published in a language other than English or a Scandinavian language.

### Data Extraction and Synthesis

All abstracts and full texts were independently screened according to the inclusion/exclusion criteria by two of the authors of this review (AC and ET) using the Covidence software (Veritas Health Innovation). Any disagreements were resolved by a consensus discussion between AC and ET, and if required with the third author (CH). The following data were extracted from the studies: authors, publication date, country, number of participants, sex, age, activity level, number of graft ruptures, time to graft rupture, graft type, follow-up period (years), assessed risk factor/s and effect measure/s. If there were not sufficient data to perform meta-analysis reported in a study, study authors were contacted with a request for additional information if the study was published within the last 10 years. A meta-analysis was performed if there were two or more studies that included the same risk factor for sustaining a graft rupture.

Comprehensive Meta-Analysis software, version 2.2.064 (Englewood, USA) was used for meta-analysis. The odds ratio (95% CI) for each risk factor for sustaining a graft rupture was chosen as the effect measure. The odds ratio was primarily calculated from the number of events and sample size in each group or from mean (SD) as appropriate. If not reported, the reported unadjusted univariable odds ratio was used if available. A random effect model was used because of expected heterogeneity between studies, regarding sex, age, graft types, physical activity level and time duration of follow-up. All meta-analyses and corresponding forest plots were weighted under the random effect model, taking both within-study variance and between-study variance (Tau^2^) into account [21]. The *Q* test and corresponding *I*^2^-statistics were used to calculate the between-study effect measure heterogeneity [22]. A 95% confidence interval excluding the null value of 1 was considered a statistically significant result. For studies reporting associated meniscal injuries/surgeries as risk factors for graft rupture, the results for any meniscal injury/surgery (medial or lateral injury) were included in the meta-analysis. If medial and lateral injury/surgery was reported separately, the result for the lateral side was included since the lateral meniscus is most frequently injured in conjunction with acute ACL injury [23]. In studies reporting data from more than one measuring technique for assessing tibial slope (i.e., anterior, posterior, central slope) in the same participants, the number of participants included in the primary analysis was divided by the number of measuring techniques reported, and each measuring technique was then treated as an independent study [24]. All the cut-off values applied for all the variables in this review (e.g., age ≥ 18 vs < 18 years) were based on those reported in the individual studies.

Subgroup analysis for children/adolescents (C&A) (aged ≤ 19 years) and adults (aged > 19 years) were performed if two or more studies investigated the same risk factor for graft rupture.

### Risk of Bias, Publication Bias and Quality of Evidence Assessments

Two of the authors (AC and ET) independently assessed all included studies for risk of bias using the Quality In Prognosis Studies (QUIPS) tool [25, 26] (Online resource 2, see ESM). If consensus was not reached, further discussions with the third author (CH) were conducted to resolve any disagreements. If the meta-analysis included at least 10 studies and the corresponding *I*^2^ was ≤ 50%, funnel plots with trim and fill were used to evaluate any publication bias [27, 28]. The quality of evidence for each risk factor was likewise assessed by both AC and ET using the Grading of Recommendations Assessment, Development and Evaluation (GRADE) for prognostic studies [29, 30] and discussed among all authors. The QUIPS and GRADE assessments were added after the PROSPERO protocol registration.

## Results

**Fig. 1:**
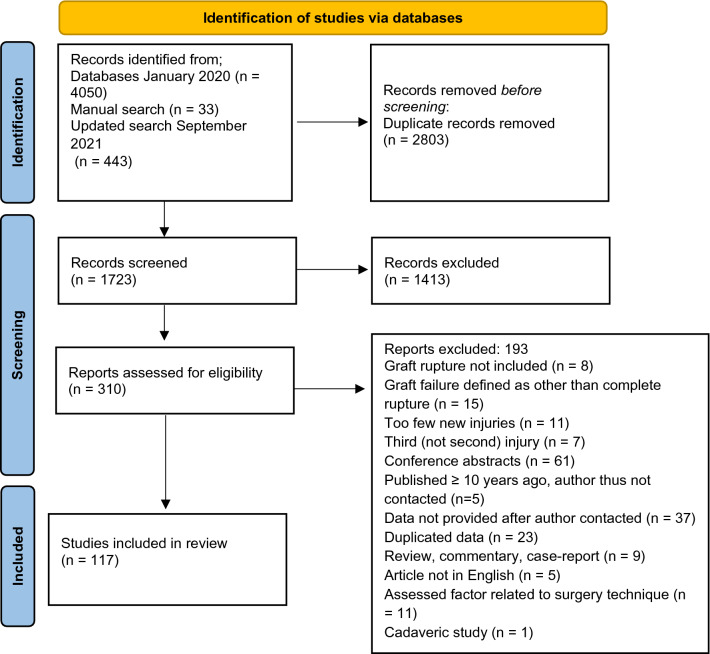
Flow chart of the inclusion process

The systematic search yielded a total of 4493 articles, and another 33 articles were identified by manual search. Of these, 310 full-text papers were then screened according to the inclusion/exclusion criteria and 131 were further excluded. In addition, 52 studies pooled the results for graft rupture with C-ACL injury, or reported the results according to different surgery techniques instead of according to graft rupture/no graft rupture [10, 31–81]. Five of these studies [31–35] were published > 10 years ago and the authors were therefore not contacted. The authors of the remaining 47 studies were contacted by email and data for graft rupture, specifically, were provided for nine studies [36, 39, 41, 43, 46, 48, 49, 77, 79]. Twelve studies [82–93] reported partly on the same participants taken from the Nordic knee ligament registries. Of these, we included one study that included data from all registries (Sweden, Norway, Denmark) [88] and another two studies reporting on specific data not included in the first study (patient-reported outcomes, timing of surgery [82] and RTS [92]). Data from 15 other studies were also partly reported on in previous publications [94–105] [106–108]. Of those, the studies with the largest sample size, the most included risk factors and/or reporting statistics allowing calculation of ORs were included [94, 97, 101, 104, 106]. Altogether, 117 articles were included in this review [9, 36, 39, 41, 43, 46, 48, 49, 72, 73, 77, 79, 82, 88, 92, 94, 97, 101, 104, 106, 109–205] (Fig. 1).

### Study Characteristics

Twelve of the included articles [185–196] were not eligible for meta-analysis (e.g., only one study assessing the specific risk factor or reported statistics not possible to calculate as odds ratios). The characteristics and results of these studies are reported in Online resource 3 (see ESM). Consequently, 105 studies were included in the meta-analysis. Seventy-three studies reported on sex, 45 on age, 27 on activity level and/or sports participation, 21 on associated injuries, 14 on body mass index (BMI), 11 on family history, 12 on tibial slope, five on smoking status, four on timing of surgery, four on contact/non-contact injury mechanism, four on hop performance and two on general laxity, growth plate status, femoral condyle ratio, patient-reported outcomes, number of physiotherapy visits, kinematics, muscle strength and psychological readiness to RTS, respectively, (see Online resource 4, Table 1, in the ESM for characteristics of each individual study included in the meta-analyses). Thirty-one of these studies also included additional risk factors not eligible for meta-analysis (e.g., only one study assessing the specific risk factor). The results for these specific factors are also reported in Online resource 3 (see ESM).

### Synthesis of Results

Meta-analyses consisting of between two and 73 studies (*n* = 108–133,128) were performed separately for 42 potential risk factors for graft rupture. Sixteen risk factors were rated as moderate quality, 12 as low and 14 as very low-quality evidence according to GRADE (Table 1).

**Table 1:** Quality of evidence of the included risk factors according to Grading of Recommendations Assessment, Development and Evaluation (GRADE)

Risk factor	GRADE criteria								
	Phase of investigation	Risk of bias	Inconsistency	Indirectness	Impression	Publication bias	Upgrading factors	GRADE quality of evidence	Summary of findings
Age ≥ 18 vs < 18 years	+++	x	−	x	x	x	+	+++	Age < 18 years associated with higher odds of graft rupture
Age ≥ 20 vs < 20 years	+++	x	−	x	x	x	+	+++	Age < 20 years associated with higher odds of graft rupture
Age ≥ 25 vs < 25 years	+++	x	–	x	x	x	+	++	Age < 25 years associated with higher odds of graft rupture
Age ≥ 30 vs < 30 years	+++	x	−	x	x	x	+	+++	Age < 30 years associated with higher odds of graft rupture
Age continuous	+++	x	–	x	x	x	+	+++	Lower age associated with higher odds of graft rupture
Sex	+++	x	x	x	−	x	x	++	Females had lower odds of graft rupture
Marx score at primary injury	+++	x	–	x	−	x	x	−	No association
Tegner score at primary injury	+++	x	x	x	−	x	x	++	Higher score associated with higher odds of graft rupture
Tegner score at primary injury ≥ 7 vs < 7	+++	x	x	x	−	x	+	+++	Score ≥ 7 associated with higher odds of graft rupture
Lateral tibial slope	+++	x	–	x	x	x	x	+	Increased tibial slope associated with higher odds of graft rupture
Medial tibial slope	+++	x	−	x	−	x	x	+	No association
Femoral condyle ratio	+++	x	x	x	−	x	x	++	No association
Duration between injury and surgery ≥ 12 vs < 12 months	+++	x	x	x	x	x	x	+++	Surgery < 12 months associated with higher odds of graft rupture
Duration between injury and surgery ≥ 6 vs < 6 months	+++	−	x	x	−	x	x	+	No association
Duration between injury and surgery ≥ 3 vs < 3 months	+++	x	−	x	x	x	x	++	No association
Return to pre-injury activity level (RTS)	+++	x	x	x	x	x	x	+++	RTS associated with higher risk of graft rupture
Family history of ACL injury	+++	x	x	x	x	x	x	+++	Family history associated with higher risk of graft rupture
KOOS ADL	+++	x	x	x	x	x	x	+++	No association
KOOS pain	+++	x	x	x	x	x	x	+++	No association
KOOS QoL	+++	x	x	x	x	x	x	+++	No association
KOOS sport/recreation	+++	x	x	x	x	x	x	+++	No association
KOOS symptom	+++	x	x	x	−	x	x	++	Higher score associated with decreased odds of graft rupture
Psychological readiness to RTS	+++	−	x	x	−	−	x	−	Lower readiness associated with higher odds of graft rupture
Concomitant cartilage injury	+++	x	x	x	x	x	x	+++	Cartilage injury associated with higher odds of graft rupture
Concomitant meniscal tear	+++	x	x	x	x	x	x	+++	No association
Concomitant meniscal repair	+++	x	−	x	x	x	x	++	No association
Concomitant meniscectomy	+++	x	x	x	x	x	x	+++	No association
Concomitant MCL injury	+++	x	x	x	x	x	x	+++	No association
BMI ≥ 25 vs < 25 kg/m^2^	+++	−	–	x	x	x	x	−	No association
BMI	+++	x	–	x	x	x	x	+	No association
Smoking status	+++	x	−	x	x	x	x	++	No association
Contact vs non-contact mechanism of primary injury	+++	x	−	x	−	x	x	+	No association
General joint laxity	+++	−	–	x	−	x	x	−	No association
Growth plate status	+++	x	−	x	−	x	x	+	No association
Number of physical therapy visits	+++	x	x	x	−	x	x	++	No association
Timing of RTS ≥ 6 vs < 6 months	+++	−	x	x	−	x	x	−	No association
Type of sport (soccer vs other sports)	+++	x	−	x	x	x	x	++	No association
Hop performance (SLHD)	+++	x	x	x	−	x	x	++	No association
Hop performance (THD)	+++	−	x	x	−	x	x	+	No association
Knee abduction	+++	x	x	x	−	x	x	++	No association
Q-ceps peak torque	+++	−	x	x	−	x	x	+	No association
Hamstring peak torque	+++	−	x	x	−	x	x	+	No association

GRADE criteria: +++ = phase III studies, x = no serious limitations, – = moderate limitations, − = serious limitations, + = upgrade by one. GRADE quality: ++++ = high, +++ = moderate, ++ = low, ± = very low quality of evidence

*ACL* anterior cruciate ligament, *ADL* activities of daily living, *BMI* body mass index, *KOOS* Knee injury and Osteoarthritis Outcome Score, *MCL* medial collateral ligament, *Q-ceps* quadriceps, *QoL* quality of life, *RTS* return to sport, *SLHD* single-leg hop for distance, *THD* triple hop for distance

Seven key factors were identified to increase the odds of future graft rupture after ACLR: (1) high activity level with the odds being almost four times higher for those having a Tegner score of ≥ 7 compared with those scoring < 7 at the primary injury (OR 3.91, 95% CI 1.69–9.04, moderate quality evidence); (2) young age (dichotomous variable), with the odds being 2.6–3.5 times higher for those aged < 18–30 years compared with ≥ 18–30 years, respectively (OR 2.59–3.53, 95% CI 1.51–5.55, low to moderate quality evidence); (3) increased lateral tibial slope (degrees) (OR 2.21, 95% CI 1.26–3.86, very low quality evidence); (4) lower psychological readiness to RTS (OR 2.18, 95% CI 1.32–3.61, very low quality evidence); (5) surgery within 12 months compared with surgery ≥ 12 months post-injury (OR 1.87, 95% CI 1.58–2.22, moderate quality evidence); (6) returning to pre-injury activity level (OR 1.87, 95% CI 1.21–2.91, moderate quality evidence); and finally, (7) family history of ACL injury (OR 1.76, 95% CI 1.34–2.31, moderate quality evidence) (Online resource 5, Figs. 1–7, see ESM). In addition, higher age (continuous variable) (OR 0.47, 95% CI 0.38–0.59, moderate quality evidence), female sex (OR 0.88, 95% CI 0.79–0.98, low quality evidence), better pre-reconstruction score on the Knee injury and Osteoarthritis Outcome Score (KOOS) (symptom subscale) (OR 0.81, 95% CI 0.69–0.95, low quality evidence) and concomitant cartilage injury (OR 0.70, 95% CI 0.62–0.79, moderate quality evidence) decreased the odds of sustaining a graft rupture (Online resource 5, Figs. 2, 8–10, see ESM). The following factors were found not to be associated with future graft rupture: BMI, smoking status, contact versus non-contact injury mechanism, medial tibial slope, general joint laxity, pre-reconstruction KOOS score (subscales: pain, activities of daily living, quality of life, sport/recreation), timing of surgery (≥ 3 vs < 3 months or ≥ 6 vs < 6 months), number of physical therapy visits, timing of RTS (≥ 6 vs < 6 months), playing soccer compared with other sports, Marx activity score at primary injury, hop performance, quadriceps strength, hamstring strength, knee abduction after RTS, concomitant meniscal or medial collateral ligament injuries or femoral condyle ratio (Online resource 5, Fig. 7, 11–26, see ESM).

#### Subgroup Analysis

Sex was the sole variable eligible for meta-analysis for the adults. No difference in the odds of sustaining a graft rupture was observed between males and females if only adults were considered (Online resource 5, Fig. 27, see ESM).

Of the factors eligible for meta-analysis in the subgroup of C&A, a family history of ACL injury (OR 2.03, 95% CI 1.13–3.64) was associated with a higher odds of future graft rupture, whereas female sex decreased the odds (OR 0.71, 95% CI 0.57–0.89) (Online resource 5, Figs. 7 and 27, see ESM). On the other hand, the following factors were not associated with the odds of sustaining a future graft rupture in this population: age, BMI, return to pre-injury activity level, growth plate status and concomitant meniscal injury (Online resource 5, Figs. 7, 22–23, 28–30, see ESM).

### Risk of Bias and Heterogeneity

Sex, family history, RTS and concomitant meniscal tear were the only variables eligible for assessment of publication bias. The funnel plots with trim and fill imputations showed no difference in effect measure, indicating no publication bias for either of the variables as risk factors for graft rupture [27] (Online resource 6, Figs. 1–4, see ESM).

Fifty-eight (50%) studies were rated as low risk of bias, 17 (14%) as moderate and 42 (36%) as high risk of bias (Online resource 6, Table 1, see ESM). After sensitivity analyses were performed, excluding articles with high risk of bias [206], a BMI ≥ 25 kg/m^2^ decreased the odds of sustaining a graft rupture. No other differences in the results were observed (Online resource 7, Table 1, see ESM).

*I*^2^ ranged between < 0.001% and 92% for all meta-analyses, indicating low to high heterogeneity between studies [22] (Online resource 5, Figs. 3–30, see ESM).

## Discussion

This systematic review and meta-analysis identified the following factors as associated with graft rupture with moderate quality evidence: a higher pre-injury activity level, younger age (< 20 years), family history of ACL injury, surgery performed within 12 months and RTS. Increased lateral tibial slope and lower psychological readiness to RTS were also associated with sustaining a future graft rupture but with very low to low quality evidence. Female sex decreased the odds (low quality evidence). On the other hand, factors such as smoking status, joint laxity, timing of RTS, kinematics, knee muscle strength and hop performance were not associated with future graft rupture. Few studies investigated factors related to sensorimotor function and neuromuscular control.

A pre-primary injury Tegner score of ≥ 7 compared with a lower activity level was associated with the highest odds (OR 3.91) of sustaining a graft rupture. In addition, and in line with our previous review on risk factors for C-ACL injury [12], return to pre-injury activity level after ACLR was associated with almost twice the odds of future graft rupture, whereas the time point of RTS (< 6 vs ≥ 6 months), or playing soccer compared with other sports, were not related to graft rupture. A high activity level has previously been linked to an increased risk of ACL injury [37], and individuals who have a higher Tegner score prior to their primary injury are reported to be more likely to return to their pre-injury activity level compared with those initially active on a lower level [207]. Taken together, these results corroborate that participating in and returning to a high activity level that imposes substantial load on the knees leads to a higher risk of graft rupture, irrespective of time point of return and the sports involved. While the pre-injury Marx score was not significantly associated with graft rupture, the Marx score was reported in only two studies and has poorer psychometric properties [208], which may explain differences in the result between these two scales of activity level/participation.

Extending the result from a recent meta-analysis reporting younger age to be a risk factor for C-ACL injury [12], younger age was likewise associated with a higher odds of graft rupture in the current review. Those younger than 20 years had an odds ratio of 3.53 for sustaining a future graft rupture compared with those older than 20 years. The fact that the anatomical structures and neuromuscular system are still under development during adolescence may partly explain why young individuals have a greater risk [209, 210]. Secondly, athletes younger than 20 years are often involved in sport at a higher level [150] and also seem to return to sport to a greater extent [51, 104, 157] without having achieved proper knee function [211] compared with older athletes, which also likely contributes to an increased risk in these young individuals. This reasoning is further supported by the absence of any association between age and graft rupture in the analysis including only those aged 19 and younger, when most athletes may return to a more competitive and knee challenging sports level.

In accordance with research on risk factors for both primary [119, 169, 212] and C-ACL injury [12], the current data revealed that those with a parent and/or sibling who had suffered an ACL injury had higher odds (OR 1.76) of sustaining a graft rupture compared with those with no family history of ACL injury. This was true for both adults and those of younger age. Many factors that predispose individuals to knee injury may be hereditary. Suggested explanations may be related to specific gene polymorphisms [213] and/or inherited anatomical, biomechanical and neuromuscular factors [119, 214]. In line with research that reported increased MRI-verified lateral, but not medial tibial slope to be associated with primary ACL injury [215], the current meta-analysis showed that individuals with a greater lateral tibial slope had higher odds of sustaining a graft rupture, whereas there was no association for medial slope. Greater lateral compared with medial slope is suggested to increase anterior tibial translation as well as internal rotation during functional activity, which consequently may increase ACL strain [216–220]. Furthermore, while we found no relation between general joint laxity and graft rupture risk in the current review, Hewett et al. followed two fraternal female twins from baseline screening to when they both sustained an ACL injury and reported both twins to have increased joint laxity, altered joint biomechanics during movement, such as increased knee abduction and reduced knee flexion, and altered muscle activation pattern [214]. Another suggested explanation for the association between a positive family history and ACL injury may be a familial inclination for sport participation [119]. In-depth approaches are, however, warranted regarding which specific hereditary factors have the strongest links to increased primary and secondary ACL injury risk.

Performing ACLR within 12 months from injury increased the odds of sustaining a graft rupture by 87% compared with delayed surgery (≥ 12 months). In contrast to our previous review where a higher risk of sustaining a C-ACL injury was reported for those who received an ACLR within 3 months [12], no difference in graft rupture rate was observed for other surgery time point cut-offs (≥ 3 vs < 3 months or ≥ 6 vs < 6 months). An early reconstruction has previously been associated with a higher post-operative activity level [221] and it is plausible that the group that delayed reconstruction for 12 months or more represents a group of individuals that have a lower pre-injury activity level and/or may not return to their pre-injury activity level and, thus, are less likely to put their knee at risk. Individuals delaying surgery > 12 months may also represent a group of so called ‘copers’, that is, being able to RTS with excellent dynamic knee stability after ACLR [222]. A recent study has shown that copers have approximately three times the odds of rehabilitation success, including lower graft rupture rate, compared with non-copers [223], which may partly explain our result.

Similar to our previous review on C-ACL injury [12], the meta-analysis showed that concomitant cartilage injury at the time of primary injury decreased the odds of sustaining a future graft rupture, whereas no such association was observed for meniscal injuries. Given that individuals with concomitant cartilage injury are reported to have decreased self-reported knee function, worse knee symptoms, lower quadriceps muscle strength and reduced activity level post-surgery compared with those without cartilage damage [118, 224–226], these individuals may not return to sport and thereby decrease the risk of re-injury to either knee. The relationships between meniscal injury/other concomitant injuries, activity level and post-surgery function are not unscrambled [225, 226] and such complexities may underlie the lack of association between meniscal injuries and graft rupture in the current analysis. Further studies are needed to disentangle the possible association between concomitant injuries, related functional impairment, failure to RTS and second ACL injuries.

In contrast to previous research reporting female sex to be a risk factor for sustaining both a primary ACL injury [1, 227–230] and a C-ACL injury [12], females had lower odds of sustaining a graft rupture in the current review when both adults and C&A were included in the meta-analysis. This result is in accordance with a recent systematic review that reported females to have lower absolute risk of sustaining a graft rupture compared with males [14]. Hormonal sex differences as well as neuromuscular differences in muscle activation pattern and postural control have been suggested to contribute to the higher risk of ACL injuries in females [11, 231]. This indicates that such factors may play a role in primary injury and that a C-ACL injury may in fact be considered as a primary injury to the contra-lateral leg, whereas other factors may be important for graft rupture. The subgroup analyses further showed that when only adults were included in the analysis, there was no sex difference in the odds of sustaining a graft rupture but that the odds for sustaining a graft rupture for females decreased even more when only those age 19 and younger were considered, indicating that the apparent sex difference is mostly driven by young individuals. This is also supported by a recent systematic review that reported males to have a higher risk of graft rupture than females in individuals younger than 20 years of age [16]. It is known that young males return to sport both earlier, more often and to a higher level compared with their female counterparts [51], which may explain why young males had higher odds of graft rupture than young females. However, in the current review there was no effect of RTS on graft rupture in the subgroup for C&A, which may contradict this hypothesis. It should be noted though, that this particular subgroup analysis (i.e., RTS) included very few studies (*n* = 3), and that the OR (1.72) was quite similar to the OR (1.91) for the full analysis. Furthermore, young males seemed to undergo ACLR using a physeal-sparing technique due to skeletal immaturity and open growth plates to a higher extent than females, which has been suggested to influence graft rupture rate in young males [232]. Neither surgical technique [232] nor growth-plate status seemed, however, to be associated with graft rupture when males and females were pooled (Online resource 5, Fig. 30, see ESM) or stratified by sex [177].

In a previous systematic review, a BMI < 25 kg/m^2^ was associated with higher odds of sustaining a future C-ACL injury, whereas smoking status did not seem to be related to C-ACL injury [12]. Individuals with a high BMI and smokers have been reported to have lower activity levels and worse symptoms and self-reported function after ACLR compared with those with a lower BMI and non-smokers [221]. Notwithstanding, our results did not support any relationship between either BMI or smoking status and future graft rupture. However, when excluding one study with high risk of bias from the meta-analysis, a BMI ≥ 25 kg/m^2^ was associated with decreased odds of sustaining a graft rupture, indicating that any relation between BMI and graft rupture is still to be verified.

Knee kinematics, kinetics, knee muscle strength, hop performance and self-reported outcomes, such as knee confidence, have previously been linked to the risk of second ACL injuries (graft ruptures and C-ACL injuries combined) [31, 40, 50, 233]. In the current review, few articles on objective and self-reported function as risk factors for graft rupture as a separate entity were eligible for meta-analysis (too few studies on same factor assessed at same time point or pooling of graft rupture and C-ACL injury). Psychological factors, such as negative emotions, stress, lack of knee confidence and fear of re-injury are commonly reported after ACL injury [234–237] and may have a negative impact on both the rehabilitation process [235] and RTS rate [237]. Lower psychological readiness to RTS, as assessed with the ACL Return to Sport after Injury scale [238], 9–12 months post-reconstruction was associated with higher odds of sustaining a future graft rupture in the current meta-analysis. In addition, Paterno et al. [192] reported those with kinesiophobia to be more prone to rupture of the reconstructed ACL (Online resource 3, see ESM). This result further highlights the importance of incorporating psychological aspects into the rehabilitation process after knee injury.

Similar to the findings for primary ACL injury [239, 240], we found no association between peak knee abduction angle during drop landing and the odds of future graft rupture. Notably, the two studies included in this analysis used different measures to assess knee abduction (2D vs 3D) during slightly different tasks (one-leg vs double-leg drop landing). Although 2D and 3D measures of knee abduction seem closely related [241–243] and the knee abduction angle is proposed to be similar during the execution of single-leg and double-leg landings [244, 245], it is possible that these differences obscured the results of the separate studies. Given this and the few studies included in the analysis, the result for knee abduction angle should be interpreted with caution. Furthermore, the meta-analyses revealed no relation between the performance of the single and triple hop for distance or hamstring and quadriceps peak torque, respectively, and future graft rupture. On the other hand, Kyritsis et al., reported lower hamstring to quadriceps ratio when returning to sport to be associated with a higher risk of graft rupture [130] (Online resource 3, see ESM) and better KOOS score on the symptom subscale decreased the odds of graft rupture in our analysis (no associations for other subscales). The few studies included in these analyses (*n* = 2–4) highlight the lack of studies that include the same measures of sensorimotor function or psychological aspects as potential risk factors for graft rupture. Standardized objective and self-reported measures on function and psychological constructs that are responsive to training/intervention, in contrast to demographic factors that are non-modifiable by nature, should thus be considered in future studies on risk factors for secondary knee injuries. This is a prerequisite to fully understand the role of the neuromuscular and psychological factors in the risk of graft rupture after ACLR.

ACL injuries are most frequent in sports, and re-injury incidence is very high [6]. According to the results from this review, the aspects of being a highly active sport athlete, < 20 years, male, and having low psychological readiness to RTS were among the factors associated with higher odds of graft rupture. Clinicians should be prepared to meet the needs of young highly active sports athletes, incorporating psychological aspects into the rehabilitation after ACLR. Being able to RTS after injury is also closely related to the athletic and personal identity [246–248], as well as to regaining long-term quality of life [7, 249]. In light of this and of the International Olympic Committees’ “Athletes’ Rights and Responsibilities Declaration” (Right #7 of the Preamble) [250], athlete rehabilitation efforts post-ACLR as well as future research should focus on a safe return to sport to reduce the high risk of re-injury in athletes.

In this systematic review, we included all studies that assessed risk factors for graft rupture without any restrictions related to either participant demographics, sport exposure, graft type or year of publication, which also increases the generalizability of our findings. Other strengths of our review are the very high number of individuals in most of the meta-analyses (up to 133,000).

However, there are several limitations. Our review includes studies with different definitions of graft rupture, such as clinically or MRI verified ruptures as well as revision surgery identified from surgical records. It is possible that using only revision surgery as outcome may underestimate the graft rupture rate and consequently influence the result of the meta-analysis, since this approach will not capture those who chose to have non-surgical treatment of their second injury. We also pooled studies on males and females and different age groups, but have performed subgroup analysis to account for possible differences between C&A populations and adults. Since only seven of the 117 included articles reported solely on males (*n* = 4) or females (*n* = 3) and all other articles included both sexes, we do not believe that this had any major influence on our result. An additional limitation is that we pooled studies including different types of grafts and surgery techniques for ACLR. Since most of the included studies comprised a mixture of different graft types/surgery techniques or did not report graft type at all, we chose to not perform subgroup analysis for graft type. Although assessing graft type and surgery technique as possible risk factors was beyond the scope of this study, research indicates an advantage for autograft versus allograft and patella graft versus hamstring graft in the risk of graft failure [251, 252], which may be considered in future research. While most of our meta-analyses were associated with low to moderate heterogeneity, a few analyses—age (continuous, ≥ 25 vs < 25 years), BMI, Marx activity scale, lateral tibial slope and general joint laxity—had high heterogeneity measured with *I*^2^ statistics (≥ 75%) [22]. To account for expected heterogeneity, we performed all meta-analyses under the random effect model that incorporates both within-study and between-study variance in the analysis. It should also be noted that most analyses with high heterogeneity included a low number of studies, which may lead to bias of the *I*^2^ statistics [253]. Thus, the *I*^2^ statistics for these specific studies should be interpreted with caution. The mechanisms contributing to ACL injury and graft rupture are most likely multifactorial and incorporate a combination of both demographic factors, such as family history and age, as well as factors related to neuromuscular control and sensorimotor function [11] and cannot be entirely explained by single factors. Other important factors are RTS status and sports exposure. Since we included all studies assessing risk factors for graft rupture, regardless of sports exposure or RTS status, most studies did not provide such information. It has been suggested that, for example, the relation between young age and a higher risk of second ACL injury is more dependent on the higher RTS rate in young individuals than age [38]. Applying a multifactorial model for assessing risk factors for graft rupture was beyond the scope of the current review but it cannot be ruled out that the result for some factors would have been different if several possible risk factors, including RTS status and sports exposure, had been considered in the same model. We do, however, believe that this review could be a starting point for exploring more complex models incorporating all relevant factors for assessing graft rupture risk in future studies. Finally, we used OR as outcome measure in all analyses and the results should, thus, not be interpreted as equal to the risk of sustaining a future graft rupture [254].

## Conclusion

This systematic review with meta-analysis provides evidence that high activity level, RTS, young age, low psychological readiness to RTS, family history of ACL injury, surgery performed within 12 months, and increased tibial slope are all factors related to increased odds of sustaining a future graft rupture. Females seem, however, to have lower odds of graft rupture compared with males. Studies including modifiable risk factors such as neuromotor control were rare. We recommend that future attention in research should be given to factors such as muscle strength and activation, sensorimotor control and movement quality as well as psychological factors, all of which may be responsive to training/intervention, and thus able to be incorporated into rehabilitation protocols aiming at reducing the risk of further knee injuries after ACL injury and facilitating a safe RTS for ACL-injured individuals.

### Supplementary Information

Below is the link to the electronic supplementary material.

Supplementary file1 (PDF 385 KB)

Supplementary file2 (PDF 382 KB)

Supplementary file3 (PDF 727 KB)

Supplementary file4 (PDF 825 KB)

Supplementary file5 (PDF 391 KB)

Supplementary file6 (PDF 611 KB)

Supplementary file7 (PDF 458 KB)

## Declarations

### Availability of data and material

The datasets used and/or analyzed during the current study are available from the corresponding author on reasonable request.
