# Supplementary material for: Return to Sports: A Risky Business? A Systematic Review with Meta-Analysis of Risk Factors for Graft Rupture Following ACL Reconstruction
Source: Sports Med. 2022 Aug 24;53(1):91–110. doi: 10.1007/s40279-022-01747-3 (PMC9807539; doi:10.1007/s40279-022-01747-3)
Supplement: Supplementary file 1 — Supplementary file1 (PDF 385 KB) [file 40279_2022_1747_MOESM1_ESM.pdf]

Online resource 1. Search strategy

(anterior cruciate ligament[MeSH Terms] OR anterior cruciate ligament reconstruction[MeSH Terms] OR anterior cruciate ligament injury[MeSH Terms] OR "lower extremity"[Title/Abstract] OR "ACL injur*"[Title/Abstract] OR "anterior cruciate ligament injur*"[Title/Abstract] OR construct*[Title/Abstract]) AND ("risk factor*"[Title/Abstract] OR "injury risk"[Title/Abstract] OR "associated with"[Title/Abstract] OR predict*[Title/Abstract] OR relat* AND ("graft injur*"[Title/Abstract] OR "second* injur*"[Title/Abstract] OR reinjur*[Title/Abstract] OR re-injur*[Title/Abstract] OR rupture*[Title/Abstract] OR "graft failure*"[Title/Abstract]) OR "contralateral injur*"[Title/Abstract] OR "contra-lateral injur*"[Title/Abstract]).

In CINAHL and EMBASE the search was performed without MeSH-terms. The search was not restricted to any publication date
