# Supplementary material for: Return to Sports: A Risky Business? A Systematic Review with Meta-Analysis of Risk Factors for Graft Rupture Following ACL Reconstruction
Source: Sports Med. 2022 Aug 24;53(1):91–110. doi: 10.1007/s40279-022-01747-3 (PMC9807539; doi:10.1007/s40279-022-01747-3)
Supplement: Supplementary file 2 — Supplementary file2 (PDF 382 KB) [file 40279_2022_1747_MOESM2_ESM.pdf]

**Online resource 2.**

**Table** The Quality In Prognosis studies (QUIPS) checklist for risk of bias

| **Domains** | **Prompting items for Consideration** | **Ratings** |
|---|---|---|
| **Study Participation** | a. Adequate participation in the study by eligible persons<br>b. Description of the source population or population of interest<br>c. Description of the baseline study sample<br>d. Adequate description of the sampling frame and recruitment<br>e. Adequate description of the period and place of recruitment<br>f. Adequate description of inclusion and exclusion criteria | **High bias:** The relationship between the prognostic factor (PF) and outcome is very likely to be different for participants and eligible nonparticipants<br>**Moderate bias**: The relationship between the PF and outcome may be different for participants and eligible nonparticipants<br>**Low bias**: The relationship between the PF and outcome is unlikely to be different for participants and eligible nonparticipants |
| **Study Attrition** | a. Adequate response rate for study participants<br>b. Description of attempts to collect information on participants who dropped out<br>c. Reasons for loss to follow-up are provided<br>d. Adequate description of participants lost to follow-up<br>e. There are no important differences between participants who completed the study and those who did not | **High bias**: The relationship between the PF and outcome is very likely to be different for completing and non-completing participants<br>**Moderate bias**: The relationship between the PF and outcome may be different for completing and non-completing participants<br>**Low bias**: The relationship between the PF and outcome is unlikely to be different for completing and non-completing participants |
| **Prognostic Factor Measurement** | a. A clear definition or description of the PF is provided<br>b. Method of PF measurement is adequately valid and reliable<br>c. Continuous variables are reported or appropriate cut points are used<br>d. The method and setting of measurement of PF is the same for all study participants<br>e. Adequate proportion of the study sample has complete data for the PF<br>f. Appropriate methods of imputation are used | **High bias**: The measurement of the PF is very likely to be different for different levels of the outcome of interest<br>**Moderate bias**: The measurement of the PF may be different for different levels of the outcome of interest<br>**Low bias**: The measurement of the PF is unlikely to be different for different levels of the outcome of interest |

| | | |
|---|---|---|
| | for missing PF data | |
| **Outcome Measurement** | a. A clear definition of the outcome is provided<br>b. Method of outcome measurement used is adequately valid and reliable<br>c. The method and setting of outcome measurement is the same for all study participants | **High bias**: The measurement of the outcome is very likely to be different related to the baseline level of the PF<br><br>**Moderate bias**: The measurement of the outcome may be different related to the baseline level of the PF<br><br>**Low bias**: The measurement of the outcome is unlikely to be different related to the baseline level of the PF |
| **Study Confounding** | a. All important confounders are measured<br>b. Clear definitions of the important confounders measured are provided<br>c. Measurement of all important confounders is adequately valid and reliable<br>d. The method and setting of confounding measurement are the same for all study participants<br>e. Appropriate methods are used if imputation is used for missing confounder data<br>f. Important potential confounders are accounted for in the study design<br>g. Important potential confounders are accounted for in the analysis | **High bias:** The observed effect of the PF on the outcome is very likely to be distorted by another factor related to PF and outcome<br><br>**Moderate bias:** The observed effect of the PF on outcome may be distorted by another factor related to PF and outcome<br><br>**Low bias:** The observed effect of the PF on outcome is unlikely to be distorted by another factor related to PF and outcome |
| **Statistical Analysis and Reporting** | a. Sufficient presentation of data to assess the adequacy of the analytic strategy<br>b. Strategy for model building is appropriate and is based on a conceptual framework or model<br>c. The selected statistical model is adequate for the design of the study<br>d. There is no selective reporting of results | **High bias**: The reported results are very likely to be spurious or biased related to analysis or reporting<br><br>**Moderate bias**: The reported results may be spurious or biased related to analysis or reporting<br><br>**Low bias**: The reported results are unlikely to be spurious or biased related to analysis or |

| | | |
|---|---|---|
| | | reporting |

**Source:** Hayden JA, van der Windt DA, Cartwright JL, Côté P, Bombardier C. Assessing bias in studies of prognostic factors. Ann Intern Med. 2013;158(4):280-6
