# Supplementary material for: Return to Sports: A Risky Business? A Systematic Review with Meta-Analysis of Risk Factors for Graft Rupture Following ACL Reconstruction
Source: Sports Med. 2022 Aug 24;53(1):91–110. doi: 10.1007/s40279-022-01747-3 (PMC9807539; doi:10.1007/s40279-022-01747-3)
Supplement: Supplementary file 3 — Supplementary file3 (PDF 727 KB) [file 40279_2022_1747_MOESM3_ESM.pdf]

Online resource 3. Characteristics and results of the articles and factors excluded from the meta-analysis

**Article, year & country**	**Study design**	**Participants** (n)	**Graft type**	**Number of graft ruptures** (n, (%)	**Time to graft rupture** (months)	**Age** Mean (sd/range)	**Activity level /sports participation**	**Risk factor(s)**	**Follow-up** (mean/ range, years)	**Reason for exclusion**	**Results**	**RoB**
Articles not eligible for meta-analysis												
Defrancesco et al. 2020 [1] USA	Retrospective	476 females, 520 males	Hamstring autograft,allograft, patella autograft	130 (13)	NR	15.1 (2.2)	NR	Age	2.7	Only study reporting on specific risk factor with specific cut-off (age >16<)	No difference in graft rupture rate between those age<16 compared to >16 years (p=0.855)	High
Della Villa et al. 2021 [2] Sweden	Prospective	118 males	Patella, hamstring, Quadriceps , iliotibial, other	11 (9)	23.5	25 (4.3)	All professional soccer players	Age, contact/non contact mechanism, associated injuries , timing of RTS	4.3	Statistics not possible to re-calculate to OR	Those with an isolated primary ACL injury had higher risk of graft rupture (HR = 3.88, 95% CI; 1.02 – 14.7). No association between age (p = 0.608), contact/non contact mechanism (p = 0.112) or timing of RTS (p = 0.199)	Low
Gagliardi et al. 2019 [3] USA	Retrospective	87 males, 92 females	Suture ligament augumentation (SLA), quadriceps autograft (QA)	15 (8)	NR	SLA 13.9 (3.2), QA 15.7 (1.8)	NR	Age, sex, BMI, timing of surgery	SLA 3.2, QA 2.7	Statistics not possible to re-calculate to OR	Age at surgery (p = 0.160), BMI (p = 0.883), sex (p = 0.280) and time from injury to surgery (p = 0.4598) were not associated with the Hazard of graft rupture	Low
Gans et al. 2018 [4] USA	Retrospective	NR	NR	126 (NR)	NR	NR	National collegiate athletic	Sex	9	Statistics not possible to	Men (rate ratio = 4.3) had a higher rate of	High

							association sport			re-calculate to OR	graft ruptures than women (Rate ratio = 3.0), p=0.04	
Goncalves et.al 2017 [5] France	Retrospective	48 males, 27 females	Patella	4 (5)	NR	Bi-meniscal repair, 30.8, uni-meniscal repair 32.6, control 31.9	NR	Bi-meniscal repair	3.6	Only study reporting on specific risk factor	The re-rupture rate was higher in the bi-meniscal repair group (3/15) compared to the other 2 groups (1/60), p=0.02.	Low
Kinsella et.al 2020 [6] USA	Retrospective	16 females, 34 males	Iliotibial band, hamstring, patella. Tibialis anterior	7 (14)	12.7	13.3 (1.9)	NR	Posterolatera l corner (PLC) injuries combined	NR	Only study reporting on specific risk factor	No association between PLC injury and graft rupture (p=0.19)	High
Lai et.al 2018 [7] Australia	Prospective	158 (females/mal es NR)	Hamstring autograft, patella, allograft, other autografts, LARS	37 (23)	15.2 (13.4)	23.5 (3.6)	AFL players	Age, Family history, timing of RTS	2-18	Statistics not possible to re-calculate to OR	Age<21 (RR = 2.5, 95% CI: 1.3-4.8) was associated with a higher risk of graft rupture. A family history of ACL injury (RR = 1.5, 95% CI: 0.7-3.0) and RTS<12 months (RR = 2.3, 95% CI: 0.6-9.1) were not associated with graft rupture	High
Levins et al. 2016 [8] USA	Prospective	25 males, 30 females	Hamstring autograft, patella autograft	11 (20)	NR	Males: with (18 (2.3)) and without (18.5 (2.5)) graft rupture, Female: with (15.9 (0.8) and without (16.6 (1.2) graft rupture	Participation High school/colleg e level sport	Joint geometry	2-5.5	Statistics not possible to re-calculate to OR	Increased posterior-inferior-directed slope of the articular cartilage in the lateral tibial plateau measured at 2 locations (hazard ratio [HR] = 1.50, p = .029; HR = 1.39,	High

											P = .006), increased volume (HR = 1.45, p = .01) and anteroposterior length (HR = 1.34, = .002) of the medial tibial spine, increased length (HR = 1.18, p = .001) and mediolateral width (HR = 2.19, p = .001) of the lateral tibial spine were associated with graft rupture in males. In females, decreased volume (HR = 0.45, p = .020) and height (HR = 0.46, p = .020) of the medial tibial spine, decreased slope of the lateral tibial subchondral bone (HR = 0.72, p = .010), decreased height of the posterior horn of the medial meniscus (HR = 0.09, p = .001), and decreased intercondylar notch width at the anterior attachment of the ACL (HR =	

											0.72, p = .020) were associated with graft rupture. No associations between 20 other unique geometric measurments and graft ruptures	
Magnussen et al. 2018 [9] USA	Prospective	1023 females, 1302 males	Bone tendon bone autograft, hamstring autograft, quadriceps autograft, allograft	147 (6)	NR	26.7 (11.1)	Recreational/ competetiv sports participation at primary injury	Pre-operative knee laxity	6	Only study defining laxity as >10< degrees	High grade pre-operative knee laxity was associated with higher odds of revision surgery (OR = 1.73, p = 0.04)	Low
Patel et al. 2019 [10] USA	Retrospective	521 females, 535 males	Hamstring autograft, tibialis posterior allograft, hybrid autograft, allograft, patella autograft, iliotibial band autograft, other	76 (7)	NR	15.1 (2.4)	92% participated in sports at primary injury	BMI	2.2	Only study defining BMI as percentiles	No difference in the rate of graft rupture between those with normal BMI (10.8%) and those with overweight (10.0%), p = 0.74	Low
Paterno et al. 2018 [11] USA	Prospective	40 (females/males NR)	NR	8 (20)	NR	16.2 (3.4)	IKDC level 1-2 at primary injury	Fear of movement/re-injury	1 (after RTS)	Only study reporting on specific risk factor	Those who sustained a graft rupture had worse score of the Tampa Scale for Kinesiophobia at return to sport (19.8 (4.0) than those who did not (16.4 (3.6)), p = 0.03	High

Sonnery-Cottet et al. 2017 [12] France	Prospective	138 females, 364 males	Bone patella bone autograft, hamstring autograft,	40 (8)	18.5 (9.5)	22.4 (4.0)	Participating in pivoting sports at primary injury	Age, sex, type of sport, pre-operative knee laxity >7< mm,	3.2	Statistics not possible to re-calculate to OR	Those <25 years had increased risk of graft rupture compared to those >25 years (HR = 3.43, p = 0.12). No association between sex, contact vs non contact sport or pre-operative knee laxity and graft rupture (p > 0.05)	Low
Additional risk factors not eligible for meta-analysis from articles included in the meta-analysis for any other risk factor/s												
Benner et al. 2016 [13] USA	Prospective	553 (females/males NR)	Patella autograft	42 (8)	NR	With graft rupture: 18.6 (5.1), without graft rupture: 23.7 (9.4)	Activity score: 8.4	Pre- and post-operative knee hyper extension	4.1	Only study reporting on specific risk factor with specific cut-offs (>6 or <3 degrees)	No difference in graft rupture rate between groups: >6 degrees (6.9%), <3 degrees (9.8), p=0.246)	Moderate
Cooper et al. 2018 [14] USA	Prospective	484 females, 661 males	Patella autograft, allograft	34 (3)	NR	26	NR	Pre-operative knee hyper extension	2	Only study reporting on specific risk factor with specific cut-offs (>5< degrees)	Those with hyper extension >5 degrees had higher risk of graft rupture (OR=2.2, p=0.030)	High
Cruz et al. 2017 [15] USA	Retrospective	24 girls, 79 boys	Allograft, autograft, hybrid	11 (11)	23	12.1 (6.3-15.7)	NR	Knee flexion at 6 weeks post op	1.8	Only study reporting on specific risk factor	No difference in knee flexion between those who sustained a graft rupture and those who did not (p=0.056)	Low
Csapo et al. 2021 [16] Austria	Retrospective	17 females, 13 males	Hamstring, quadriceps	5 (17)	29.4	21.6 (4.0)	Professional ski racers at primary injury	Simple/complex injury, timing of RTS	At least 2 years	Only study reporting on specific risk factor with specific cut	No association between simple/complex injury (OR = 0.5, 95% CI; 0.1 – 5.1), or timing	High

										off (RTS ≥300 vs. < 300 days	of RTS (OR = 1.2, 95% CI; 0.2 – 8.5)	
Engelman et al. 2014 [17] USA	Retrospective	33 girls, 40 boys,	Hamstring autograft, allograft	15 (21)	NR	15 (11.2-18.7)	NR	Age, history of knee surgery, timing of surgery, associated injuries, timing of physical therapy initiation (>2< weeks) post-operative knee laxity (compared to contra-lateral leg) growth plate status, post-operative range of motion	2.7 - 4.2	Statistics not possible to re-calculate to OR	Increased post-operative knee laxity was associated with higher risk of graft failure (HR=5.28, p=0.022). No association between age (p=0.993), history of knee surgery (p=0.583), timing of surgery (p=0.171), additional meniscal surgeries (p=0.228, timing of physical therapy initiation (p=0.103), growth plate status (p=0.142) or post-operative range of motion (p=0.996).	High
Digiacomo et al. 2018 [18] USA	Prospective	12 females, 16 males	NR	14 (50)	16.4	Graft rupture: 17.9 (4.0) No graft rupture: 18.7 (4.0)	Tegner score at primary injury Graft rupture: 8.6 No graft rupture: 8.0	Femoral notch shape index (NSI)	4	Only study reporting on specific risk factor	No differences in NSI between those who sustained a graft rupture and those who did not (p>0.05)	Moderate
Fältström et al. 2016 [19] Sweden	Retrospective	8 986 females, 11 838 males	Hamstring autograft	702 (3)	NR	Graft rupture: 21.9 (7.3) No graft rupture: 27 (9.9)	Soccer, other contact ball sports, other sport/recreation, other	Pre-operative EQ5D	0.5-8.6	Only study reporting on specific risk factor	Those who sustained a graft rupture had lower pre-operative EQ5D	Low

							(causes of injury)				index (0.66 vs 0.68, p=0.003) and lower EQ5D VAS score (60.7 vs 63.3, p=0.005) than those who did not	
Ghosh et al. 2020 [20] Australia	Prospective	32 girls, 68 boys	Hamstring allograft	12 (12)	11	14 (8-16)	NR	Tibial slope, tanner stage	2	Statistics not possible to re-calculate to OR (tibial slope) or only study reporting on specific cut-off (tanner stage 1-3 vs 4-5)	Those with tanner stage 4-5 had higher risk of sustaining a graft rupture (p=0.05). No difference in tibial slope (>12< degrees) between those who sustained a graft rupture and those who did not (p>0.05).	High
Grassi et al. 2019 [21] Italy	Retrospective	18 females, 68 males	Hamstring autograft	43 (50)	NR	Graft rupture: 21.8 (q1-q3: 18.7-26.7) Controls: 23.3 (q1-q3: 19.6-23.2)	NR	Transepicondylar distance (TE), medial femoral condyle ratio (MFC), lateral/medial tibial plateu subluxation (LTP/MTP), notch width index (NWI), notch ratio (NR), lateral/medial femoral condyle width (LFCw/MFCw, lateral/medial plateu width (LTPw/MTPw)	3	Only study with sufficient statistics including specific variables	Those with graft rupture had greater LTP, MTP and MFCw comoared to those with intact graft (p≤0.001). No differences in the other variables between those with graft rupture and intact graft (p>0.05)	Low
Heath et al. 2019 [22]	Retrospective	82 girls. 166 boys	Hamstring allograft	51 (21)	17.5	14.6 (8-17.9)	> 86% participated	Tanner stage	4.5	Only study reporting	Those with Tanner stage 3-	Low

Australia							in sports at primary injury			on specific factor with specific cut-off (Tanner stage 1-2 vs 3-5)	5 had higher risk of sustaining a graft rupture compared to Tanner stage 1-2 (HR=3.7, p=0.01)	
Henle et al. 2018 [23] Switzerland	Prospective	186 females, 195 males	LARS	30 (8)	NR	33 (12)	55% Tegner score of 5 or 6, time point NR	Post-operative knee laxity (side-to-side difference)	2.5	Only study reporting on specific risk factor including sufficient statistics	Increased knee laxity was associated to graft rupture (OR=1.34, p=0.015)	Low
Kamien et al. 2013 [24] USA	Retrospective	98 (females, males NR)	Hamstring	15 (15)	NR	Range. 12-52	Tegner score (time point NR): Graft rupture: 5.5 No graft rupture: 4.8	Tegner level	2	Unclear time point for assessing activity level	No differences in Tegner activity level between those who sustained a graft rupture (5.53) and those who did not (4.81), p=0.463.	High
Kim et al. 2020 [25] South Korea	Retrospective	19 females, 101 males	NR	31 (26)	NR	Graft rupture: 29.3 (8.3) Controls: 31.1 (7.9)	Pre-operative Tegner score: 3	Post-operative Tegner level, associated injuries, pre- and poste operative knee laxity, knee range of motion	4.1	Only study reporting on specific risk factors at specific time point and with specific cut-offs (lachman/pivot test >grade 2<) with sufficient statistics	Those sustained a graft rupture had a higher post-operative tegner level, more often an associated ALL injury and pre- and post-operative knee laxity ≥ grade 2 compared to those who did not (p<0.05). No difference in pre-operative Tegner level or post-operative knee range of motion (p>0.05).	Low

King et al. 2021 [26] Ireland	Prospective	88 males	Patella, hamstring	31 (35)	19.8	18 - 35	All played multidirectional field sport at primary injury	Marx activity scale (at 9 months post injury), hop performance, biomechanics	2	Only study reporting on specific risk factors at specific time	Those who sustained a graft rupture had greater knee flexion angle (p=0.030), lower center of mass to ankle distance (p=0.027) during double-leg drop jump, greater ground contact time during a planned change of direction movement (p≤0.037) and lower degrees of pelvic tilt during an unplanned change of direction movement than those who did not sustain a graft rupture. No differences for Marx activity score or hop performance during single-leg counter movement jump and single-leg drop jump (p>0.05)	High
Krismer et al. 2017 [27] Switzerland	Retrospective	109 females, 155 males	LARS	25 (9)	7.1	30.8 (12.2)	Tegner score at primary injury: 7	Pre-operative knee laxity, extension and flexion deficits	2	Only study reporting on specific risk factors at specific time point	No differences in pre-operative knee laxity, extension or flexion deficits between those who sustained a graft rupture	Moderate

											and those who did not (p>0.05)	
Kyritsis et al. 2016 [28] Qatar	Prospective	158 males	Patella tendon, hamstring graft	26 (16)	3.5 (after RTS)	Graft rupture: 22 (5) No graft rupture: 21 (4)	Professional athletes	Rehabilitation duration, associated injuries combined, hamstring and quadriceps peak muscle strength and hop performance at time of RTS	2.5 (after RTS)	Only study reporting on specific risk factors	Those who sustained a graft rupture had lower hamstring peak torque at 300 °/s (118 vs 128, p=0.004) on the involved leg, lower hamstring to quadriceps ratio at 60°/s on the involved leg (53 vs 58, p=0.004), lower hamstring average power on the involved leg at 60°/s (79 vs 91, p=0.006), 180 °/s (154 vs 173, p=0.003) and 300 °/s (148 vs 171, p=0.02) No other differences at different speeds for quadriceps or hamstring peak torque, average power or work fatiques were observed for the involved or uninvolved legs. No difference in rehabilitation duration (221 vs 247 days, p=0.070), associated injuries (27% vs 45%, p=0.080) or LSI for the	Low

											cross over hop for distance (99 vs 99) between those who sustained a graft rupture and those who did not.	
Mitchell et al. 2021 [29] USA	Retrospective	83 females, 70 males	Patella, quadriceps, hamstring	39 (25)	NR	14.2 (9.3 – 19)	NR		3.5	Tibial translation, tibial rotation, coronal lateral collateral ligament (LCL) sign on MRI	Greater tibial rotation (p=0.042) and LCL sign (p=0.013) on MRI were associated with graft rupture. No association for tibial translation	Low
Mohtadi et al. 2016 [30] Canada	Prospective	147 females, 183 males	Patella, hamstring	17 (5)	18.3	28.5 (9.8)	Tegner Score ≥ 5 at primary injury	Pre-injury activity level	2	Only study reporting on specific risk factor with specific cut-offs (high/low activity level)	No association between activity level and graft rupture (OR=1.21, 95%C; 0.81-1.81)	Low
Poston et al. 2020 [31] USA	Prospective	32 females, 17 males	Hamstring autograft, allograft patella autograft, allografts	7 (14)	<24	16.5 (3.0)	Pivoting or cutting sport at primary injury	Kinematics	2	Only study reporting on specific risk factors	The sum of knee valgus and trunk lean and the sum of knee valugus, trunk lean and pelvis deviation, respectively, were associated with a higher risk of graft rupture (p≤0.02). No associations between individual assessments of trunk lean and	Low

											pelvis deviation and graft rupture (p≥0,12)	
Pinczewski et al. 2007 [32] Australia	Prospective	85 females 95 males	Patella autograft, hamstring autograft	19 (11)	Patella autografts: 63 Hamstring autografts: 50	25 (13-42)	Pivoting, cutting or side-stepping sports at primary injury	Activity level and knee laxity at 2 years	10	Only study reporting on specific risk factors with specific cut-offs (At 2 years post surgery)	Knee laxity at 2 years was associated with graft rupture (p=0.001). No association between activity level at 2 years and graft rupture (p=0.25)	High
Pullen et al. 2016 [33] USA	Retrospective	2728 females, 13601 males	NR	587 (4)	16.6	28.9 (7.6)	94% active duty military	Medication history	7	Only study reporting on specific risk factor	A history of NSAIDs (p<0.001) and COX-2 inhibitors (p=0.021) were associated with a higher risk of graft rupture	Low
Rosenstiel et al. 2019 [34] France	Retrospective	22 females, 48 males	Hamstring autograft	4 (6)	23.9	23.2 (15-37)	Tegner score: 9.3 (1) at primary injury	Age, contact vs non-contact sport, pre-operative knee laxity, associated injuries	3.9	Statistics not possible to re-calculate to OR	Age (HR=1.47, p=0.739), contact vs non-contact sport (HR=1.67, p=0.758), knee laxity (HR≤1.20, p=0.310) or lateral/medial meniscal lesions (HR≤1.84, p=0.705) were not associated with graft rupture.	Low
Salmon et al. 2006 [35] Australia	Prospective	20 females, 47 males	Patella autograft	9 (13)	77.3	27 (NR)	Pivoting, cutting or side-stepping sports at primary injury	Age	5	Only study with sufficient stats including specific cut-off (>21<)	Age < 21 years was associated with a higher risk of graft rupture (OR=10.0, p=0.020)	Low
Salmon et al. 2018 [36]	Prospective	74 females, 105 males	Hamstring autograft	37 (21)	NR	25.8	Streneous, moderate or	Joint geometry	19.7	Only study including	A tibial slope >12 degrees	Low

Australia							light activity at follow-up			specific cut-off for tibial slope (>12< degrees)	was associated with a higher risk of graft rupture (HR=3.0, p=0.001)	
Sanders et al. 2017 [37] USA	Retrospective	535 females, 820 males	Patella autograft, hamstring autograft, allograft	72 (5)	58.8	26.6 (9.9)	NR	Timing of surgery (>12< months), age (>22< years), associated injuries	10	Statistics not possible to re-calculate to OR	Age <22 years (p=0.04) and timing of surgery >12 months post injury (HR=1.10, p=0.009) were associated with a higher risk of graft rupture. Meniscal tear was not associated with graft rupture (p>0.05)	Low
Shelbourne et al. 1998 [38] USA	Prospective	234 women, 480 men	Patella	19 (3)	NR	24.3	Noyes score: 99.7% > 12 at primary injury	Joint geometry	NR	Only study including specific risk factor with specific cut-0ff (≤15mm vs ≥16mm)	No difference in graft rupture rate between those with an intercondylar femoral notch width ≤15mm (2.3%) and those ≥16mm (3.1%), p=0.628.	Moderate
Schlumberger et al. 2017 [39] Germany	Retrospective	916 females, 1551 males	Hamstring, quadriceps	73 (3)	NR	32.4 (12.2)	NR	Age	5	Statistics not possible to re-calculate to OR	No association between age and graft rupture (p>0.05)	Moderate
Tulloch et al. 2019 [40] Australia	Retrospective	23 females, 32 males	LARS	17 (31)	46.8	Graft rupture: 34.6 No graft rupture: 36.6	Cincinatti score at primary injury: 70	Pre injury activity level	7.8	Only study including specific risk factor (Cincinatti-score)	No difference in pre-injury activity level between those who sustained an graft rupture and those who did not (p>0.05)	Low
Webster et al. 2019 [41] Australia	Prospective	129 females, 200 males	Hamstring	50 (15)	33	17.2 (2)	NR	Post operative extension and	5	Only study including specific risk	Larger flexion deficits (OR=2.3,	High

								flexion deficits, knee laxity		factors at specific time point (12 months post surgery)	p=0.05) were associated with graft rupture. No association between extension deficits (OR=0.4, p>0.05) or knee laxity (OR=0.9, p>0.05) and graft rupture.	
Webster et al. 2021 [42] Australia	Prospective	56 females, 73 males	Patella, quadriceps, hamstring	20 (16)	29	17 (1.9)	91% participated in cutting and pivoting sports	Timing of RTS	3.4	Only study including specific risk factor with adequate statistics	No difference in graft rupture rate between those who returned to sport ≥12 months vs <12 months post reconstruction (p>0.005)	High
Yabroudi et al. 2016 [43] USA	Retrospective	139 females, 112 males	Autograft, allograft	21 (8)	NR	26.1 (9.9)	58% competetive level at primary injury	Pre-injury activity level	3.4	Only study including specific risk factor with specific cut-offs (Marx activity score >12<)	Marx score >12 was associated with graft rupture (OR=3.27, p=0.023).	High

NR = not reported, RTS = return to sport, LARS = Ligament Advanced Reinforcement System, BMI = Body Mass Index, IKDC = International Knee Documentation Committee subjective knee form, OR = odds ratio, RR = risk ratio, HR = hazard ratio, AFL = Australian Football league, EQ5D = EuroQol – 5 – dimension descriptive system, NSAID = Non-Steroidal Anti-inflammatory Drug, RoB = risk of bias

References

1. DeFrancesco CJ, Striano BM, Bram JT, Baldwin KD, Ganley TJ: **An In-Depth Analysis of Graft Rupture and Contralateral Anterior Cruciate Ligament Rupture Rates After Pediatric Anterior Cruciate Ligament Reconstruction**. *Am J Sports Med* 2020, **48**(10):2395-2400.
2. Della Villa F, Hägglund M, Della Villa S, Ekstrand J, Waldén M: **High rate of second ACL injury following ACL reconstruction in male professional footballers: an updated longitudinal analysis from 118 players in the UEFA Elite Club Injury Study**. *Br J Sports Med* 2021.

3. Gagliardi AG, Carry PM, Parikh HB, Traver JL, Howell DR, Albright JC: **ACL Repair With Suture Ligament Augmentation Is Associated With a High Failure Rate Among Adolescent Patients**. *Am J Sports Med* 2019, **47**(3):560-566.
4. Gans I, Retzky JS, Jones LC, Tanaka MJ: **Epidemiology of Recurrent Anterior Cruciate Ligament Injuries in National Collegiate Athletic Association Sports: The Injury Surveillance Program, 2004-2014**. *Orthop J Sports Med* 2018, **6**(6):2325967118777823.
5. Gonçalves H, Steltzlen C, Boisrenoult P, Beaufils P, Pujol N: **High failure rate of anterior cruciate ligament reconstruction with bimeniscal repair: A case-control study**. *Orthop Traumatol Surg Res* 2017, **103**(6):943-946.
6. Kinsella SD, Rider SM, Fury MS, Tepolt FA, Ecklund K, Kocher MS: **Concomitant Posterolateral Corner Injuries in Skeletally Immature Patients With Acute Anterior Cruciate Ligament Injuries**. *J Pediatr Orthop* 2020, **40**(6):271-276.
7. Lai CCH, Feller JA, Webster KE: **Fifteen-Year Audit of Anterior Cruciate Ligament Reconstructions in the Australian Football League From 1999 to 2013: Return to Play and Subsequent ACL Injury**. *Am J Sports Med* 2018, **46**(14):3353-3360.
8. Levins JG, Sturnick DR, Argentieri EC, Gardner-Morse M, Vacek PM, Desarno MJ, Tourville TW, Slauterbeck JR, Beynnon BD: **Geometric Risk Factors Associated With Noncontact Anterior Cruciate Ligament Graft Rupture**. *Am J Sports Med* 2016, **44**(10):2537-2545.
9. Magnussen RA, Reinke EK, Huston LJ, Hewett TE, Spindler KP, Amendola A, Andrish JT, Brophy RH, Dunn WR, Flanigan DC *et al*: **Effect of High-Grade Preoperative Knee Laxity on 6-Year Anterior Cruciate Ligament Reconstruction Outcomes**. *Am J Sports Med* 2018, **46**(12):2865-2872.
10. Patel NM, Talathi NS, Bram JT, DeFrancesco CJ, Ganley TJ: **How Does Obesity Impact Pediatric Anterior Cruciate Ligament Reconstruction?** *Arthroscopy* 2019, **35**(1):130-135.
11. Paterno MV, Flynn K, Thomas S, Schmitt LC: **Self-Reported Fear Predicts Functional Performance and Second ACL Injury After ACL Reconstruction and Return to Sport: A Pilot Study**. *Sports Health* 2018, **10**(3):228-233.
12. Sonnery-Cottet B, Saithna A, Cavalier M, Kajetanek C, Temponi EF, Daggett M, Helito CP, Thaunat M: **Anterolateral Ligament Reconstruction Is Associated With Significantly Reduced ACL Graft Rupture Rates at a Minimum Follow-up of 2 Years: A Prospective Comparative Study of 502 Patients From the SANTI Study Group**. *Am J Sports Med* 2017, **45**(7):1547-1557.
13. Benner RW, Shelbourne KD, Gray T: **The Degree of Knee Extension Does Not Affect Postoperative Stability or Subsequent Graft Tear Rate After Anterior Cruciate Ligament Reconstruction With Patellar Tendon Autograft**. *Am J Sports Med* 2016, **44**(4):844-849.
14. Cooper DE, Dunn WR, Huston LJ, Haas AK, Spindler KP, Allen CR, Anderson AF, DeBerardino TM, Lantz BBA, Mann B *et al*: **Physiologic Preoperative Knee Hyperextension Is a Predictor of Failure in an Anterior Cruciate Ligament Revision Cohort: A Report From the MARS Group**. *Am J Sports Med* 2018, **46**(12):2836-2841.
15. Cruz AI, Jr., Fabricant PD, McGraw M, Rozell JC, Ganley TJ, Wells L: **All-Epiphyseal ACL Reconstruction in Children: Review of Safety and Early Complications**. *J Pediatr Orthop* 2017, **37**(3):204-209.
16. Csapo R, Runer A, Hoser C, Fink C: **Contralateral ACL tears strongly contribute to high rates of secondary ACL injuries in professional ski racers**. *Knee Surg Sports Traumatol Arthrosc* 2021, **29**(6):1805-1812.
17. Engelman GH, Carry PM, Hitt KG, Polousky JD, Vidal AF: **Comparison of allograft versus autograft anterior cruciate ligament reconstruction graft survival in an active adolescent cohort**. *Am J Sports Med* 2014, **42**(10):2311-2318.
18. Digiacomo JE, Palmieri-Smith RM, Redman JA, III, Lepley LK: **Examination of Knee Morphology After Secondary Ipsilateral ACL Injury Compared With Those That Have Not Been Reinjured: A Preliminary Study**. *J Sport Rehabil* 2018, **27**(1):73-82.

19. Fältstrom A, Hagglund M, Magnusson H, Forssblad M, Kvist J: **Predictors for additional anterior cruciate ligament reconstruction: data from the Swedish national ACL register**. *Knee Surg Sports Traumatol Arthrosc* 2016, **24**(3):885-894.
20. Ghosh K, Salmon LJ, Heath E, Pinczewski LA, Roe JP: **Transphyseal anterior cruciate ligament reconstruction using living parental donor hamstring graft: excellent clinical results at 2 years in a cohort of 100 patients**. *Knee Surg Sports Traumatol Arthrosc* 2020, **28**(8):2511-2518.
21. Grassi A, Signorelli C, Urrizola F, Macchiarola L, Raggi F, Mosca M, Samuelsson K, Zaffagnini S: **Patients With Failed Anterior Cruciate Ligament Reconstruction Have an Increased Posterior Lateral Tibial Plateau Slope: A Case-Controlled Study**. *Arthroscopy* 2019, **35**(4):1172-1182.
22. Heath EL, Salmon LJ, Cooper R, Pappas E, Roe JP, Pinczewski LA: **5-Year Survival of Pediatric Anterior Cruciate Ligament Reconstruction With Living Donor Hamstring Tendon Grafts**. *Am J Sports Med* 2019, **47**(1):41-51.
23. Henle P, Bieri KS, Brand M, Aghayev E, Bettfuehr J, Haeberli J, Kess M, Eggli S: **Patient and surgical characteristics that affect revision risk in dynamic intraligamentary stabilization of the anterior cruciate ligament**. *Knee Surg Sports Traumatol Arthrosc* 2018, **26**(4):1182-1189.
24. Kamien PM, Hydrick JM, Replogle WH, Go LT, Barrett GR: **Age, graft size, and Tegner activity level as predictors of failure in anterior cruciate ligament reconstruction with hamstring autograft**. *Am J Sports Med* 2013, **41**(8):1808-1812.
25. Kim SH, Park YB, Kim DH, Pujol N, Lee HJ: **Predictive factors for failure of anterior cruciate ligament reconstruction via the trans-tibial technique**. *Arch Orthop Trauma Surg* 2020, **140**(10):1445-1457.
26. King E, Richter C, Daniels KAJ, Franklyn-Miller A, Falvey E, Myer GD, Jackson M, Moran R, Strike S: **Biomechanical but Not Strength or Performance Measures Differentiate Male Athletes Who Experience ACL Reinjury on Return to Level 1 Sports**. *Am J Sports Med* 2021, **49**(4):918-927.
27. Krismer AM, Gousopoulos L, Kohl S, Ateschrang A, Kohlhof H, Ahmad SS: **Factors influencing the success of anterior cruciate ligament repair with dynamic intraligamentary stabilisation**. *Knee Surg Sports Traumatol Arthrosc* 2017, **25**(12):3923-3928.
28. Kyritsis P, Bahr R, Landreau P, Miladi R, Witvrouw E: **Likelihood of ACL graft rupture: not meeting six clinical discharge criteria before return to sport is associated with a four times greater risk of rupture**. *Br J Sports Med* 2016, **50**(15):946-951.
29. Mitchell BC, Siow MY, Bastrom T, Bomar JD, Pennock AT, Parvaresh K, Edmonds EW: **Predictive Value of the Magnetic Resonance Imaging-Based Coronal Lateral Collateral Ligament Sign on Adolescent Anterior Cruciate Ligament Reconstruction Graft Failure**. *Am J Sports Med* 2021, **49**(4):935-940.
30. Mohtadi N, Chan D, Barber R, Paolucci EO: **Reruptures, Reinjuries, and Revisions at a Minimum 2-Year Follow-up: A Randomized Clinical Trial Comparing 3 Graft Types for ACL Reconstruction**. *Clin J Sport Med* 2016, **26**(2):96-107.
31. Poston GR, Schmitt LC, Ithurburn MP, Hugentobler JA, Thomas S, Paterno MV: **Reduced 2-D Frontal Plane Motion During Single-Limb Landing Is Associated With Risk of Future Anterior Cruciate Ligament Graft Rupture After Anterior Cruciate Ligament Reconstruction and Return to Sport: A Pilot Study**. *J Orthop Sports Phys Ther* 2021, **51**(2):82-87.
32. Pinczewski LA, Lyman J, Salmon LJ, Russell VJ, Roe J, Linklater J: **A 10-year comparison of anterior cruciate ligament reconstructions with hamstring tendon and patellar tendon autograft: a controlled, prospective trial**. *Am J Sports Med* 2007, **35**(4):564-574.

33. Pullen WM, Bryant B, Gaskill T, Sicignano N, Evans AM, DeMaio M: **Predictors of Revision Surgery After Anterior Cruciate Ligament Reconstruction**. *Am J Sports Med* 2016, **44**(12):3140-3145.
34. Rosenstiel N, Praz C, Ouanezar H, Saithna A, Fournier Y, Hager JP, Thaunat M, Sonnery-Cottet B: **Combined Anterior Cruciate and Anterolateral Ligament Reconstruction in the Professional Athlete: Clinical Outcomes From the Scientific Anterior Cruciate Ligament Network International Study Group in a Series of 70 Patients With a Minimum Follow-Up of 2 Years**. *Arthroscopy* 2019, **35**(3):885-892.
35. Salmon LJ, Russell VJ, Refshauge K, Kader D, Connolly C, Linklater J, Pinczewski LA: **Long-term outcome of endoscopic anterior cruciate ligament reconstruction with patellar tendon autograft: minimum 13-year review**. *Am J Sports Med* 2006, **34**(5):721-732.
36. Salmon LJ, Heath E, Akrawi H, Roe JP, Linklater J, Pinczewski LA: **20-Year Outcomes of Anterior Cruciate Ligament Reconstruction With Hamstring Tendon Autograft: The Catastrophic Effect of Age and Posterior Tibial Slope**. *Am J Sports Med* 2018, **46**(3):531-543.
37. Sanders TL, Pareek A, Hewett TE, Levy BA, Dahm DL, Stuart MJ, Krych AJ: **Long-term rate of graft failure after ACL reconstruction: a geographic population cohort analysis**. *Knee Surg Sports Traumatol Arthrosc* 2017, **25**(1):222-228.
38. Shelbourne KD, Davis TJ, Klootwyk TE: **The relationship between intercondylar notch width of the femur and the incidence of anterior cruciate ligament tears. A prospective study**. *Am J Sports Med* 1998, **26**(3):402-408.
39. Schlumberger M, Schuster P, Schulz M, Immendörfer M, Mayer P, Bartholomä J, Richter J: **Traumatic graft rupture after primary and revision anterior cruciate ligament reconstruction: retrospective analysis of incidence and risk factors in 2915 cases**. *Knee Surg Sports Traumatol Arthrosc* 2017, **25**(5):1535-1541.
40. Tulloch SJ, Devitt BM, Porter T, Hartwig T, Klemm H, Hookway S, Norsworthy CJ: **Primary ACL reconstruction using the LARS device is associated with a high failure rate at minimum of 6-year follow-up**. *Knee Surg Sports Traumatol Arthrosc* 2019, **27**(11):3626-3632.
41. Webster KE, Feller JA: **Clinical Tests Can Be Used to Screen for Second Anterior Cruciate Ligament Injury in Younger Patients Who Return to Sport**. *Orthop J Sports Med* 2019, **7**(8):2325967119863003.
42. Webster KE, Feller JA, Klemm HJ: **Second ACL Injury Rates in Younger Athletes Who Were Advised to Delay Return to Sport Until 12 Months After ACL Reconstruction**. *Orthop J Sports Med* 2021, **9**(2):2325967120985636.
43. Yabroudi MA, Björnsson H, Lynch AD, Muller B, Samuelsson K, Tarabichi M, Karlsson J, Fu FH, Harner CD, Irrgang JJ: **Predictors of Revision Surgery After Primary Anterior Cruciate Ligament Reconstruction**. *Orthop J Sports Med* 2016, **4**(9):2325967116666039.
