# Supplementary material for: Return to Sports: A Risky Business? A Systematic Review with Meta-Analysis of Risk Factors for Graft Rupture Following ACL Reconstruction
Source: Sports Med. 2022 Aug 24;53(1):91–110. doi: 10.1007/s40279-022-01747-3 (PMC9807539; doi:10.1007/s40279-022-01747-3)
Supplement: Supplementary file 4 — Supplementary file4 (PDF 825 KB) [file 40279_2022_1747_MOESM4_ESM.pdf]

Online resource 4.

Table 1. Characteristics of the studies included in the meta-analysis

**Article, year & country**	**Study design**	**Participants** (n)	**Graft type**	**Number of graft ruptures** (n (%))	**Time to graft rupture** (mean months)	**Age** Mean (sd/range)	**Activity level /sports participation**	**Risk factor(s)**	**Follow-up** (mean/range, years)	**RoB**
Adults, adolescents and children pooled										
Akhtar et al. 2016 [1] United Kingdom	Retrospective	54 females, 129 males	Hamstring autograft, patella autograft	44 (24)	68	With graft rupture: 28 (16-51), Without graft rupture: 28 (15-57)	NR	Generalized joint laxity	NR	High
Akada et al. 2019 [2] Japan	Retrospective	220 females, 206 males	Hamstring	14 (3)	8.5	28.4	Tegner score: 7.0 at primary injury	Associated injuries	2	High
Allen et al. 2016 [3] USA	Retrospective	180 females	Hamstring autograft, patella autograft, allograft	11 (6)	24.8	19.6 (6.9)	Tegner score: 7.9 at primary injury	Playing soccer, RTS	5.7	Low
Annear et al. 2019 [4] Australia	Prospective	19 females, 23 males	Hamstring autograft	5 (12)	7 to 49	28.9 (10.6)	NR	Age, sex, associated injuries	10	High
Barrett et al. 2011 [5] USA	Retrospective	179 females, 238 males	Patella autograft, allograft Hamstrings autograft	63 (15)	NR	<25: 17.8 (12-25) (13-39), >25: 39 (26-59)	<25: Post operative Tegner score 5.7, >25: 4.3	Age	5.2	High
Beischer et al. 2020 [6] Sweden	Retrospective	70 females, 89 males	Patella, hamstring, quadriceps autograft, allograft	10 (6)	NR	21 (4.4)	Tegner score >6 at primary injury	Age, sex, activity level	1.3 (after RTS)	Moder ate

Benner et al. 2016 [7] USA	Prospective	553 (females/males NR)	Patella autograft	42 (8)	NR	With graft rupture: 18.6 (5.1), without graft rupture: 23.7 (9.4)	Activity score: 8.4	age	4.1	Moderate
Bodkin et al. 2021 [8] USA	Prospective	104 females, 88 males	Patella, hamstring	24 (13)	NR	21.2 (9.2)	Tegner score at primary injury: 8.6	Sex, RTS	2	Low
Bourke et al. 2012[9] Australia	Retrospective	241 females, 432 males	Hamstring autograft, patella autograft	75 (11)	60	29 (13-62)	NR	Age, sex, family history, RTS, associated injuries	16	Moderate
Christensen et al. 2015 [10] USA	Retrospective	28 females, 42 males	Hamstring autograft, patella autograft, allograft	35 (50)	12	21	NR	Tibial slope	6.9	Low
Cooper et al. 2018 [11] USA	Prospective	484 females, 661 males	Patella autograft, allograft	34 (3)	NR	26	NR	Age, sex	2	High
Csapo et al. 2021 [12] Austria	Retrospective	17 females, 13 males	Hamstring, quadriceps	5 (17)	29.4	21.6 (4.0)	Professional ski racers at priamary injury	Sex	At least 2 years	High
Digiacomo et al. 2018 [13] USA	Prospective	12 females, 16 males	Patella, semitendinousus, quadriceps	14 (50)	16.4	Graft rupture: 17.9 (4.0) No graft rupture: 18.7 (4.0)	Tegner score at primary injury Graft rupture: 8.6 No graft rupture: 8.0	Tibial slope	4	Moderate
Everhart et al. 2020 [14] USA	Prospective	205 females, 303 males	Hamstring autograft, allograft, hybrid graft	31 (6)	17.6	24 (8.2)	NR	Associated injuries	3.2	Low

Fleming et al. 2013 [15] USA	Prospective RCT	48 females, 42 males	Patella autograft, hamstring autograft	6 (7)	NR	Low tension: 24 (2.7) High tension: 23 (2.1)	Tegner score at primary injury Low tension:7.6 High tension: 7.6	Age, sex, associated injuries, activity level	3	Moderate
Fältström et al. 2016 [16] Sweden	Retrospective	8 986 females, 11 838 males	Hamstring autograft	702 (3)	NR	Graft rupture: 21.9 (7.3) No graft rupture: 27 (9.9)	Soccer, other contact ball sports, other sport/recreation, other (causes of injury)	Timing of surgery, KOOS score	0.5-8.6	Low
Fältström et al. 2021 [17] Sweden	Prospective	117 females	NR	21 (18)	NR	20 (2)	All soccer players	Hop performance	2	Moderate
Gifstad et al. 2014 [18] Norway	Retrospective	19 031 females, 26 371 males	Patella autograft, hamstring autograft, allograft, other grafts	1198 (3)	NR	Patella autograft: 29 (13-71) Hamstring autograft: 26 (7-90)	93% injured during sport participation	Age, sex, playing soccer, associated injuries	3	Low
Goshima et a. 2014 [19] Japan	Retrospective	160 females, 73 males	NR	12 (5)	NR	21 (14-51)	Tegner score: 7 (Time point NR)	Family history	2	High
Grassi et al. 2020 [20] Italy	Retrospective	58 females, 186 males	Hamstring autograft	8 (3)	44	30.7 (10.6)	Tegner score ≥7: 44% <7: 56%	Age, sex, BMI, smoking status, timing of surgery, activity level	10	Low
Grassi et al. 2019 [21] Italy	Retrospective	18 females, 68 males	Hamstring autograft	43 (50)	NR	Graft rupture: 21.8 (q1-q3: 18.7-26.7)	NR	Tibial slope, femoral condyle ratio	3	Low

						Controls: 23.3 (q1-q3: 19.6-23.2)				
Gupta et al. 2020 [22] India	Retrospective	26 females, 314 males	Patella, hamstring	8 (2)	25.5	26 (5.5)	Tegner score at primary injury<br>Graft rupture: 9.1<br>No graft rupture: NR	Age, sex	10	High
t 2018 [23] Switzerland	Prospective	186 females, 195 males	LARS	30 (8)	NR	33 (12)	Tegner score at primary injury: Graft rupture: 6, controls: 5	Age, sex, BMI, smoking status, activity level	2.5	Low
Henle et al. 2018 [24] Switzerland	Prospective	47 females, 51 males	LARS	11 (11)	NR	31.5 (9.8)	Tegner score at primary injury: 5	Age, Sex, activity level	2	Low
Ifran et al. 2020 [25] Singapore	Retrospective	153 females, 598 males	Hamstring autograft, allograft, patella autograft, allograft	44 (6)	32	23 (14-60)	NR	Age, sex, BMI	2-7	Low
Jaecker et al. 2018 [26] Germany	Retrospective	41 females, 85 males	Patella autograft, hamstring autograft	57 (45)	NR	Graft rupture: 26.6 (18-46)<br>No graft rupture: 31.9 (18-68)	NR	Tibial slope	NR	Low
Kaeding et al. 2017 [27] USA	Prospective	1129 females, 1368 males	Patella autograft, hamstring	112 (4)	NR	26.9 (11.4)	Marx score at primary injury: 11.3	Age, sex, BMI, smoking status,	2	Low

			autograft, allograft					associated injuries, playing soccer, activity level		
Kajetanek et al. 2017 [28] France	Retrospective	160 females, 227 males	Hamstring autograft	20 (5)	25	28.6 (13-58)	Tegner score at primary injury: 5.2	Age, sex, tibial slope, Activity level	2.2	Moderate
Kamien et al. 2013 [29] USA	Retrospective	98 (females, males NR)	Hamstring	15 (15)	NR	Range. 12-52	Tegner score (time point NR): Graft rupture: 5.5 No graft rupture: 4.8	Age, sex	2	High
Kim et al. 2020 [30] South Korea	Retrospective	19 females, 101 males	NR	31 (26)	NR	Graft rupture: 29.3 (8.3) No graft ruptures: 31.1 (7.9)	Pre-operative Tegner score: 3	Age, sex, BMI, tibial slope, Associated injuries	4.1	Low
King et al. 2020 [31] Ireland	Prospective	364 females, 1068 males	Patella autograft, hamstring autograft	39 (3)	21.4	24.4 (7.3)	Pre-operative Marx score: 10.9	Age, sex, associated injuries, contact/non-contact injury, activity level	2.4	Low
King et al. 2021 [32] Ireland	Prospective	88 males	Patella, hamstring	31 (35)	19.8	18 - 35	All played multidirectional field sport at primary injury	Hop performance, knee strength, Psychological readiness to RTS	2	High
Krismer et.al 2017 [33] Switzerland	Retrospective	109 females, 155 males	LARS	25 (9)	7.1	30.8 (12.2)	Tegner score at primary injury: 7	Age, sex, BMI, activity level,	2	Moderate

								associated injuries		
Krosshaug et al. 2016 [34] Norway	Prospective	59 females	NR	4 (7)	NR	Graft rupture: 22.7 (3.8) including n=8 with C-ACL injury) No graft rupture: 23.6 (4.1)	Premier league handball players	Knee abduction	1-7	Low
Kyritsis et al. 2016 [35] Qatar	Prospective	158 males	Patella, hamstring	26 (16)	3.5 (after RTS)	Graft rupture: 22 (5) No graft rupture: 21 (4)	Professional athletes	Age, playing soccer, hop performance, muscle strength	2.5 (after RTS)	Low
Laboute et al. 2010 [36] France	Retrospective	64 females, 234 males	Patella autograft, hamstring autograft	26 (9)	Patella autograft: 21.8 Hamstring autograft: 22	Graft rupture: 24 (6) No graft rupture: 26 (7.7)	All athletes playing at regional level or higher	Age, sex, contact/non contact injury	3.5	High
Larson et al. 2017 [37] USA	Prospective	102 females, 81 males	Patella autograft, hamstring autograft, allograft	13 (7)	NR	26.9 (12-62)	Tegner score at primary injury: 6.5	Sex, general joint laxity	6	High
Lee et al. 2021 [38] South Korea	Retrospective	26 females, 72 males	Hamstring	5 (5)	NR	Adolescents: 17.4 (1.3), adults: 32.3 (4.9)		Age	2.3	Low
Leys et al. 2012 [39] Australia	Prospective	85 females, 95 males	Patella autograft,	22 (12)	NR	24.5 (13-52)	81% participated in moderate-to-	Age, sex	15	High

			hamstring autograft				streneous activity at primary injury			
Lord et al. 2020 [40] Sweden	Retrospective	2684 females 3346 males	Hamstring autograft, patella autograft	146 (2)	NR	28.3 (10.7)	NR	Age, Sex, associated injuries,	2	Low
Magnussen et al. 2012 [41] USA	Retrospective	120 females, 136 males	Hamstring autograft	18 (7)	12	25 (10.5)	NR	Age, sex	1.2	High
Maletis et al. 2015 [42] USA	Retrospective	6277 females, 11159 males	Patella autograft, hamstring autograft, allograft	416 (2)	17	27.2 (IR: 18.7-37-7)	NR	Age, sex, BMI	2.4	Low
Mardani-Kivi et al. 2019 [43] Iran	Retrospective	179 females, 836 males	Hamstring autograft	33 (3)	NR	34 (8.9)	Sport inactivity – regular sport activity (Time point NA)	Age, sex, BMI, Family history	6.5	High
McPherson et al. 2019 [44] Australia	Prospective	118 females, 211 males	Patella, hamstring, quadriceps, synthetic graft	34 (10)	NR	25.3 (8.7)	Sports participation at primary injury	Sex, psychological readiness to RTS	2	Low
Mohtadi et al. 2016 [45] Canada	Prospective	147 females, 183 males	Patella, hamstring	17 (5)	18.3	28.5 (9.8)	Tegner Score ≥ 5 at primary injury	Sex	2	Low
Okoroha et al. 2019 [46] USA	Retrospective	130 males	NR	23 (18)	NR	25.3 (3.2)	NFL players	Age	NR	Low
Paterno et al. 2012 [47] USA	Prospective	42 females, 21 males	NR	4 (6)	NR	16.3 (2.9)	IKDC level 1-2	Sex	1	Moderate
Pfeiffer et al. 2018 [48] USA	Retrospective	38 females, 47 males	NR	49 (6)	NR	29.1 (9.7)	NR	Femoral condyle ratio	4.4	Moderate

Pinczewski et al. 2007 [49] Australia	Prospective	85 females 95 males	Patella autograft, hamstring autograft	19 (11)	Patella autografts: 63 Hamstring autografts: 50	25 (13-42)	Pivoting, cutting or side-stepping sports at primary injury	Age, sex	10	High
Poston et al. 2020 [50] USA	Prospective	32 females, 17 males	Hamstring autograft, allograft, patella autograft, allograft	7 (14)	<24	16.5 (3.0)	Pivoting or cutting sport at primary injury	Age, sex, knee abduction	2	Low
Rahardja et al. 2020 [51] New Zealand	Prospective	3111 females, 4291 males	NR	285 (4)	NR	29.1 (10.9)	NR	Age, sex, timing of surgery, associated injuries	1.9	Low
Riff et al. 2017 [52] USA	Retrospective	929 females, 1296 males	Patella auto graft, allograft, hamstring autograft, allograft	40 (2)	54	29.3 (15.1)	NR	Age, sex	15.7	High
Rosenstiel et al. 2019 [53] France	Retrospective	22 females, 48 males	Hamstring autograft	4 (6)	23.9	23.2 (15-37)	Tegner score: 9.3 at primary injury	Sex	3.9	Low
Runer et al. 2020 [54] Austria	Prospective	345 females, 530 males	Quadriceps autograft, hamstring autograft	38 (4)	NR	30.8 (12.4)	Tegner score: 6 at primary injury	Age, sex, activity level	2	Low
Salmon et al. 2005 [55] Australia	Prospective	289 females, 383 males	Patella autograft, hamstring autograft	39 (6)	20	28 (14-62)	IKDC level 1-4 at primary injury	Sex, family history, contact vs non-contact mechanism RTS,	5	Low

								associated injuries		
Salmon et al. 2006 [56] Australia	Prospective	20 females, 47 males	Patella autograft	9 (13)	77.3	27 (NR)	Pivoting, cutting or side-stepping sports at primary injury	Sex, associated injuries	13	Low
Salmon et al. 2018 [57] Australia	Prospective	74 females, 105 males	Hamstring autograft	37 (21)	NR	25.8	Streneous, moderate or light activity at follow-up	Age, sex, family history	19.7	Low
Sanders et al. 2017 [58] USA	Retrospective	535 females, 820 males	Patella autograft, hamstring autograft, allograft	72 (5)	58.8	26.6 (9.9)	NR	sex	10	Low
Sandon et al. 2020 [59] Sweden	Retrospective	252 females, 432 males	Patella autograft, hamstring autograft, allograft	34 (5)	NR	23.5 (7.6)	Soccer players	RTS	10	High
Sauer et al. 2019 [60] Denmark	Retrospective	34 females, 86 males	Hamstring	60 (50)	NR	22.5 (10-51)	NR	Tibial slope	4	Low
Schlumberger et al. 2017 [61] Germany	Retrospective	916 females, 1551 males	Hamstring, quadriceps	73 (3)	NR	32.4 (12.2)	NR	Sex	5	Moderate
Shelbourne et al. 2009 [62] USA	Prospective	552 females, 863 males	Patella autograft	61 (4)	19.2	21.6 (3.6)	Tegner score: > 7 at primary injury	Age, sex, timing of RTS	5	High
Singhal et al. 2007 [63] USA	Retrospective	31 females, 38 males	Tibialis allograft	16 (23)	22	31.7 (19-69)	NR	Age	4.5	High
Sousa et al. 2017 [64] USA	Prospective	131 females, 92 males	Patella autograft, allograft,	10 (4)	44	22 (12-59)	Tegner score: >6.5 at primary injury	Sex, timing of RTS	4	Low

			hamstring autograft, allograft							
Su et al. 2020 [65] USA	Retrospective	59 females, 64 males	NR	46 (37)	NR	21.2 (6.8)	NR	Tibial slope	NR	High
Tagesson et al. 2016 [66] Sweden	Prospective	8 females, 11 males	Hamstring	3 (16)	24-48	Graft rupture: 21.3 (4) No graft rupture: 21.7 (4.4)	Tegner score: 7 at primary injury24-	Age, sex, KOOS score	5	Moder ate
Thompson et al. 2015 [67] Australia	Prospective	44 females, 46 men	Patella autograft	6 (7)	84	25 (15-42)	Pivoting, cutting or side-stepping sports at primary injury	Age, sex, family history	20	Low
Van Eck et al. 2012 [68] USA	Prospective	103 females, 103 males	Hamstring allograft, tibialis allograft	27 (13)	11 (9)	24 (10)	NR	Age, sex, BMI, associated injuries	2.5	Low
Vincent et al. 2017 [69] China	Retrospective	3 females, 39 males	Patella	9 (21)	NR	Graft rupture: 22.8 (3.6) No graft rupture: 21.4 (4.4)	NR	Number of physical therapy visits	5	Low
Wasserstein et al. 2013 [70] USA	Retrospective	4708 females, 8259 males	Allograft, autograft	336 (3)	35	29.5 (10.5)	NR	Age, sex	5.2	Low
Webb et al. 2013 [71] Australia	Prospective	90 females, 91 males	Hamstring autograft	31 (17)	66	26	NR	Sex, tibial slope	15	High
Welling et al. 2020 [72] Netherlands	Prospective	20 females, 44 males	Patella autograft, allograft,	8 (13)	NR	27.7 (8.8)	Amateur team ball sports	RTS	2.1	High

			hamstring autograft							
Webster et al. 2014 [73] Australia	Retrospective	191 females, 370 males	Autograft	25 (4)	20	28.5 (9.9)	NR	Age, sex, family history, RTS, associated injuries, contact/non-contact injury	4.8	Moderate
Webster et al. 2019 [74] Australia	Prospective	129 females, 200 males	Hamstring	50 (15)	33	17.2 (2)	NR	Hop performance	5	High
Webster et al. 2021 [75] Australia	Retrospective	253 females, 405 males	Hamstring	43 (7)	NR	26 (9)	Marx activity score 8.9 - 9.8 at follow-up	Sex	2	High
Wright et al. 2007 [76] USA	Prospective	110 females, 125 males	Patella autograft, allograft, hamstring autograft, allograft	7 (3)	NR	24 (11-54)	NR	Sex	2	High
Yabroudi et al. 2016 [77] USA	Retrospective	139 females, 112 males	Autograft, allograft	21 (8)	NR	26.1 (9.9)	58% competetive level at primary injury	Age, sex, BMI, RTS, timing of surgery	3.4	High
Adults										
Borton et al. 2018 [78] United Kingdom	Retrospective	76 females, 203 males	Hamstring autograft	11 (4)	15	31 (IQR: 23-42)	NR	Sex	4	High
Filbay et al. 2017 [79] Sweden	Prospective	32 females, 86 males	Patella autograft, hamstring autograft	5 (4)	NR	26 (5)	Moderate-high activity level (non professional) at primary injury	Age, BMI, smoking, activity level, number of physical therapy	5	Low

								visits, associated injuries		
Lee et al. 2018 [80] South Korea	Retrospective	12 females, 116 males	Autograft, allograft	64 (50)	48.5	31 (18-60)	NR	Tibial slope	3.1	High
Lee et al. 2017 [81] South Korea	Prospective	19 females, 61 males	Hamstring autograft, tibialis allograft	4 (5)	12	34.4 (9.8)	NR	Sex	2.5	High
Pullen et al. 2016 [82] USA	Retrospective	2735 females, 13601 males	NR	587 (4)	16.6	28.9 (7.6)	94% active duty military	Age, sex, BMI, smoking, associated injuries	7	Low
Shelbourne et al. 1998 [83] USA	Prospective	234 women, 480 men	Patella	19 (3)	NR	24.3	Noyes score: 99.7% > 12 at primary injury	Sex	NR	Moderate
Tulloch et al. 2019 [84] Australia	Retrospective	23 females, 32 males	LARS	17 (30)	46.8	36 (19-58)	Cincinatti score at primary injury: 70	sex	7.8	Low
Children/Adolescents										
Bayomy et al. 2019 [85] USA	Retrospective	18 girls, 41 boys	Hamstring autograft, allograft	6 (10)	NR	12.5 (6.8-16)	NR	Sex	2.8	Low
Bram et al. 2020 [86] USA	Retrospective	237 girls, 213 boys	NR	56 (12)	NR	14.9 (2.2)	89% played sport at primary injury	Sex, family history	4.3	High
Britt et al. 2020 [87] USA	Retrospective	71 girls	Hamstring, patella autograft	8 (11)	<24	15.4 (1.3)	Soccer players	RTS, associated injuries	3.4	Moderate
Cruz et al. 2017 [88] USA	Retrospective	24 girls, 79 boys	Allograft, autograft, hybrid	11 (11)	23	12.1 (6.3-15.7)	NR	Age, sex, BMI, associated injuries	1.8	Low

Ellis et al. 2012 [89] USA	Retrospective	56 girls, 34 boys	Patella autograft, allograft	9 (10)	9	16 (14-18)	All participated in high school athletics at primary injury	Sex	4-11	Low
Engelman et al. 2014 [90] USA	Retrospective	33 girls, 40 boys,	Hamstring allograft, autograft	15 (21)	NR	15 (11.2-18.7)	NR	Sex	2.7 - 4.2	High
Fones et al. 2020 [91] USA	Retrospective	46 girls, 28 boys	Hamstring allograft, autograft, Patella autograft	8 (11)	NR	15.9 (1.5)	Athletes	Sex, RTS, associated injuries	4	Moderate
Ghosh et al. 2020 [92] Australia	Prospective	32 girls, 68 boys	Hamstring allograft	12 (12)	11	14 (8-16)	NR	Sex, family history	2	High
Grassi et al. 2020 [93] Italy	Prospective	30 girls, 64 boys	Hamstring	8 (9)	NR	15.7 (1.5)	34% Tegner activity level >7 at primary injury	Tibial slope	>2	Low
Graziano et al. 2017 [94] USA	Prospective	12 girls, 30 boys	Hamstring autograft	4 (10)	13.7	12 (10-15)	All participated in sports at primary injury (level 1-3)	Sex	NR	High
Heath et al. 2019 [95] Australia	Retrospective	82 girls. 166 boys	Hamstring allograft	51 (21)	17.5	14.6 (8-17.9)	> 86% participated in sports at primary injury	Age, sex, family history, Growth plate status	4.5	Low
Ho et al. 2018 [96] USA	Retrospective	264 girls, 297 boys	Patella autograft, allograft, hamstring autograft, allograft, hybrid	54 (10)	13.6	15.4 (5-19)	NR	Age, sex, growth plate status	1	Low

Larson et al 2016 [97] USA	Prospective	16 girls, 13 boys	Hamstring autograft, tibialis allograft	5 (17)	NR	13.9 (9-16)	All participated in cutting/pivoting sports at priamary injury	Age, sex	4	Low
Mitchell et al. 2021 [98] USA	Retrospective	83 females, 70 males	Patella, quadriceps, hamstring	39 (25)	NR	14.2 (9.3 – 19)	NR	Tibial slope	3.5	Low
Morgan et al. 2016 [99] Australia	Prospective	104 girls, 138 boys	Patella autograft, hamstring autograft	27 (19)	51	13-18	> 91% participated in sports at primary injury	Age, sex, family history, associated injuries, RTS	16.5	Low
Perkins et al. 2019 [100] USA	Retrospective	197 females, 157 males	Hamstring autograft	50 (14)	16	15.3 (10-19)	NR	Age, sex, BMI, associated injuries	2	Low
Ranade et al. 2018 [101] USA	Retrospective	83 (sex NR)	Hamstring	11 (13)	32	12.3 (6.3-15.9)	NR	Age	4	High
Rauck et al. 2021 [102] USA	Retrospective	38 females, 15 males	Patella	4 (8)	15	16.6 (1.3)	All high school athletes	Sex	3.8	High
Schmale et al. 2014 [103] USA	Retrospective	23 females, 6 males	Hamstring autograft, tibialis allograft	4 (14)	39	14 (1.0)	Tegner score: 8 at primary injury	Sex	4	High
Wall et al. 2017 [104] USA	Retrospective	4 girls, 23 boys	Hamstring	3 (11)	26	11 (8-15)	NR	sex	3.6	Low
Webster et al. 2021 [105] Australia	Prospective	56 females, 73 males	Patella, quadriceps, hamstring	20 (16)	29	17 (1.9)	91% participated in cutting and pivoting sports	Sex	3.4	Low

USA = United States of America, RoB = risk of bias, NR = not reported, RTS = return to sport, KOOS = Knee injury and Osteoarthritis Outcome Score, LARS = Ligament Advanced Reinforcement System, BMI = Body Mass Index, NFL = National Football League, IKDC = International Knee Documentation Committee subjective knee form, RoB = risk of bias

1. Akhtar MA, Bhattacharya R, Keating JF: **Generalised ligamentous laxity and revision ACL surgery: Is there a relation?** *Knee* 2016, **23**(6):1148-1153.
2. Akada T, Yamaura I, Gupta A, Sakai H, Takahashi K, Tsuchiya A: **Partial meniscectomy adversely affects return-to-sport outcome after anatomical double-bundle anterior cruciate ligament reconstruction**. *Knee Surg Sports Traumatol Arthrosc* 2019, **27**(3):912-920.
3. Allen MM, Pareek A, Krych AJ, Hewett TE, Levy BA, Stuart MJ, Dahm DL: **Are Female Soccer Players at an Increased Risk of Second Anterior Cruciate Ligament Injury Compared With Their Athletic Peers?** *Am J Sports Med* 2016, **44**(10):2492-2498.
4. Annear PT, Rohr EJ, Hille DM, Gohil S, Ebert JR: **No clinical difference in 10-year outcomes between standard and minimal graft debridement techniques in patients undergoing anterior cruciate ligament reconstruction using autologous hamstrings: a randomized controlled trial**. *Knee Surg Sports Traumatol Arthrosc* 2019, **27**(2):516-523.
5. Barrett AM, Craft JA, Replogle WH, Hydrick JM, Barrett GR: **Anterior cruciate ligament graft failure: a comparison of graft type based on age and Tegner activity level**. *Am J Sports Med* 2011, **39**(10):2194-2198.
6. Beischer S, Gustavsson L, Senorski EH, Karlsson J, Thomeé C, Samuelsson K, Thomeé R: **Young Athletes Who Return to Sport Before 9 Months After Anterior Cruciate Ligament Reconstruction Have a Rate of New Injury 7 Times That of Those Who Delay Return**. *J Orthop Sports Phys Ther* 2020, **50**(2):83-90.
7. Benner RW, Shelbourne KD, Gray T: **The Degree of Knee Extension Does Not Affect Postoperative Stability or Subsequent Graft Tear Rate After Anterior Cruciate Ligament Reconstruction With Patellar Tendon Autograft**. *Am J Sports Med* 2016, **44**(4):844-849.
8. Bodkin SG, Hertel J, Diduch DR, Saliba SA, Novicoff WM, Brockmeier SF, Miller MD, Gwathmey FW, Werner BC, Hart JM: **Predicting ACL Reinjury from Return to Activity Assessments at 6-months Post-Surgery: A Prospective Cohort Study**. *J Athl Train* 2021.
9. Bourke HE, Salmon LJ, Waller A, Patterson V, Pinczewski LA: **Survival of the anterior cruciate ligament graft and the contralateral ACL at a minimum of 15 years**. *Am J Sports Med* 2012, **40**(9):1985-1992.
10. Christensen JJ, Krych AJ, Engasser WM, Vanhees MK, Collins MS, Dahm DL: **Lateral Tibial Posterior Slope Is Increased in Patients With Early Graft Failure After Anterior Cruciate Ligament Reconstruction**. *Am J Sports Med* 2015, **43**(10):2510-2514.
11. Cooper DE, Dunn WR, Huston LJ, Haas AK, Spindler KP, Allen CR, Anderson AF, DeBerardino TM, Lantz BBA, Mann B *et al*: **Physiologic Preoperative Knee Hyperextension Is a Predictor of Failure in an Anterior Cruciate Ligament Revision Cohort: A Report From the MARS Group**. *Am J Sports Med* 2018, **46**(12):2836-2841.

12. Csapo R, Runer A, Hoser C, Fink C: **Contralateral ACL tears strongly contribute to high rates of secondary ACL injuries in professional ski racers**. *Knee Surg Sports Traumatol Arthrosc* 2021, **29**(6):1805-1812.
13. Digiacomo JE, Palmieri-Smith RM, Redman JA, III, Lepley LK: **Examination of Knee Morphology After Secondary Ipsilateral ACL Injury Compared With Those That Have Not Been Reinjured: A Preliminary Study**. *J Sport Rehabil* 2018, **27**(1):73-82.
14. Everhart JS, DiBartola AC, Swank K, Pettit R, Hughes L, Lewis C, Flanigan DC: **Cartilage damage at the time of anterior cruciate ligament reconstruction is associated with weaker quadriceps function and lower risk of future ACL injury**. *Knee Surg Sports Traumatol Arthrosc* 2020, **28**(2):576-583.
15. Fleming BC, Fadale PD, Hulstyn MJ, Shalvoy RM, Oksendahl HL, Badger GJ, Tung GA: **The effect of initial graft tension after anterior cruciate ligament reconstruction: a randomized clinical trial with 36-month follow-up**. *Am J Sports Med* 2013, **41**(1):25-34.
16. Fältstrom A, Hagglund M, Magnusson H, Forssblad M, Kvist J: **Predictors for additional anterior cruciate ligament reconstruction: data from the Swedish national ACL register**. *Knee Surg Sports Traumatol Arthrosc* 2016, **24**(3):885-894.
17. Fältström A, Hägglund M, Hedevik H, Kvist J: **Poor Validity of Functional Performance Tests to Predict Knee Injury in Female Soccer Players With or Without Anterior Cruciate Ligament Reconstruction**. *Am J Sports Med* 2021, **49**(6):1441-1450.
18. Gifstad T, Foss OA, Engebretsen L, Lind M, Forssblad M, Albrektsen G, Drogset JO: **Lower risk of revision with patellar tendon autografts compared with hamstring autografts: a registry study based on 45,998 primary ACL reconstructions in Scandinavia**. *Am J Sports Med* 2014, **42**(10):2319-2328.
19. Goshima K, Kitaoka K, Nakase J, Tsuchiya H: **Familial predisposition to anterior cruciate ligament injury**. *Asia-Pacific Journal of Sports Medicine, Arthroscopy, Rehabilitation and Technology* 2014, **1**(2):62-66.
20. Grassi A, Macchiarola L, Lucidi GA, Stefanelli F, Neri M, Silvestri A, Della Villa F, Zaffagnini S: **More Than a 2-Fold Risk of Contralateral Anterior Cruciate Ligament Injuries Compared With Ipsilateral Graft Failure 10 Years After Primary Reconstruction**. *Am J Sports Med* 2020:363546519893711.
21. Grassi A, Signorelli C, Urrizola F, Macchiarola L, Raggi F, Mosca M, Samuelsson K, Zaffagnini S: **Patients With Failed Anterior Cruciate Ligament Reconstruction Have an Increased Posterior Lateral Tibial Plateau Slope: A Case-Controlled Study**. *Arthroscopy* 2019, **35**(4):1172-1182.
22. Gupta R, Singhal A, Malhotra A, Soni A, Masih GD, Raghav M: **Predictors for Anterior Cruciate Ligament (ACL) Re-injury after Successful Primary ACL Reconstruction (ACLR)**. *Malays Orthop J* 2020, **14**(3):50-56.
23. Henle P, Bieri KS, Brand M, Aghayev E, Bettfuehr J, Haeberli J, Kess M, Eggli S: **Patient and surgical characteristics that affect revision risk in dynamic intraligamentary stabilization of the anterior cruciate ligament**. *Knee Surg Sports Traumatol Arthrosc* 2018, **26**(4):1182-1189.
24. Henle P, Bieri KS, Haeberli J, Arnout N, Victor J, Herbort M, Koesters C, Eggli S: **Surgeon experience with dynamic intraligamentary stabilization does not influence risk of failure**. *Knee Surg Sports Traumatol Arthrosc* 2018, **26**(10):2978-2985.
25. Ifran NN, Mok YR, Krishna L: **Tear Rates of the Ipsilateral ACL Graft and the Contralateral Native ACL Are Similar following ACL Reconstruction**. *J Knee Surg* 2020.
26. Jaecker V, Drouven S, Naendrup JH, Kanakamedala AC, Pfeiffer T, Shafizadeh S: **Increased medial and lateral tibial posterior slopes are independent risk factors for graft failure following ACL reconstruction**. *Arch Orthop Trauma Surg* 2018, **138**(10):1423-1431.

27. Kaeding CC, Pedroza AD, Reinke EK, Huston LJ, Hewett TE, Flanigan DC, Spindler KP: **Change in Anterior Cruciate Ligament Graft Choice and Outcomes Over Time**. *Arthroscopy* 2017, **33**(11):2007-2014.
28. Kajetanek C, Rousseau R, Makridis K, Sanchez M, Dehlin C, Djian P: **Influence of the tibial slope on recurrent ACL ruptures**. *Journal de Traumatologie du Sport* 2017, **34**(1):37-43.
29. Kamien PM, Hydrick JM, Replogle WH, Go LT, Barrett GR: **Age, graft size, and Tegner activity level as predictors of failure in anterior cruciate ligament reconstruction with hamstring autograft**. *Am J Sports Med* 2013, **41**(8):1808-1812.
30. Kim SH, Park YB, Kim DH, Pujol N, Lee HJ: **Predictive factors for failure of anterior cruciate ligament reconstruction via the trans-tibial technique**. *Arch Orthop Trauma Surg* 2020, **140**(10):1445-1457.
31. King E, Richter C, Jackson M, Franklyn-Miller A, Falvey E, Myer GD, Strike S, Withers D, Moran R: **Factors Influencing Return to Play and Second Anterior Cruciate Ligament Injury Rates in Level 1 Athletes After Primary Anterior Cruciate Ligament Reconstruction: 2-Year Follow-up on 1432 Reconstructions at a Single Center**. *Am J Sports Med* 2020, **48**(4):812-824.
32. King E, Richter C, Daniels KAJ, Franklyn-Miller A, Falvey E, Myer GD, Jackson M, Moran R, Strike S: **Biomechanical but Not Strength or Performance Measures Differentiate Male Athletes Who Experience ACL Reinjury on Return to Level 1 Sports**. *Am J Sports Med* 2021, **49**(4):918-927.
33. Krismer AM, Gousopoulos L, Kohl S, Ateschrang A, Kohlhof H, Ahmad SS: **Factors influencing the success of anterior cruciate ligament repair with dynamic intraligamentary stabilisation**. *Knee Surg Sports Traumatol Arthrosc* 2017, **25**(12):3923-3928.
34. Krosshaug T, Steffen K, Kristianslund E, Nilstad A, Mok KM, Myklebust G, Andersen TE, Holme I, Engebretsen L, Bahr R: **The Vertical Drop Jump Is a Poor Screening Test for ACL Injuries in Female Elite Soccer and Handball Players: A Prospective Cohort Study of 710 Athletes**. *Am J Sports Med* 2016, **44**(4):874-883.
35. Kyritsis P, Bahr R, Landreau P, Miladi R, Witvrouw E: **Likelihood of ACL graft rupture: not meeting six clinical discharge criteria before return to sport is associated with a four times greater risk of rupture**. *Br J Sports Med* 2016, **50**(15):946-951.
36. Laboute E, Savalli L, Puig P, Trouve P, Sabot G, Monnier G, Dubroca B: **Analysis of return to competition and repeat rupture for 298 anterior cruciate ligament reconstructions with patellar or hamstring tendon autograft in sportspeople**. *Ann Phys Rehabil Med* 2010, **53**(10):598-614.
37. Larson CM, Bedi A, Dietrich ME, Swaringen JC, Wulf CA, Rowley DM, Giveans MR: **Generalized Hypermobility, Knee Hyperextension, and Outcomes After Anterior Cruciate Ligament Reconstruction: Prospective, Case-Control Study With Mean 6 Years Follow-up**. *Arthroscopy* 2017, **33**(10):1852-1858.
38. Lee DW, Lee JK, Kwon SH, Moon SG, Cho SI, Chung SH, Kim JG: **Adolescents show a lower healing rate of anterolateral ligament injury and a higher rotational laxity than adults after anterior cruciate ligament reconstruction**. *Knee* 2021, **30**:113-124.
39. Leys T, Salmon L, Waller A, Linklater J, Pinczewski L: **Clinical results and risk factors for reinjury 15 years after anterior cruciate ligament reconstruction: a prospective study of hamstring and patellar tendon grafts**. *Am J Sports Med* 2012, **40**(3):595-605.
40. Lord L, Cristiani R, Edman G, Forssblad M, Stålman A: **One sixth of primary anterior cruciate ligament reconstructions may undergo reoperation due to complications or new injuries within 2 years**. *Knee Surg Sports Traumatol Arthrosc* 2020, **28**(8):2478-2485.
41. Magnussen RA, Lawrence JT, West RL, Toth AP, Taylor DC, Garrett WE: **Graft size and patient age are predictors of early revision after anterior cruciate ligament reconstruction with hamstring autograft**. *Arthroscopy* 2012, **28**(4):526-531.

42. Maletis GB, Inacio MC, Funahashi TT: **Risk factors associated with revision and contralateral anterior cruciate ligament reconstructions in the Kaiser Permanente ACLR registry**. *Am J Sports Med* 2015, **43**(3):641-647.
43. Mardani-Kivi M, Mobarakeh MK, Keyhani S, Ebrahim-Zadeh MH, Azari Z: **Contralateral and siblings' knees are at higher risk of ACL tear for patients with a positive history of ACL tear**. *Knee Surg Sports Traumatol Arthrosc* 2019.
44. McPherson AL, Feller JA, Hewett TE, Webster KE: **Psychological Readiness to Return to Sport Is Associated With Second Anterior Cruciate Ligament Injuries**. *Am J Sports Med* 2019, **47**(4):857-862.
45. Mohtadi N, Chan D, Barber R, Paolucci EO: **Reruptures, Reinjuries, and Revisions at a Minimum 2-Year Follow-up: A Randomized Clinical Trial Comparing 3 Graft Types for ACL Reconstruction**. *Clin J Sport Med* 2016, **26**(2):96-107.
46. Okoroha KR, Fidai MS, Tramer JS, Elmenini J, Makhni EC, Verma NN, Bach BR, Moutzouros V: **Length of Time Between Anterior Cruciate Ligament Reconstruction and Return to Sport Does Not Predict Need for Revision Surgery in National Football League Players**. *Arthroscopy* 2019, **35**(1):158-162.
47. Paterno MV, Rauh MJ, Schmitt LC, Ford KR, Hewett TE: **Incidence of contralateral and ipsilateral anterior cruciate ligament (ACL) injury after primary ACL reconstruction and return to sport**. *Clin J Sport Med* 2012, **22**(2):116-121.
48. Pfeiffer TR, Burnham JM, Hughes JD, Kanakamedala AC, Herbst E, Popchak A, Shafizadeh S, Irrgang JJ, Debski RE, Musahl V: **An Increased Lateral Femoral Condyle Ratio Is a Risk Factor for Anterior Cruciate Ligament Injury**. *J Bone Joint Surg Am* 2018, **100**(10):857-864.
49. Pinczewski LA, Lyman J, Salmon LJ, Russell VJ, Roe J, Linklater J: **A 10-year comparison of anterior cruciate ligament reconstructions with hamstring tendon and patellar tendon autograft: a controlled, prospective trial**. *Am J Sports Med* 2007, **35**(4):564-574.
50. Poston GR, Schmitt LC, Ithurburn MP, Hugentobler JA, Thomas S, Paterno MV: **Reduced 2-D Frontal Plane Motion During Single-Limb Landing Is Associated With Risk of Future Anterior Cruciate Ligament Graft Rupture After Anterior Cruciate Ligament Reconstruction and Return to Sport: A Pilot Study**. *J Orthop Sports Phys Ther* 2021, **51**(2):82-87.
51. Rahardja R, Zhu M, Love H, Clatworthy MG, Monk AP, Young SW: **Rates of revision and surgeon-reported graft rupture following ACL reconstruction: early results from the New Zealand ACL Registry**. *Knee Surg Sports Traumatol Arthrosc* 2020, **28**(7):2194-2202.
52. Riff AJ, Luchetti TJ, Weber AE, Chahal J, Bach BR, Jr.: **Thirty-Year Experience With ACL Reconstruction Using Patellar Tendon: A Critical Evaluation of Revision and Reoperation**. *Orthop J Sports Med* 2017, **5**(8):2325967117724345.
53. Rosenstiel N, Praz C, Ouanezar H, Saithna A, Fournier Y, Hager JP, Thaunat M, Sonnery-Cottet B: **Combined Anterior Cruciate and Anterolateral Ligament Reconstruction in the Professional Athlete: Clinical Outcomes From the Scientific Anterior Cruciate Ligament Network International Study Group in a Series of 70 Patients With a Minimum Follow-Up of 2 Years**. *Arthroscopy* 2019, **35**(3):885-892.
54. Runer A, Csapo R, Hepperger C, Herbort M, Hoser C, Fink C: **Anterior Cruciate Ligament Reconstructions With Quadriceps Tendon Autograft Result in Lower Graft Rupture Rates but Similar Patient-Reported Outcomes as Compared With Hamstring Tendon Autograft: A Comparison of 875 Patients**. *Am J Sports Med* 2020, **48**(9):2195-2204.
55. Salmon L, Russell V, Musgrove T, Pinczewski L, Refshauge K: **Incidence and risk factors for graft rupture and contralateral rupture after anterior cruciate ligament reconstruction**. *Arthroscopy* 2005, **21**(8):948-957.
56. Salmon LJ, Russell VJ, Refshauge K, Kader D, Connolly C, Linklater J, Pinczewski LA: **Long-term outcome of endoscopic anterior cruciate ligament reconstruction with patellar tendon autograft: minimum 13-year review**. *Am J Sports Med* 2006, **34**(5):721-732.

57. Salmon LJ, Heath E, Akrawi H, Roe JP, Linklater J, Pinczewski LA: **20-Year Outcomes of Anterior Cruciate Ligament Reconstruction With Hamstring Tendon Autograft: The Catastrophic Effect of Age and Posterior Tibial Slope**. *Am J Sports Med* 2018, **46**(3):531-543.
58. Sanders TL, Pareek A, Hewett TE, Levy BA, Dahm DL, Stuart MJ, Krych AJ: **Long-term rate of graft failure after ACL reconstruction: a geographic population cohort analysis**. *Knee Surg Sports Traumatol Arthrosc* 2017, **25**(1):222-228.
59. Sandon A, Engström B, Forssblad M: **High Risk of Further Anterior Cruciate Ligament Injury in a 10-Year Follow-up Study of Anterior Cruciate Ligament-Reconstructed Soccer Players in the Swedish National Knee Ligament Registry**. *Arthroscopy* 2020, **36**(1):189-195.
60. Sauer S, English R, Clatworthy M: **The influence of tibial slope on anterior cruciate ligament graft failure risk is dependent on graft positioning**. *J Orthop Surg (Hong Kong)* 2019, **27**(1):2309499019834674.
61. Schlumberger M, Schuster P, Schulz M, Immendörfer M, Mayer P, Bartholomä J, Richter J: **Traumatic graft rupture after primary and revision anterior cruciate ligament reconstruction: retrospective analysis of incidence and risk factors in 2915 cases**. *Knee Surg Sports Traumatol Arthrosc* 2017, **25**(5):1535-1541.
62. Shelbourne KD, Gray T, Haro M: **Incidence of subsequent injury to either knee within 5 years after anterior cruciate ligament reconstruction with patellar tendon autograft**. *Am J Sports Med* 2009, **37**(2):246-251.
63. Singhal MC, Gardiner JR, Johnson DL: **Failure of primary anterior cruciate ligament surgery using anterior tibialis allograft**. *Arthroscopy* 2007, **23**(5):469-475.
64. Sousa PL, Krych AJ, Cates RA, Levy BA, Stuart MJ, Dahm DL: **Return to sport: Does excellent 6-month strength and function following ACL reconstruction predict midterm outcomes?** *Knee Surg Sports Traumatol Arthrosc* 2017, **25**(5):1356-1363.
65. Su AW, Bogunovic L, Smith MV, Gortz S, Brophy RH, Wright RW, Matava MJ: **Medial Tibial Slope Determined by Plain Radiography Is Not Associated with Primary or Recurrent Anterior Cruciate Ligament Tears**. *J Knee Surg* 2020, **33**(1):22-28.
66. Tagesson S, Kvist J: **Greater fear of re-injury and increased tibial translation in patients who later sustain an ACL graft rupture or a contralateral ACL rupture: a pilot study**. *J Sports Sci* 2016, **34**(2):125-132.
67. Thompson S, Salmon L, Waller A, Linklater J, Roe J, Pinczewski L: **Twenty-year outcomes of a longitudinal prospective evaluation of isolated endoscopic anterior cruciate ligament reconstruction with patellar tendon autografts**. *Am J Sports Med* 2015, **43**(9):2164-2174.
68. van Eck CF, Schkrohowsky JG, Working ZM, Irrgang JJ, Fu FH: **Prospective analysis of failure rate and predictors of failure after anatomic anterior cruciate ligament reconstruction with allograft**. *Am J Sports Med* 2012, **40**(4):800-807.
69. Vincent YP-h, Yiu-chung W, Patrick YS-h: **Role of Physiotherapy in Preventing Failure of Primary Anterior Cruciate Ligament Reconstruction**. *Journal of Orthopaedics, Trauma and Rehabilitation* 2017, **22**(1):6-12.
70. Wasserstein D, Khoshbin A, Dwyer T, Chahal J, Gandhi R, Mahomed N, Ogilvie-Harris D: **Risk factors for recurrent anterior cruciate ligament reconstruction: a population study in Ontario, Canada, with 5-year follow-up**. *Am J Sports Med* 2013, **41**(9):2099-2107.
71. Webb JM, Salmon LJ, Leclerc E, Pinczewski LA, Roe JP: **Posterior tibial slope and further anterior cruciate ligament injuries in the anterior cruciate ligament-reconstructed patient**. *Am J Sports Med* 2013, **41**(12):2800-2804.
72. Welling W, Benjaminse A, Lemmink K, Gokeler A: **Passing return to sports tests after ACL reconstruction is associated with greater likelihood for return to sport but fail to identify second injury risk**. *Knee* 2020, **27**(3):949-957.
73. Webster KE, Feller JA, Leigh WB, Richmond AK: **Younger patients are at increased risk for graft rupture and contralateral injury after anterior cruciate ligament reconstruction**. *Am J Sports Med* 2014, **42**(3):641-647.

74. Webster KE, Feller JA: **Clinical Tests Can Be Used to Screen for Second Anterior Cruciate Ligament Injury in Younger Patients Who Return to Sport**. *Orthop J Sports Med* 2019, **7**(8):2325967119863003.
75. Webster KE, Murgier J, Feller JA, Klemm HJ, Devitt BM, Whitehead TS: **Preservation of the Tibial Stump During Anterior Cruciate Ligament Reconstruction Surgery Did Not Increase the Rate of Surgery for Symptomatic Cyclops Lesions**. *Orthop J Sports Med* 2021, **9**(4):2325967121992517.
76. Wright RW, Dunn WR, Amendola A, Andrish JT, Bergfeld J, Kaeding CC, Marx RG, McCarty EC, Parker RD, Wolcott M *et al*: **Risk of tearing the intact anterior cruciate ligament in the contralateral knee and rupturing the anterior cruciate ligament graft during the first 2 years after anterior cruciate ligament reconstruction: a prospective MOON cohort study**. *Am J Sports Med* 2007, **35**(7):1131-1134.
77. Yabroudi MA, Björnsson H, Lynch AD, Muller B, Samuelsson K, Tarabichi M, Karlsson J, Fu FH, Harner CD, Irrgang JJ: **Predictors of Revision Surgery After Primary Anterior Cruciate Ligament Reconstruction**. *Orthop J Sports Med* 2016, **4**(9):2325967116666039.
78. Borton ZM, Yasen SK, Mumith A, Wilson AJ: **Mid-bundle positioning of the femoral socket increases graft rupture in anatomic single bundle anterior cruciate ligament reconstruction**. *Knee* 2018, **25**(6):1122-1128.
79. Filbay SR, Roos EM, Frobell RB, Roemer F, Ranstam J, Lohmander LS: **Delaying ACL reconstruction and treating with exercise therapy alone may alter prognostic factors for 5-year outcome: an exploratory analysis of the KANON trial**. *Br J Sports Med* 2017, **51**(22):1622-1629.
80. Lee CC, Youm YS, Cho SD, Jung SH, Bae MH, Park SJ, Kim HW: **Does Posterior Tibial Slope Affect Graft Rupture Following Anterior Cruciate Ligament Reconstruction?** *Arthroscopy* 2018, **34**(7):2152-2155.
81. Lee BH, Jangir R, Kim HY, Shin JM, Chang M, Kim K, Wang JH: **Comparison of anterior cruciate ligament volume after anatomic double-bundle anterior cruciate ligament reconstruction**. *Knee* 2017, **24**(3):580-587.
82. Pullen WM, Bryant B, Gaskill T, Sicignano N, Evans AM, DeMaio M: **Predictors of Revision Surgery After Anterior Cruciate Ligament Reconstruction**. *Am J Sports Med* 2016, **44**(12):3140-3145.
83. Shelbourne KD, Davis TJ, Klootwyk TE: **The relationship between intercondylar notch width of the femur and the incidence of anterior cruciate ligament tears. A prospective study**. *Am J Sports Med* 1998, **26**(3):402-408.
84. Tulloch SJ, Devitt BM, Porter T, Hartwig T, Klemm H, Hookway S, Norsworthy CJ: **Primary ACL reconstruction using the LARS device is associated with a high failure rate at minimum of 6-year follow-up**. *Knee Surg Sports Traumatol Arthrosc* 2019, **27**(11):3626-3632.
85. Bayomy AF, Bompadre V, Schmale GA: **The Impact of Transphyseal Anterior Cruciate Ligament Reconstruction on Lower Extremity Growth and Alignment**. *Arthroscopy* 2019, **35**(3):940-949.
86. Bram JT, Pascual-Leone N, Patel NM, DeFrancesco CJ, Talathi NS, Ganley TJ: **Do Pediatric Patients With Anterior Cruciate Ligament Tears Have a Higher Rate of Familial Anterior Cruciate Ligament Injury?** *Orthop J Sports Med* 2020, **8**(10):2325967120959665.
87. Britt E, Ouillette R, Edmonds E, Chambers H, Johnson K, Bastrom T, Pennock A: **The Challenges of Treating Female Soccer Players With ACL Injuries: Hamstring Versus Bone-Patellar Tendon-Bone Autograft**. *Orthop J Sports Med* 2020, **8**(11):2325967120964884.
88. Cruz AI, Jr., Fabricant PD, McGraw M, Rozell JC, Ganley TJ, Wells L: **All-Epiphyseal ACL Reconstruction in Children: Review of Safety and Early Complications**. *J Pediatr Orthop* 2017, **37**(3):204-209.

89. Ellis HB, Matheny LM, Briggs KK, Pennock AT, Steadman JR: **Outcomes and revision rate after bone-patellar tendon-bone allograft versus autograft anterior cruciate ligament reconstruction in patients aged 18 years or younger with closed physes**. *Arthroscopy* 2012, **28**(12):1819-1825.
90. Engelman GH, Carry PM, Hitt KG, Polousky JD, Vidal AF: **Comparison of allograft versus autograft anterior cruciate ligament reconstruction graft survival in an active adolescent cohort**. *Am J Sports Med* 2014, **42**(10):2311-2318.
91. Fones L, Kostyun RO, Cohen AD, Pace JL: **Patient-Reported Outcomes, Return-to-Sport Status, and Reinjury Rates After Anterior Cruciate Ligament Reconstruction in Adolescent Athletes: Minimum 2-Year Follow-up**. *Orthop J Sports Med* 2020, **8**(11):2325967120964471.
92. Ghosh K, Salmon LJ, Heath E, Pinczewski LA, Roe JP: **Transphyseal anterior cruciate ligament reconstruction using living parental donor hamstring graft: excellent clinical results at 2 years in a cohort of 100 patients**. *Knee Surg Sports Traumatol Arthrosc* 2020, **28**(8):2511-2518.
93. Grassi A, Pizza N, Zambon Bertoja J, Macchiarola L, Lucidi GA, Dal Fabbro G, Zaffagnini S: **Higher risk of contralateral anterior cruciate ligament (ACL) injury within 2 years after ACL reconstruction in under-18-year-old patients with steep tibial plateau slope**. *Knee Surg Sports Traumatol Arthrosc* 2021, **29**(6):1690-1700.
94. Graziano J, Chiaia T, de Mille P, Nawabi DH, Green DW, Cordasco FA: **Return to Sport for Skeletally Immature Athletes After ACL Reconstruction: Preventing a Second Injury Using a Quality of Movement Assessment and Quantitative Measures to Address Modifiable Risk Factors**. *Orthop J Sports Med* 2017, **5**(4):2325967117700599.
95. Heath EL, Salmon LJ, Cooper R, Pappas E, Roe JP, Pinczewski LA: **5-Year Survival of Pediatric Anterior Cruciate Ligament Reconstruction With Living Donor Hamstring Tendon Grafts**. *Am J Sports Med* 2019, **47**(1):41-51.
96. Ho B, Edmonds EW, Chambers HG, Bastrom TP, Pennock AT: **Risk Factors for Early ACL Reconstruction Failure in Pediatric and Adolescent Patients: A Review of 561 Cases**. *J Pediatr Orthop* 2018, **38**(7):388-392.
97. Larson CM, Heikes CS, Ellingson CI, Wulf CA, Giveans MR, Stone RM, Bedi A: **Allograft and Autograft Transphyseal Anterior Cruciate Ligament Reconstruction in Skeletally Immature Patients: Outcomes and Complications**. *Arthroscopy* 2016, **32**(5):860-867.
98. Mitchell BC, Siow MY, Bastrom T, Bomar JD, Pennock AT, Parvaresh K, Edmonds EW: **Predictive Value of the Magnetic Resonance Imaging-Based Coronal Lateral Collateral Ligament Sign on Adolescent Anterior Cruciate Ligament Reconstruction Graft Failure**. *Am J Sports Med* 2021, **49**(4):935-940.
99. Morgan MD, Salmon LJ, Waller A, Roe JP, Pinczewski LA: **Fifteen-Year Survival of Endoscopic Anterior Cruciate Ligament Reconstruction in Patients Aged 18 Years and Younger**. *Am J Sports Med* 2016, **44**(2):384-392.
100. Perkins CA, Busch MT, Christino M, Herzog MM, Willimon SC: **Allograft Augmentation of Hamstring Anterior Cruciate Ligament Autografts Is Associated With Increased Graft Failure in Children and Adolescents**. *Am J Sports Med* 2019, **47**(7):1576-1582.
101. Ranade SC, Refakis CA, Cruz AI, Jr., Leddy KL, Wells L, Lawrence JT, Ganley TJ: **Validated Pediatric Functional Outcomes of All-epiphyseal ACL Reconstructions: Does Reinjury Affect Outcomes?** *J Pediatr Orthop* 2020, **40**(4):157-161.
102. Rauck RC, Apostolakos JM, Nwachukwu BU, Schneider BL, Williams RJ, 3rd, Dines JS, Altchek DW, Pearle A, Allen A, Stein BS *et al*: **Return to Sport After Bone-Patellar Tendon-Bone Autograft ACL Reconstruction in High School-Aged Athletes**. *Orthop J Sports Med* 2021, **9**(6):23259671211011510.

103. Schmale GA, Kweon C, Larson RV, Bompadre V: **High satisfaction yet decreased activity 4 years after transphyseal ACL reconstruction**. *Clin Orthop Relat Res* 2014, **472**(7):2168-2174.
104. Wall EJ, Ghattas PJ, Eismann EA, Myer GD, Carr P: **Outcomes and Complications After All-Epiphyseal Anterior Cruciate Ligament Reconstruction in Skeletally Immature Patients**. *Orthop J Sports Med* 2017, **5**(3):2325967117693604.
105. Webster KE, Feller JA, Klemm HJ: **Second ACL Injury Rates in Younger Athletes Who Were Advised to Delay Return to Sport Until 12 Months After ACL Reconstruction**. *Orthop J Sports Med* 2021, **9**(2):2325967120985636.
