# Supplementary material for: Return to Sports: A Risky Business? A Systematic Review with Meta-Analysis of Risk Factors for Graft Rupture Following ACL Reconstruction
Source: Sports Med. 2022 Aug 24;53(1):91–110. doi: 10.1007/s40279-022-01747-3 (PMC9807539; doi:10.1007/s40279-022-01747-3)
Supplement: Supplementary file 5 — Supplementary file5 (PDF 391 KB) [file 40279_2022_1747_MOESM5_ESM.pdf]

Return to sports - a risky business? A systematic review with meta-analysis of risk factors for graft rupture following ACL reconstruction, Sports Medicine, Anna Cronström; anna.cronstrom@umu.se, Eva Tengman, Charlotte Häger, Umeå University

Online resource 5. Meta-analyses and forest plots of risk factors for graft rupture

Pre-injury activity level

Author & year	Population	Graft	Scale	Follow-up	Odds ratio	Lower limit	Upper limit
Kaeding et al. 2017 [1]	A/C&A	Mix	Marx	2.0	2.88	2.04	4.06
King et al. 2020 [2]	A/C&A	Mix	Marx	2.4	0.90	0.50	1.62
					1.65	0.53	5.16
Beischer et al. 2020 [3]	A/C&A	Mix	Tegner	5.0	2.68	0.83	8.62
Filbay et al. 2017 [4]	A	Mix	Tegner	5.0	2.48	0.40	15.23
Fleming et al. 2013 [5]	A/C&A	Mix	Tegner	3.0	2.66	0.59	12.04
Henle et al. 2018 [6]	A/C&A	LARS	Tegner	2.5	5.13	2.58	10.19
Henle et al. 2018 [7]	A/C&A	LARS	Tegner	2.0	1.55	1.01	2.39
Kajetanek et al. 2017 [8]	A/C&A	Hamstring	Tegner	2.2	1.83	0.81	4.14
Krismer et al. 2017 [9]	A/C&A	LARS	Tegner	2.0	2.70	1.27	5.73
Tagesson et al. 20 .5 [10]	A/C&A	Hamstring	Tegner	5.0	0.26	0.03	2.58
					2.29	1.48	3.55
Grassi et al. 2020 [11]	A/C&A	Hamstring	Tegner >7<	10.0	1.34	0.31	5.72
Krismer et al. 2017 [9]	A/C&A	LARS	Tegner >7<	2.0	4.40	1.21	16.02
Runer et al. 2020 [12]	A/C&A	Mix	Tegner >7<	2.0	6.14	2.87	13.15
					3.91	1.69	9.04

Odds ratio and 95% CI

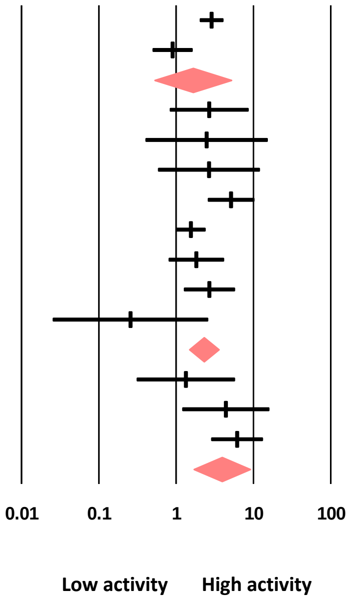

I^2 statistics
Marx scale: 91%, Tegner continuous: 44%
Tegner >7<: 40%

Figure 1. Meta-analysis on the association between pre-injury activity level and graft rupture (Marx scale; graft ruptures n = 150, controls n = 3310), (Tegner scale continuous; graft ruptures n = 109, controls n = 1379), (Tegner scale >7<; graft ruptures n = 71, controls n = 1162). A = adults, C&A = children/adolescents, Mix = mix of different grafts, LARS = Ligament Advanced Reinforcement System, Follow-up = years.

Return to sports - a risky business? A systematic review with meta-analysis of risk factors for graft rupture following ACL reconstruction, Sports Medicine, Anna Cronström; anna.cronstrom@umu.se, Eva Tengman, Charlotte Häger, Umeå University

Age

Author & year	Graft	Age	Follow-up	Odds ratio	Lower limit	Upper limit
Grassi et al. 2020 [11]	Hamstring	>18<	10.0	1.06	0.13	8.95
Leys et al. 2012 [13]	Mix	>18<	15.0	2.00	0.62	6.48
Rahardja et al. [14]	NR	>18<	1.9	1.91	1.44	2.53
Salmon et al. [15]	Hamstring	>18<	19.7	3.95	1.80	8.67
Shelbourne et al. 2009 [16]	Patella	>18<	5.0	5.55	3.07	10.04
Thompson et al. 2017 [17]	Patella	>18<	20.0	1.10	0.11	10.89
Yabroudi et al. 2016 [18]	NR	>18<	3.4	9.52	2.05	44.23
				3.03	1.74	5.27
Gifstad et al. 2014 [19]	Mix	>20<	5.0	2.27	2.02	2.55
Lee et al. 2021 [20]	Hamstring	>20<	3.0	4.27	0.46	39.63
Magnussen et al. 2012 [21]	Hamstring	>20<	1.2	22.67	2.97	173.11
Wasserstein et al. 2013 [22]	NR	>20<	5.2	3.46	2.79	4.31
Webster et al. 2014 [23]	NR	>20<	4.8	6.32	2.81	14.18
				3.53	2.29	5.45
Barrett et al 2011 [24]	Patella	>25<	5.2	2.19	1.18	4.07
Cooper et al. 2018 [25]	Patella	>25<	2.0	3.60	1.62	8.00
Kamien et al. 2013 [26]	Hamstring	>25<	2.0	5.22	1.37	19.90
Rahardja et al. 2020 [14]	NR	>25<	1.9	2.05	1.59	2.63
Wasserstein et al. 2013 [22]	NR	>25<	5.0	3.70	2.93	4.68
				2.86	1.97	4.16
Mardani-Kivi et al. 2019 [27]	[illegible]nstring	>30<	6.5	1.57	0.78	3.16
Rahardja et al. 2020 [14]	NR	>30<	1.9	2.52	1.84	3.44
Runer et al. 2020 [12]	Mix	>30<	2.0	5.65	2.18	14.61
				2.59	1.51	4.44
Annear et al. 2019 [28]	Hamstring	Continuous	10.0	0.12	0.02	0.72
Beischer et al. 2020 [3]	Mix	Continuous	1.5	0.45	0.06	3.55
Benner et al. 2016 [29]	Patella	Continuous	4.1	0.37	0.17	0.80
Ferring et al. 2013 [5]	Mix	Continuous	3.0	0.30	0.07	1.35
Filbay et al. 2017 [4]	Mix	Continuous	5.0	0.19	0.03	1.15
Gupta et al. 2020 [30]	Mix	Continuous	10.0	0.51	0.14	1.84
Henle et al. 2018 [6]	LARS	Continuous	2.5	0.16	0.08	0.31
Henle et al. 2018 [7]	LARS	Continuous	2.0	0.98	0.89	1.08
Ifran et al. 2020 [31]	Mix	Continuous	2-7	0.90	0.84	0.97
Kaeding et al. 2017 [1]	Mix	Continuous	2.0	0.28	0.20	0.40
Kajetanek et al. 2017 [8]	Hamstring	Continuous	2.2	0.25	0.11	0.57
Kim et al. 2020 [32]	NR	Continuous	4.1	0.76	0.36	1.60
King et al. 2020 [2]	Mix	Continuous	2.4	0.37	0.21	0.67
Krismer et al. 2017 [9]	LARS	Continuous	2.0	0.24	0.11	0.51
Kyritsis et al. 2016 [33]	Mix	Continuous	2.0	1.54	0.72	3.32
Laboute et al. 2010 [34]	Mix	Continuous	3.5	0.62	0.30	1.28
Maletis et al. 2015 [35]	Mix	Continuous	2.4	0.81	0.68	0.96
Okoroha et al. 2019 [36]	NR	Continuous	NR	0.88	0.39	2.02
Poston et al. 2021 [37]	Hamstring	Continuous	2.0	0.29	0.21	0.39
Pullen et al. 2016 [38]	NR	Continuous	7.0	0.56	0.48	0.65
Rahardja et al. 2020 [14]	NR	Continuous	1.9	0.45	0.36	0.56
Riff et al. 2017 [39]	Mix	Continuous	15.7	0.99	0.77	1.27
Singhal et al. 2007 [40]	Tibialis	Continuous	4.5	0.21	0.07	0.61
Tagesson et al. 2020 [10]	Hamstring	Continuous	5.0	0.85	0.09	8.13
Van Eck et al. 2012 [41]	Hamstring	Continuous	2.5	0.32	0.14	0.71
Wasserstein et al. 2013 [22]	NR	Continuous	5.2	0.29	0.24	0.36
				0.47	0.38	0.59

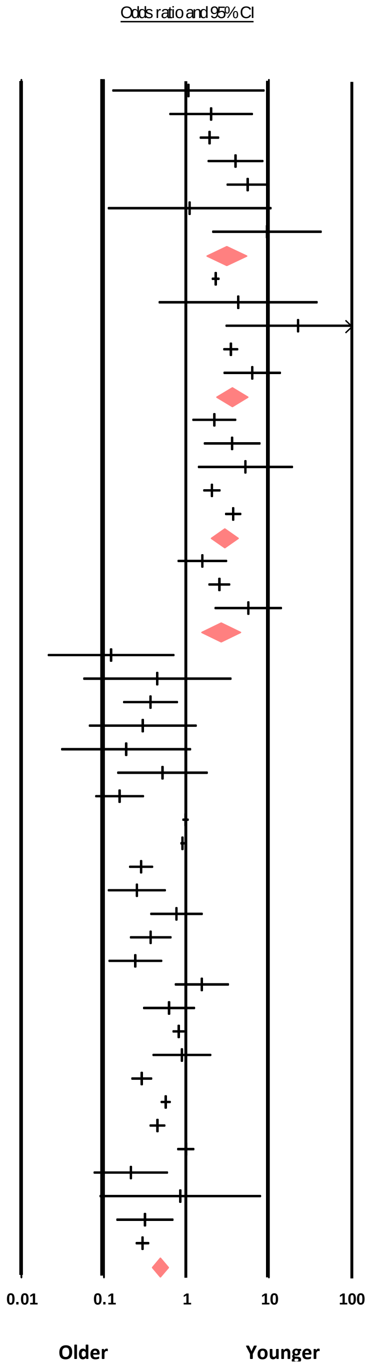

I^2 statistics
>18<: 62%, >20<: 69%, >25<: 85%
>30<: 56%, continuous: 92%

Figure 2. Meta-analysis on the association between age and graft rupture (>18<; graft ruptures n = 413, controls n = 9318), (>20<; graft ruptures n = 1579, controls n = 57615), (>25<; graft ruptures n = 697, controls n = 21368), (>30<; graft ruptures n = 329, controls n = 8936), (continuous; graft ruptures n = 2450, controls n = 66451). Mix = mix of different grafts, LARS = Ligament Advanced Reinforcement System, Follow-up = years.

Lateral tibial slope

Author & year	Population	Graft	Follow-up	Odds ratio	Lower limit	Upper limit
*Christen et al. 2015 [42]	A/C&A	Mix	6.9	1.98	0.65	5.97
Christensen et al. 2015 [42]	A/C&A	Mix	6.9	5.96	1.44	24.74
Digiacomo et al. 2018 [43]	A/C&A	NR	4.0	0.77	0.20	2.97
Grassi et al. 2019 [44]	A/C&A	Hamstring	3.0	26.58	10.70	66.03
Jaecker et al. 2018 [45]	A/C&A	Mix	NR	2.99	1.56	5.72
Kajetanek et al. 2017 [8]	A/C&A	Hamstring	2.2	0.60	0.20	1.81
Mitchell et al. 2021 [46]	A/C&A	Mix	3.5	1.94	1.00	3.77
*Sauer et al. 2019 [47]	A/C&A	Hamstring	4.0	1.40	1.05	1.87
Sauer et al. 2019 [47]	A/C&A	Hamstring	4.0	1.32	0.52	3.30
Webb et al. 2013 [48]	A/C&A	Hamstring	15.0	1.76	0.86	3.59
				2.21	1.26	3.86

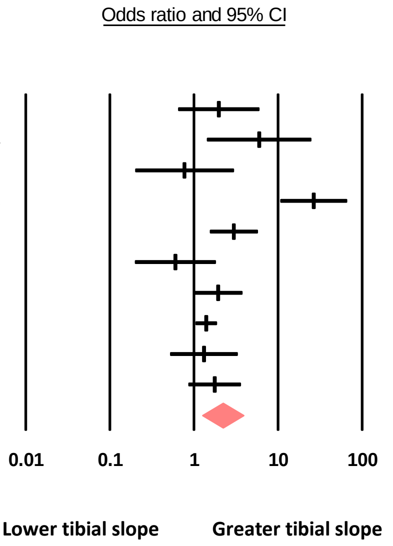

I^2 statistics: 81%

Figure 3. Meta-analysis on the association between lateral tibial slope and graft rupture (graft ruptures n = 311, controls n = 545). A = adults, C&A = children/adolescents, NR = not reported, Mix = mix of different grafts, PTS = posterior tibial slope, Follow-up = years,*Christenssen et al. reported the result for two cohorts (men vs women) and Sauer et al reported the result for two cohorts with two types of graft positioning.

Psychological readiness to return to sport

Author & year	Population	Graft	Follow-up	Statistics for each study: Odds ratio	Lower limit	Upper limit
King et al. 2021 [49]	A/C&A	Mix	2.0	2.02	0.91	4.51
McPherson et al. 2019 [50]	A/C&A	Mix	2.0	2.29	1.20	4.37
				2.18	1.32	3.61

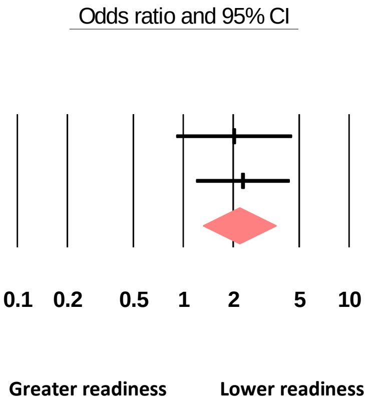

I² statistics: <0.001%

Figure 4. Meta-analysis on the association between psychological readiness to return to sport and graft rupture (graft ruptures n = 65, controls n = 352). A = adults, C&A = children/adolescents, Mix = mix of different grafts, Follow-up = years.

Timing of surgery

Author & year	Population	Graft	Months	Follow-up	Statistics for each study: Odds ratio	Lower limit	Upper limit
Fältström et al. 2021 [51]	A/C&A	Hamstring	>12<	8.0	1.92	1.60	2.31
Rahardja et al. 2014 [14]	A/C&A	NR	>12<	1.9	1.63	1.07	2.50
					1.87	1.58	2.22
Fältström et al. 2016 [51]	A/C&A	Hamstring	>3<	8.0	2.40	1.99	2.89
Grassi et al. 2020 [11]	A/C&A	Hamstring	>3<	10.0	0.42	0.05	3.48
					1.40	0.29	6.79
Rahardja et al. 2020 [14]	A/C&A	NR	>6<	1.9	1.19	0.90	1.55
Yabroudi et al. 2016 [18]	A/C&A	NR	>6<	3.4	4.21	0.55	32.29
					1.47	0.58	3.74

Odds ratio and 95% CI

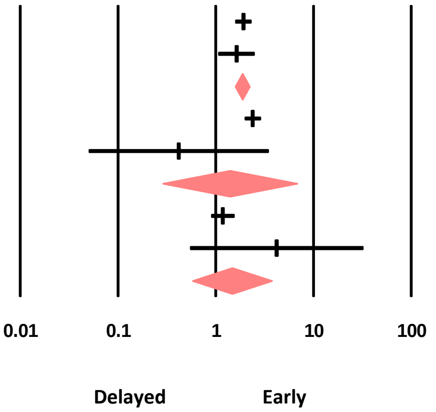

I² statistics
>12<: <0.001%, >3<: 61%, >6<: 31%

Figure 5. Meta-analysis on the association between timing of surgery and graft rupture (>12 months<; graft ruptures n = 910, controls n = 24091), (>3 months<; graft ruptures n = 661, controls n = 17853), (>6 months<; graft ruptures n = 278, controls n = 7357). NR = not reported, A = adults, C&A = children/adolescents, Follow-up = years.

Return to pre-injury activity level

Author & year	Population	Graft	Follow-up	Odds ratio	Lower limit	Upper limit
Allen et al. 2016 [52]	A/C&A	Mix	5.7	8.58	0.48	152.49
Bodkin et al. 2021 [53]	A/C&A	Patella	2.0	3.21	0.72	14.22
Bourke et al. 2012 [54]	A/C&A	Mix	16.0	0.94	0.55	1.63
Salmon et al. 2005 [55]	A/C&A	Mix	5.0	1.82	0.90	3.67
Sandon et al. 2020 [56]	A/C&A	Mix	10.0	2.91	1.48	5.71
Webster et al. 2014 [23]	A/C&A	NR	4.8	3.90	1.33	11.48
Welling et al. 2020 [57]	A/C&A	Mix	2.1	0.61	0.13	2.87
Yaborudi et al. 2016 [18]	A/C&A	NR	3.4	2.00	0.45	8.93
				1.91	1.17	3.12
Britt et al. 2020 [58]	C&A	Mix	3.4	4.03	0.47	34.80
Fones et al. 2020 [59]	C&A	Mix	4.0	0.58	0.12	2.68
Morgan et al. 2016 [60]	C&A	Mix	16.5	2.43	0.90	6.52
				1.72	0.61	4.80
			**Overall**	1.87	1.21	2.91

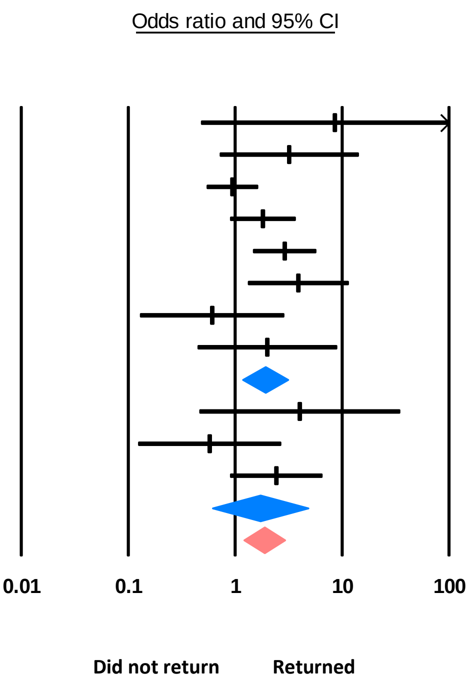

I² statistics: 37%

Figure 6. Meta-analysis on the association between return to pre-injury activity level and graft rupture (graft ruptures n = 302, controls n = 3224). NR = not reported, A = adults, C&A = children/adolescents. Blue color = sub group total, pink color = Overall, irrespective of subgroup

Family history

Author & year	Population	Graft	Follow-up	Statistics for each study: Odds ratio	Lower limit	Upper limit
Bourke et al. 2012 [54]	A/C&A	Mix	16.0	1.82	1.07	3.08
Goshima et al. 2014 [61]	A/C&A	NR	2.0	1.77	0.46	6.87
Mardani-Kivi et al. 2019 [27]	A/C&A	Hamstring	6.5	0.98	0.40	2.40
Salmon et al. 2005 [55]	A/C&A	Mix	5.0	1.40	0.64	3.05
Salmon et al. 2018 [15]	A/C&A	Hamstring	19.7	1.80	0.80	4.08
Thompson et al. 2015 [17]	A/C&A	Patella	20.0	2.00	0.49	8.10
Webster et al. 2014 [23]	A/C&A	NR	4.8	2.33	1.03	5.28
				1.69	1.24	2.29
Bram et al. 2020 [62]	C&A	NR	4.3	1.22	0.66	2.28
Ghosh et al. 2020 [63]	C&A	Hamstring	2.0	3.31	0.77	14.17
Heat et al. 2019 [64]	C&A	Hamstring	4.5	1.54	0.72	3.31
Morgan et al. 2016 [60]	C&A	Mix	16.5	3.71	1.84	7.47
				2.03	1.13	3.64
			**Overall**	1.76	1.34	2.31

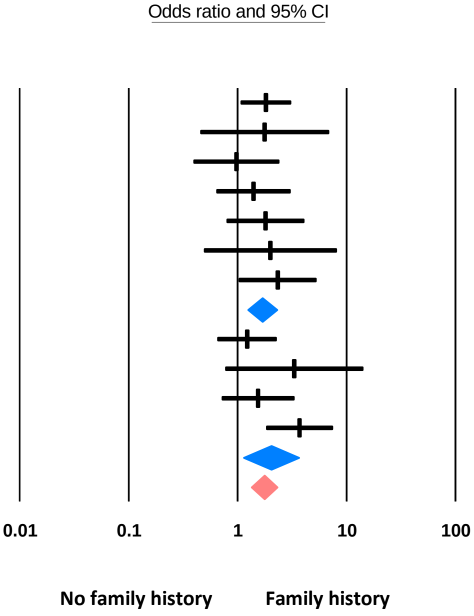

I² statistics: <0.001%

Figure 7. Meta-analysis on the association between family history and graft rupture (graft ruptures n = 357, controls n = 3837). ACL = anterior cruciate ligament, NR = not reported, Mix = mix of different grafts, A = adults, C&A = children/adolescents. Blue color = sub group total, pink color = overall, irrespective of subgroup

KOOS

Author & year	Population	Graft	KOOS	Follow-up	Odds ratio	Lower limit	Upper limit
Fältström et al. 2016 [51]	A/C&A	Hamstring	ADL	8.0	1.03	0.88	1.22
Tagesson et al. 2016 [10]	A/C&A	Hamstring	ADL	5.0	0.27	0.03	2.74
					0.89	0.39	2.02
Fältström et al. 2016 [51]	A/C&A	Hamstring	Pain	8.0	0.95	0.81	1.12
Tagesson et al. 2016 [10]	A/C&A	Hamstring	Pain	5.0	0.65	0.07	6.26
					0.95	0.80	1.12
Fältström et al. 2016 [51]	A/C&A	Hamstring	QoL	8.0	1.01	0.86	1.19
Tagesson et al. 2016 [10]	A/C&A	Hamstring	QoL	5.0	0.47	0.05	4.59
					1.01	0.85	1.19
Fältström et al. 2016 [51]	A/C&A	Hamstring	Sport/rec	8.0	0.96	0.82	1.13
Tagesson et al. 2016 [10]	A/C&A	Hamstring	Sport/rec	5.0	0.60	0.06	5.83
					0.96	0.81	1.13
Fältström et al. 2016 [51]	A/C&A	Hamstring	Symptoms	8.0	0.81	0.69	0.96
Tagesson et al. 2016 [10]	A/C&A	Hamstring	Symptoms	5.0	0.29	0.03	2.89
					0.81	0.69	0.95

Odds ratio and 95% CI

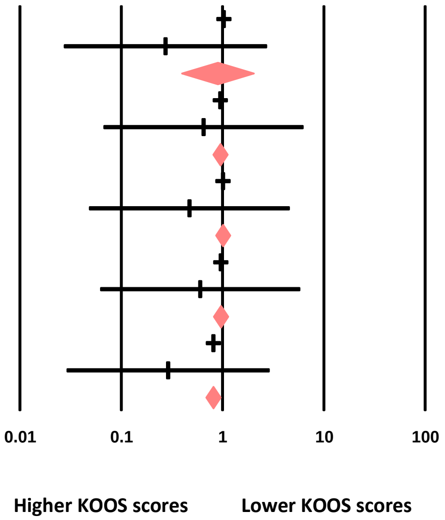

I^2 statistics
ADL: 21%, Pain: <0.001%, QoL: <0.001%, Sport/rec: <0.001%, Symptoms: <0.001%

Figure 8. Meta-analysis on the association between Knee injury and Osteoarthritis Outcome Scores (KOOS) and graft rupture (graft ruptures n = 486, controls n = 13207). ADL=Activity of Daily Living, QoL=Quality of Life, A = adults, C&A = Children/adolescents, Follow-up = years

Concomitant cartilage injury

Author & year	Population	Graft	Follow-up	Odds ratio	Lower limit	Upper limit
Everhart et al. 2020 [65]	A/C&A	Mix	3.2	0.59	0.27	1.28
Filbay et al. 2017 [4]	A/C&A	Mix	5.0	0.87	0.09	8.74
Fleming et al. 2013 [5]	A/C&A	Mix	3.0	0.81	0.09	7.45
Gifstad et al. 2019 [19]	A/C&A	Mix	5.0	0.68	0.59	0.80
Kim et al. 2020 [32]	A/C&A	NR	4.1	0.94	0.36	2.50
King et al. 2020 [2]	A/C&A	Mix	2.4	0.73	0.26	2.10
Lord et al. 2020 [66]	A/C&A	Mix	2.0	0.55	0.32	0.92
Rahardja et al. 2020 [14]	A/C&A	NR	1.9	0.74	0.57	0.96
Webster et al. 2014 [23]	A/C&A	NR	4.8	1.24	0.54	2.84
				0.70	0.62	0.79

Odds ratio and 95% CI

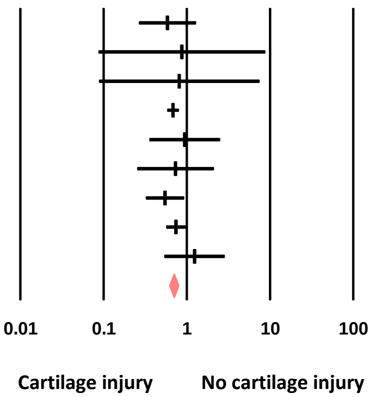

I^2 statistics: < 0.001%

Figure 9. Meta-analysis on the association between concomitant cartilage injury and graft rupture (graft ruptures n = 1740, controls n = 62038). NR = not reported, Mix = mix of different grafts, A = adults, C&A = children/adolescents, Follow-up = year

Return to sports - a risky business? A systematic review with meta-analysis of risk factors for graft rupture following ACL reconstruction, Sports Medicine, Anna Cronström; anna.cronstrom@umu.se, Eva Tengman, Charlotte Häger, Umeå University

Sex

Author & year	Population	Graft	Follow-up	Odds ratio	Lower limit	Upper limit
Borton et al. 2018 [67]	A	Hamstring	4.0	1.56	0.44	5.47
Lee et al. 2017 [68]	A	Mx	2.5	1.07	0.11	10.97
Pullen et al. 2016 [38]	A	NR	7.0	0.89	0.71	1.12
Salmon et al. 2018 [15]	A	Hamstring	19.7	0.98	0.40	2.41
Shelbourne et al. 1998 [69]	A	Patella	NR	0.85	0.30	2.45
Tulloch et al. 2019 [70]	A	LARS	7.8	0.96	0.30	3.07
Annear et al. 2019 [28]	A/C&A	Hamstring	10.0	0.72	0.10	5.01
Beischehr et al. 2020 [3]	A/C&A	Mx	1.5	1.39	0.38	5.02
Bodkin et al. 2021 [53]	A/C&A	Mx	2.0	0.25	0.09	0.71
Bourke et al. 2012 [54]	A/C&A	Mx	16.0	0.45	0.25	0.81
Cooper et al. 2018 [25]	A/C&A	Patella	2.0	0.65	0.28	1.54
Csapo et al. 2021 [71]	A/C&A	Mx	2.0	3.30	0.41	26.37
Fleming et al. 2013 [5]	A/C&A	Mx	3.0	1.82	0.32	10.47
Gifstad et al. 2014 [19]	A/C&A	Mx	5.0	1.12	1.00	1.25
Grassi et al. 2020 [11]	A/C&A	Hamstring	10.0	1.07	0.21	5.46
Gupta et al. 2020 [30]	A/C&A	Patella	10.0	1.75	0.21	14.83
Henle et al. 2018 [6]	A/C&A	LARS	2.5	0.83	0.31	2.21
Henle et al. 2018 [7]	A/C&A	LARS	2.0	0.38	0.10	1.51
Ifran et al. 2020 [31]	A/C&A	Mx	7.0	1.20	0.54	2.62
Kaeding et al. 2017 [1]	A/C&A	Mx	2.0	0.87	0.59	1.28
Kajatenak et al. 2017 [8]	A/C&A	Hamstring	2.2	1.17	0.47	2.89
Kamien et al. 2013 [26]	A/C&A	Hamstring	2.0	0.76	0.36	1.57
Kim et al. 2020 [32]	A/C&A	NR	4.1	2.47	0.89	6.86
King et al. 2020 [2]	A/C&A	Mx	2.4	0.73	0.30	1.77
Krismer et al. 2017 [9]	A/C&A	LARS	2.0	0.64	0.27	1.55
Laboute et al. 2010 [34]	A/C&A	Mx	3.5	0.86	0.31	2.38
Larson et al. 2017 [72]	A/C&A	Mx	6.0	1.07	0.43	2.67
Leys et al. 2013 [13]	A/C&A	Mx	15.0	0.31	0.11	0.89
Lord et al. 2020 [66]	A/C&A	Hamstring	2.0	1.09	0.78	1.51
Magnussen et al. 2012 [21]	A/C&A	Hamstring	1.2	1.86	0.70	4.96
Maletis et al. 2015 [35]	A/C&A	Mx	2.4	1.08	0.88	1.32
Mardani-Kivi et al. 2019 [27]	A/C&A	Hamstring	6.5	2.18	1.05	4.51
McPherson et al. 2019 [50]	A/C&A	Mx	2.0	0.76	0.35	1.66
Mohtadi et al. 2016 [73]	A/C&A	Mx	2.0	0.50	0.17	1.46
Paterno et al. 2012 [74]	A/C&A	NR	1.0	2.11	0.22	20.12
Perkins et al. 2019 [75]	A/C&A	Hamstring	2.0	0.97	0.53	1.75
Pinczewski et al. 2007 [76]	A/C&A	Mx	10.0	1.86	0.87	3.96
poston et al. 2007 [37]	A/C&A	Mx	2.0	1.39	0.24	8.05
Rahardja et al. 2020 [14]	A/C&A	NR	1.9	0.59	0.45	0.77
Riff et al. 2017 [39]	A/C&A	Mx	15.7	1.15	0.87	1.52
Rosenstiel et al. 2019 [77]	A/C&A	Hamstring	3.9	7.42	0.73	75.90
Runer et al. 2020 [12]	A/C&A	Mx	2.0	0.54	0.26	1.12
Salmon et al. 2005 [55]	A/C&A	Mx	5.0	0.48	0.22	1.03
Salmon et al. 2006 [78]	A/C&A	Patella	13.0	1.21	0.27	5.39
Sanders et al. 2017[79]	A/C&A	Mx	10.0	0.92	0.56	1.49
Schlumberger et al. 2017 [80]	A/C&A	Mx	5.0	0.44	0.25	0.78
Shelbourne et al. 2009 [16]	A/C&A	Patella	5.0	1.17	0.70	1.97
Sousa et al. 2017 [81]	A/C&A	Mx	4.0	1.90	1.17	3.09
Tagesson et al. 2016 [10]	A/C&A	Hamstring	5.0	0.88	0.06	12.97
Thompson et al. 2015 [17]	A/C&A	Patella	20.0	0.13	0.02	1.10
Van Eck et al. 2012 [41]	A/C&A	Hamstring	2.5	0.77	0.34	1.75
Wasserstein et al. 2013 [22]	A/C&A	NR	5.2	1.31	1.05	1.63
Webb et al. 2013 [48]	A/C&A	Hamstring	15.0	1.28	0.59	2.78
Webster et al. 2014 [23]	A/C&A	NR	4.8	0.70	0.29	1.70
Webster et al. 2021 [82]	A/C&A	Hamstring	2.0	0.40	0.19	0.85
Wright et al. 2007 [83]	A/C&A	Mx	2.0	0.18	0.02	1.54
Yabroudi et al. 2016 [18]	A/C&A	NR	3.4	0.54	0.25	1.15
Bayomy et al. 2019 [84]	C&A	Hamstring	2.8	1.16	0.19	6.97
Bram et al. 2020 [62]	C&A	NR	4.3	0.55	0.30	1.00
Cruz et al. 2015 [85]	C&A	Mx	1.8	1.00	0.44	2.29
Ellis et al. 2012 [86]	C&A	Patella	4-11	0.42	0.09	2.03
Engelman et al. 2014 [87]	C&A	Hamstring	4.2	0.94	0.41	2.17
Fones et al. 2020 [59]	C&A	Mx	4.0	0.57	0.13	2.49
Ghosh et al. 2020 [63]	C&A	Hamstring	2.0	0.60	0.20	1.82
Graziano et al. 2017 [80]	C&A	Hamstring	NR	0.82	0.08	8.75
Heath et al. 2019 [64]	C&A	Hamstring	4.5	0.84	0.44	1.61
Ho et al. 2018 [89]	C&A	Mx	1.0	1.05	0.60	1.84
Larson et al. 2016 [90]	C&A	Mx	4.0	3.69	0.36	37.86
Morgan et al. 2016 [60]	C&A	Mx	16.5	0.56	0.27	1.15
Rauck et al. 2021 [91]	C&A	Patella	3.8	1.20	0.11	12.54
Salmon et al. [15]	C&A	Hamstring	19.7	0.38	0.10	1.50
Scmale et al. 2014[92]	C&A	Mx	4.0	0.75	0.06	8.83
Wall et al. 2017 [93]	C&A	Hamstring	3.6	3.50	0.24	51.46
Webster et al. 2021 [94]	C&A	Mx	3.4	0.46	0.26	0.81
				0.88	0.79	0.98

Odds ratio and 95% CI

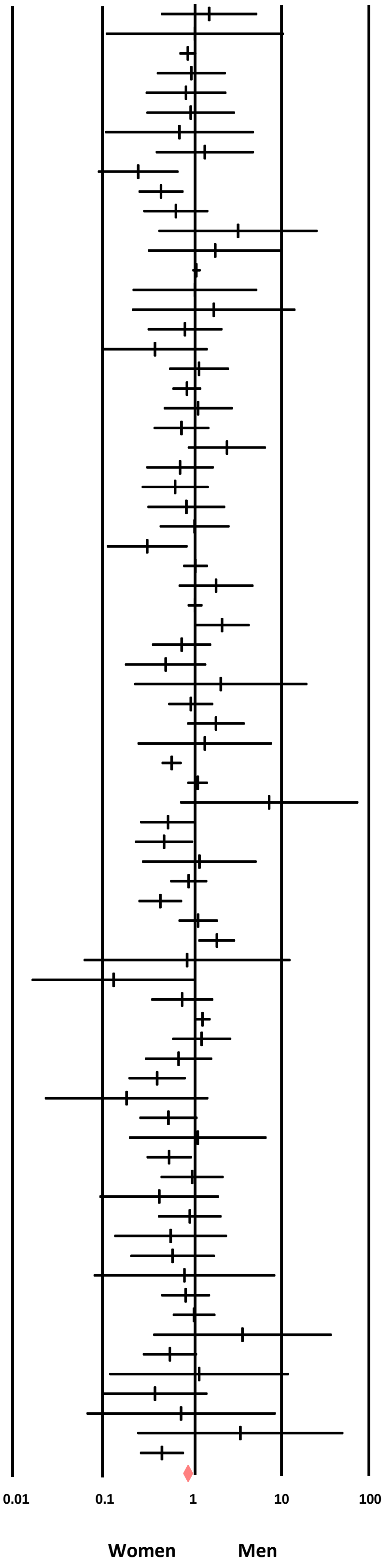

I² statistics: 42%

Return to sports - a risky business? A systematic review with meta-analysis of risk factors for graft rupture following ACL reconstruction, Sports Medicine, Anna Cronström; anna.cronstrom@umu.se, Eva Tengman, Charlotte Häger, Umeå University

Figure 10. Meta-analysis on the association between sex and graft (graft ruptures n = 4817, controls n = 128 377). NR = not reported, Mix = mix of different grafts, LARS = Ligament Advanced Reinforcement System, A = adults, C&A = children/adults, Follow-up = years.

BMI

Author & year	Population	Graft	BMI	Follow-up	Odds ratio	Lower limit	Upper limit
Grassi et al. 2020 [11]	A/C&A	Hamstring	>25<	10.0	1.37	0.27	6.95
Mardani-Kivi et al. 2019 [27]	A/C&A	Hamstring	>25<	6.5	2.32	1.09	4.93
Pullen et al. 2016 [38]	A/C&A	NR	>25<	7.0	0.69	0.55	0.88
					1.22	0.48	3.11
Cruz et al. 2017 [85]	C&A	Mix	Continuous	1.8	1.70	0.55	5.26
Filbay et al. 2017 [4]	A/C&A	Mix	Continuous	5.0	0.30	0.05	1.83
Henle et al. 2018 [6]	A/C&A	LARS	Continuous	2.5	1.06	0.91	1.24
Ifran et al. 2020 [31]	A/C&A	Mix	Continuous	2.0	0.97	0.89	1.05
Kaeding et al. 2017 [1]	A/C&A	Mix	Continuous	2.0	0.54	0.46	0.62
Kim et al. 2020 [32]	A/C&A	NR	Continuous	4.1	1.77	0.84	3.73
Krismer et al. 2017 [9]	A/C&A	LARS	Continuous	2.0	1.10	0.52	2.33
Maletis et al. 2015 [35]	A/C&A	Mix	Continuous	2.4	0.96	0.39	2.34
Perkins et al. 2019 [75]	C&A	Hamstring	Continuous	2.0	0.62	0.36	1.07
Pullen et al. 2016 [38]	A/C&A	NR	Continuous	7.0	0.91	0.79	1.06
Van Eck et al. 2012 [41]	A/C&A	Hamstring	Continuous	2.5	0.47	0.22	0.98
Yabroudi et al. 2016 [18]	A/C&A	NR	Continuous	3.4	0.96	0.86	1.07
					0.87	0.73	1.03

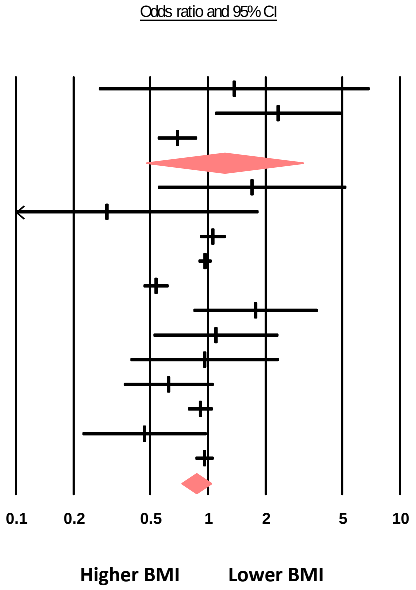

I^2 statistics
>25<: 78%, continuous: 83%

Figure 11. Meta-analysis on the association between Body Mass Index (BMI) and graft rupture. ≥25 vs. <25 (graft ruptures n = 664, controls n = 17595), continuous: (graft ruptures n = 1740, controls n = 62038). NR = not reported, Mix = mix of different grafts, LARS = Ligament Advanced Reinforcement System, A = adults, C&A = children/adolescents, Follow-up = years.

Smoking status

Author & year	Population	Graft	Follow-up	Odds ratio	Lower limit	Upper limit
Filbay et al. 2017 [4]	A/C&A	Mix	5.0	0.88	0.09	8.77
Grassi et al. 2020 [11]	A/C&A	Hamstring	10.0	1.37	0.32	5.87
Henle et al. 2018 [6]	A/C&A	LARS	2.5	1.21	0.48	3.09
Kaeding et al. 2017 [1]	A/C&A	Mix	2.0	0.31	0.15	0.65
Pullen et al. 2016 [38]	A	NR	7.0	0.90	0.76	1.06
				0.77	0.45	1.31

Statistics for each study; Odds ratio and 95% CI

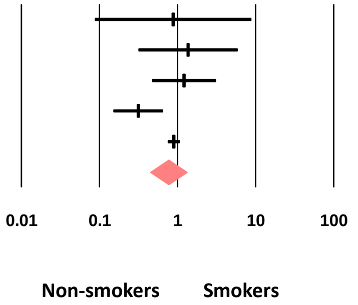

I^2 statistics: 74%

Figure 12. Meta-analysis on the association between smoking status and graft rupture (graft ruptures n = 741, controls n = 9969). NR = not reported, Mix = mix of different grafts, LARS = Ligament Advanced Reinforcement System, A = adults, C&A = children/adolescents, Follow-up = years.

Injury mechanism

Author & year	Population	Graft	Follow-up	Odds ratio	Lower limit	Upper limit
King et al. 2020 [2]	A/C&A	Mix	2.4	1.29	0.68	2.47
Laboute et al. 2010 [34]	A/C&A	Mix	3.5	0.38	0.11	1.31
Salmon et al. 2005 [55]	A/C&A	Mix	5.0	3.48	1.79	6.79
Webster et al. 2014 [23]	A/C&A	Mix	5.0	3.24	1.44	7.28
				1.70	0.75	3.87

Statistics for each study; Odds ratio and 95% CI

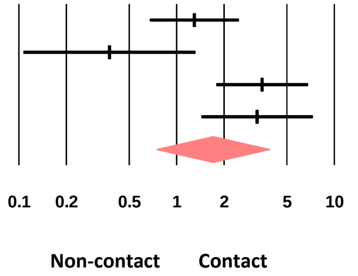

I^2 statistics: 53%

Figure 13. Meta-analysis on the association between injury mechanism and graft rupture (graft ruptures n = 123, controls n = 2647). Mix = mix of different grafts, A = adults, C&A = children/adolescents, Follow-up = years.

Medial tibial slope

Author & year	Population	Graft	Tibial slope	Follow-up	Odds ratio	Lower limit	Upper limit
					Statistics for each study		
Digiacomo et al. 2018 [43]	A/C&A	NR	Medial (PTS)	4.0	1.38	0.36	5.30
Grassi et al. 2019 [44]	A/C&A	Hamstring	Medial (PTS)	3.0	6.04	2.68	13.61
Grassi et al. 2021 [95]	C&A	Hamstring	Medial (ATS)	>2	0.45	0.05	4.00
Grassi et al. 2021 [95]	C&A	Hamstring	Medial (CTS)	>2	0.61	0.07	5.31
Grassi et al. 2021 [95]	C&A	Hamstring	Medial (PTS)	>2	0.55	0.06	4.80
Jaecker et al. 2018 [45]	A/C&A	Mix	Medial (PTS)	NR	2.99	1.56	5.72
Kajetanek et al. 2017 [8]	A/C&A	Hamstring	Medial (PTS)	2.2	0.25	0.08	0.78
Kim et al. 2020 [32]	A/C&A	NR	Medial (PTS)	4.1	0.74	0.35	1.55
Lee et al. 2018 [96]	A	NR	Medial (PTS)	3.1	2.31	1.22	4.37
Su et al. 2020 [97]	A/C&A	NR	Medial (ATS)	NR	1.00	0.31	3.19
Su et al. 2020 [97]	A/C&A	NR	Medial (CTS)	NR	1.31	0.41	4.18
Su et al. 2020 [97]	A/C&A	NR	Medial (PTS)	NR	1.53	0.48	4.92
					1.31	0.76	2.24

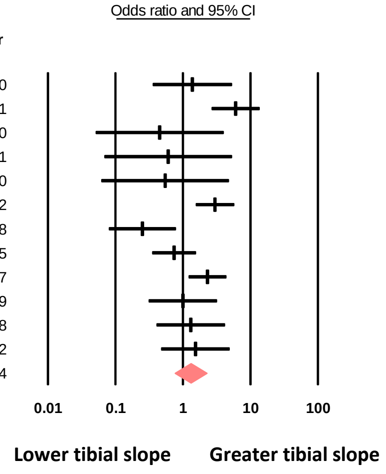

I^2 statistics: 67%

Figure 14. Meta-analysis on the association between medial tibial slope and graft rupture (graft ruptures n = 123, controls n = 2647). NR = not reported, Mix = mix of different grafts, PTS = posterior tibial slope, ATS = anterior tibial slope, CTS = central tibial slope, A = adults, C&A = children/adolescents, Follow-up = years.

General joint laxity

Author & year	Population	Graft	Follow-up	Odds ratio	Lower limit	Upper limit
				Statistics for each study		
Akhtar et al. 2016 [98]	A/C&A	Mix	4.0	9.50	2.80	32.18
Larson et al. 2017 [72]	A/C&A	Mix	16.0	1.04	0.27	3.98
				3.20	0.37	27.93

Odds ratio and 95% CI

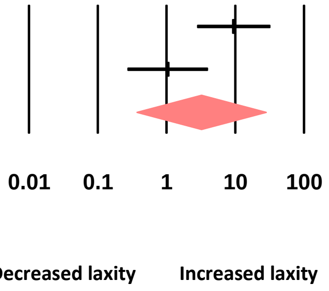

I² statistics: 82%

Figure 15. Meta-analysis on the association between general joint laxity and graft rupture (graft ruptures n = 57, controls n = 309). Mix = mix of different grafts, A = adults, C&A = children/adolescents, Follow-up = years.

Number of physical therapy visits

Author & year	Population	Graft	Follow-up	Odds ratio	Lower limit	Upper limit
				Statistics for each study		
Filbay et al. 2017 [4]	A	Mix	5.0	1.85	0.30	11.32
Vincent et al. 2017 [99]	A/C&A	Patella	5.0	0.93	0.24	3.55
				1.19	0.40	3.48

Odds ratio and 95% CI

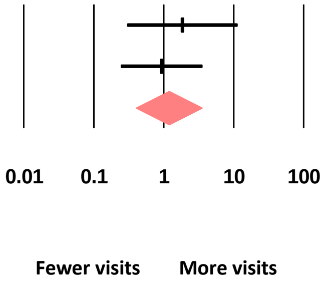

I² statistics: <0.001%

Figure 16. Meta-analysis on the association between the number of physical therapy visits and graft rupture (graft ruptures n = 13, controls n = 142). A = adults, C&A = children/adolescents, Mix = mix of different grafts, Follow-up = years.

Timing of return to sport

Author & year	Population	Graft	Follow-up	Odds ratio	Lower limit	Upper limit
Shelbourne et al. 2009 [16]	A/C&A	Patella	5.0	1.16	0.69	1.95
Sousa et al. 2017 [81]	A/C&A	Mix	4.0	0.82	0.17	3.96
				1.12	0.68	1.83

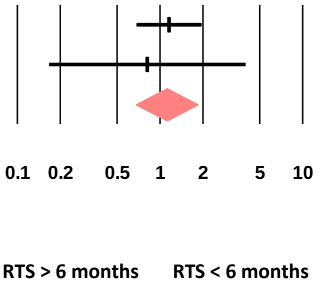

I^2 statistics: <0.001%

Figure 17. Meta-analysis on the association between timing of return to sport (RTS) and graft rupture (>6< months) (graft ruptures n = 71, controls n = 1467). A = adults, C&A = children/adolescents, Mix = mix of different graft, Follow-up = years.

Soccer vs. other sports

Author & year	Population	Graft	Follow-up	Odds ratio	Lower limit	Upper limit
Allen et al. 2016 [52]	A/C&A	Mix	5.7	11.13	1.39	88.84
Gifstad et al. 2014 [19]	A/C&A	Mix	5.0	1.12	1.00	1.27
Kaeding et al. 2017 [1]	A/C&A	Mix	2.0	1.57	0.92	2.67
Kyritsis et al. 2016 [33]	A/C&A	Mix	2.0	0.60	0.22	1.63
Rosenstiel et al. 2019 [77]	A/C&A	Hamstring	3.9	3.83	0.38	38.75
				1.32	0.82	2.15

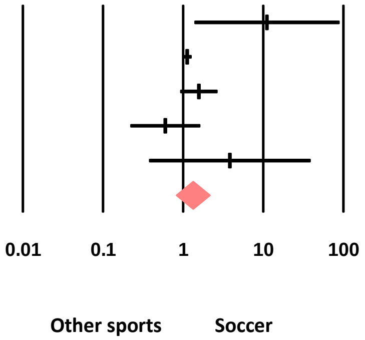

I^2 statistics: 54%

Figure 18. Meta-analysis on the association between playing soccer and graft rupture (graft ruptures n = 1248, controls n = 43422). A = adults, C&A = children/adolescents, Mix = mix of different grafts, Follow-up = years.

Hop performance

Author & year	Population	Graft	Task	Follow-up	Odds ratio	Lower limit	Upper limit
Fältström et al. 2021 [100]	A/C&A	NR	SLHD	2.0	0.63	0.27	1.50
King et al. 2021 [49]	A/C&A	Mix	SLHD	2.0	0.99	0.45	2.18
Kyritsis et al. 2016 [33]	A/C&A	Mix	SLHD	2.0	1.86	0.86	4.00
Webster et al. 2019 [101]	A/C&A	Hamstring	SLHD	5.0	0.84	0.48	1.47
					0.99	0.65	1.50
Kyritsis et al. 2016 [33]	A/C&A	Mix	THD	2.0	1.32	0.61	2.82
Webster et al. 2019 [101]	A/C&A	Hamstring	THD	5.0	1.08	0.62	1.87
					1.15	0.74	1.81

(Statistics for each study: Odds ratio, Lower limit, Upper limit)

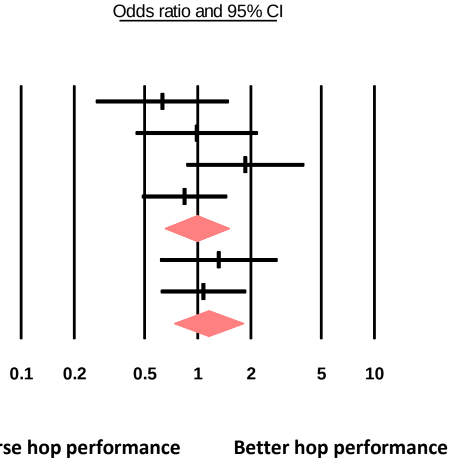

I^2 statistics
SLHD: 24%
THD: <0.001%

Figure 19. Meta-analysis on the association between hop performance and graft rupture (SLHD: graft ruptures n = 128, controls n = 571, THD, respectively: graft ruptures n = 76, controls n = 366). A = adults, C&A = children/adolescents, Mix = mix of different grafts, SLHD = single leg hop for distance, THD = Triple hop for distance. Follow-up = years.

Muscle strength

Author & year	Population	Graft	Muscle	Follow-up	Odds ratio	Lower limit	Upper limit
King et al. 2021 [49]	A/C&A	Mix	Hamstring	2.0	0.74	0.34	1.64
Kyritsis et al. 2016 [33]	A/C&A	Mix	Hamstring	2.5	0.47	0.22	1.01
					0.59	0.34	1.02
King et al. 2021 [49]	A/C&A	Mix	Q-ceps	2.0	0.91	0.41	2.02
Kyritsis et al. 2016 [33]	A/C&A	Mix	Q-ceps	2.5	1.20	0.56	2.58
					1.05	0.61	1.83

(Statistics for each study: Odds ratio, Lower limit, Upper limit)

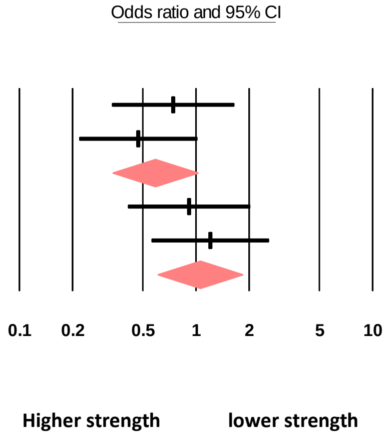

I^2 statistics: <0.001%

Figure 20. Meta-analysis on the association between muscle strength and graft rupture (Quadriseps (Q-ceps) & hamstring strength, respectively: graft ruptures n = 57, controls n = 163)

Knee abduction

Author & year	Population	Graft	Follow-up	Odds ratio	Lower limit	Upper limit
Krosshaug et al. 2016 [102]	A/C&A	NR	1-7	1.13	0.19	6.73
Poston et al. 2021 [37]	A/C&A	Hamstring	2.0	0.30	0.07	1.30
				0.53	0.15	1.91

Odds ratio and 95% CI

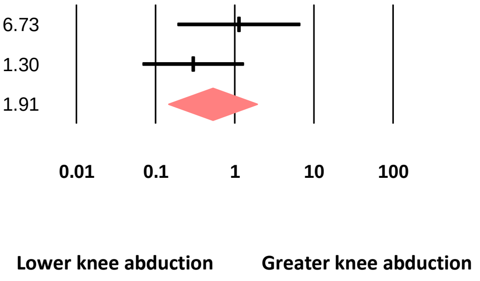

I² statistics: 21%

Figure 21. Meta-analysis on the association between knee abduction and graft rupture (graft ruptures n = 11, controls n = 655). A = adults, C&A = children/adolescents, NR = not reported, Follow-up = years.

Concomitant meniscal tear

Author & year	Population	Graft	Injury	Follow-up	Odds ratio	Lower limit	Upper limit
Fleming et al. 2013 [5]	A/C&A	Mix	Meniscal tear	3.0	1.08	0.21	5.66
Gifstad et al. 2014 [19]	A/C&A	Mix	Meniscal tear	5.0	1.01	0.90	1.14
Kaeding et al. 2017 [1]	A/C	Mix	L meniscal tear	2.0	0.94	0.63	1.40
Kim et al. 2020 [32]	A/C&A	NR	L meniscal tear	4.1	2.46	1.04	5.81
Krismer et al. 2017 [9]	A/C&A	LARS	Meniscal tear	2.0	1.53	0.57	4.05
Lord et al. 2020 [66]	A/C&A	Mix	L meniscal tear	2.0	1.19	0.82	1.72
Rahadja et al. 2014 [14]	A/C&A	NR	Meniscal tear	1.9	1.02	0.77	1.36
Salmon et al. 2005 [55]	A/C&A	Mix	Meniscal tear	5.0	2.13	0.88	5.18
					1.07	0.94	1.22
Britt et al. 2020 [58]	C&A	Mix	Meniscal tear	3.4	0.17	0.02	1.44
Fones et al. 2020 [59]	C&A	Mix	Meniscal tear	4.0	1.54	0.35	6.70
					0.59	0.07	5.06
				**Overall**	1.07	0.94	1.22

Odds ratio and 95% CI

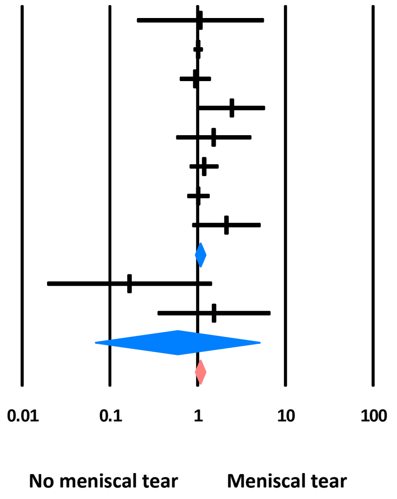

I² statistics: 17%

Figure 22. Meta-analysis on the association concomitant meniscal tear and graft rupture (graft ruptures n = 1799, controls n = 59841). A = adults, C&A = children/adolescents, NR = not reported, Mix = mix of different grafts, LARS = Ligament Advanced Reinforcement System, L = lateral, Follow-up = years. Blue color = sub group total, pink color = overall, irrespective of subgroup

Concomitant meniscal repair

Author & year	Population	Graft	Injury	Follow-up	Statistics for each study		
					**Odds ratio**	**Lower limit**	**Upper limit**
Lord et al. 2020 [66]	A/C&A	Mix	L meniscal repair	2.0	1.64	0.83	3.27
Cruz et al. 2017 [85]	A/C&A	Mix	Meniscal repair	1.8	1.24	0.61	2.52
King et al. 2020 [2]	A/C&A	Mix	Meniscal repair	2.4	0.61	0.08	4.60
Pullen et al. 2016 [38]	A/C&A	NR	Meniscal repair	7.0	0.69	0.53	0.90
					1.00	0.60	1.66

Odds ratio and 95% CI

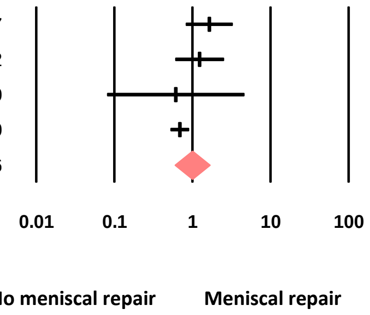

I² statistics: 56%

Figure 23. Meta-analysis on the association between concomitant meniscal repair and graft rupture (graft ruptures n = 776, controls n = 22404). A = adults, C&A = children/adolescents, Mix = mix of different grafts, L = lateral, Follow-up = years.

Concomitant meniscectomy

Author & year	Population	Graft	Injury	Follow-up	Odds ratio	Lower limit	Upper limit
Akada et al. 2019 [103]	A/C&A	Hamstring	Meniscectomy	2.0	1.14	0.23	5.73
Bourke et al. 2012 [54]	A/C&A	Mix	Meniscectomy	16.0	1.12	0.68	1.83
King et al. 2020 [2]	A/C&A	Mix	Meniscectomy	2.4	1.32	0.39	4.45
Lord 2020 [66]	A/C&A	Mix	L Meniscectomy	2.0	1.12	0.73	1.72
Salmon et al. 2005 [55]	A/C&A	Mix	Meniscectomy	5.0	1.35	0.70	2.58
Salmon et al. 2006 [78]	A/C&A	Mix	Meniscectomy	13.0	6.00	0.99	36.50
					1.21	0.92	1.60
Cruz et al. 2017 [85]	C&A	Mix	Meniscectomy	1.8	1.18	0.58	2.41
Morgan et al. 2016 [60]	C&A	Mix	Meniscectomy	16.5	0.92	0.43	1.96
Perkins et al. 2019 [75]	C&A	Hamstring	Meniscectomy	2.0	1.07	0.57	2.02
					1.06	0.71	1.58
				**Overall**	1.16	0.93	1.46

Statistics for each study; Odds ratio and 95% CI

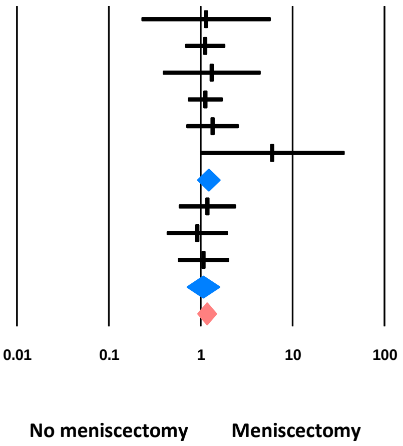

I² statistics: <0.001%

Figure 24. Meta-analysis on the association between concomitant meniscectomy and graft rupture (graft ruptures n = 373, controls n = 8906). A = adults, C&A = children/adolescents, Mix = mix of different grafts, Follow-up = years, L = lateral. Blue color = sub group total, pink color = Overall, irrespective of subgroup

Concomitant MCL injury

Author & year	Population	Graft	Injury	Follow-up	Odds ratio	Lower limit	Upper limit
Gifstad et al. 2014 [19]	A/C&A	Mix	MCL	5.0	0.92	0.64	1.33
Krismer et al. 2017 [9]	A/C&A	LARS	MCL	2.0	2.09	0.65	6.67
					1.14	0.56	2.31

Statistics for each study; Odds ratio and 95% CI

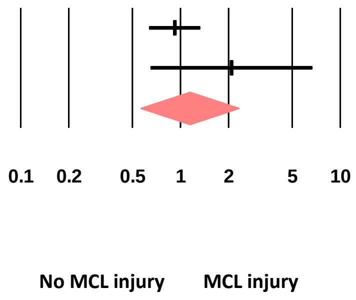

I² statistics: 42%

Figure 25. Meta-analysis on the association between concomitant medial collateral ligament (MCL) injury and graft rupture (graft ruptures n = 1223, controls n = 44443). A = adults, C&A = children/adolescents, Mix = mix of different grafts, Follow-up = years.

Femoral condyle ratio

Author & year	Population	Graft	Follow-up	Odds ratio	Lower limit	Upper limit
Grassi et al. 2019 [44]	A/C&A	Hamstring	3.0	1.69	0.78	3.66
Pfeiffer et al. 2018 [104]	A/C&A	NR	4.4	0.91	0.48	1.72
				1.19	0.65	2.19

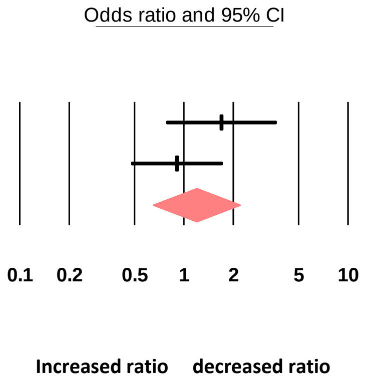

I² statistics: 33%

Figure 26. Meta-analysis on the association between femoral condyle ratio and graft rupture (graft ruptures n = 65, controls n = 352). A = adults, C&A = children/adolescents, NR = not reported, Follow-up = years.

Sex – sub group analysis age

Author & year	Population	Graft	Follow-up	Odds ratio	Lower limit	Upper limit
Borton et al. 2018 [67]	A	Hamstring	4.0	1.56	0.44	5.47
Lee et al. 2017 [68]	A	Mix	2.5	1.07	0.11	10.97
Pullen et al. 2016 [38]	A	NR	7.0	0.89	0.71	1.12
Salmon et al. 2018 [15]	A	Hamstring	19.7	0.98	0.40	2.41
Shelbourne et al. 1998 [69]	A	Patella	NR	0.85	0.30	2.45
Tulloch et al. 2019 [70]	A	LARS	7.8	0.96	0.30	3.07
				0.91	0.74	1.13
Bayomy et al. 2019 [84]	C&A	Hamstring	2.8	1.16	0.19	6.97
Bram et al. 2020 [62]	C&A	NR	4.3	0.55	0.30	1.00
Cruz et al. 2015 [85]	C&A	Mix	1.8	1.00	0.44	2.29
Ellis et al. 2012 [86]	C&A	Patella	4-11	0.42	0.09	2.03
Engelman et al. 2014 [87]	C&A	Hamstring	4.2	0.94	0.41	2.17
Fones et al. 2020 [59]	C&A	Mix	4.0	0.57	0.13	2.49
Ghosh et al. 2020 [63]	C&A	Hamstring	2.0	0.60	0.20	1.82
Graziano et al. 2017 [80]	C&A	Hamstring	NR	0.82	0.08	8.75
Heath et al. 2019 [64]	C&A	Hamstring	4.5	0.84	0.44	1.61
Ho et al. 2018 [89]	C&A	Mix	1.0	1.05	0.60	1.84
Larson et al. 2016 [90]	C&A	Mix	4.0	3.69	0.36	37.86
Morgan et al. 2016 [60]	C&A	Mix	16.5	0.56	0.27	1.15
Rauck et al. 2021 [91]	C&A	Patella	3.8	1.20	0.11	12.54
Salmon et al. [15]	C&A	Hamstring	19.7	0.38	0.10	1.50
Scmale et al. 2014 [92]	C&A	Mix	4.0	0.75	0.06	8.83
Wall et al. 2017 [93]	C&A	Hamstring	3.6	3.50	0.24	51.46
Webster et al. 2021 [94]	C&A	Mix	3.4	0.46	0.26	0.81
				0.71	0.57	0.89

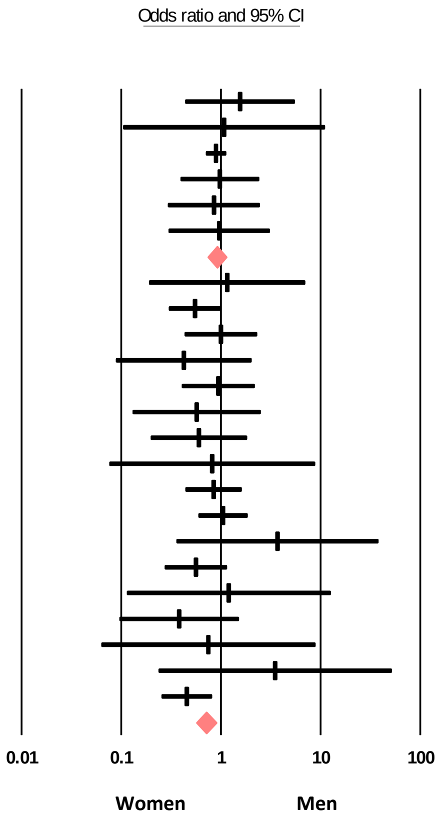

I² statistics: <0.001%

Figure 27. Meta-analysis on the association between sex and graft rupture in the sub groups of adults (A) and children/adolescents (C&A), respectively (graft ruptures n = 1018, controls n = 18 379). Mix = mix of different grafts, LARS = Ligament Advanced Reinforcement System, NR = not reported, Follow-up = years.

Age – sub group analysis children/adolescents

Author & year	Graft	Age	Follow-up	Odds ratio	Lower limit	Upper limit
Heath et al. 2018 [64]	Hamstring	>14<	4.5	0.54	0.26	1.12
Larson et al. 2016 [90]	Mix	>14<	4.0	1.63	0.23	11.46
Morgan et al. 2016 [60]	Mix	>14<	16.5	1.86	0.68	5.04
				1.03	0.40	2.60
Cruz et al. 2017 [85]	Mix	Continuous	1.8	1.70	0.55	5.25
Ho et al. 2018 [89]	Mix	Continuous	NR	0.71	0.43	1.19
Perkins et al. 2019 [79]	Hamstring	Continuous	2.0	0.61	0.35	1.04
Ranade et al. 2018 [105]	Hamstring	Continuous	4.0	1.49	0.47	4.71
				0.81	0.54	1.22

Statistics for each study; Odds ratio and 95% CI

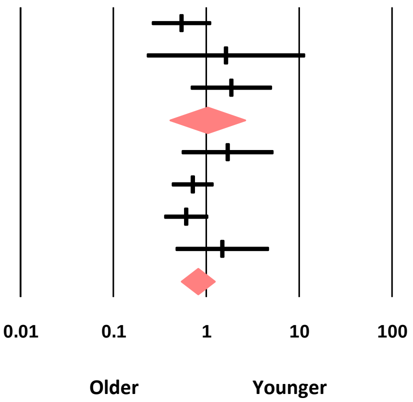

I² statistics
>14<: 53%
Continuous: <0.001%

Figure 28. Meta-analysis on the association between age and graft rupture in the sub group of children/adolescents (C&A) (>14<; graft ruptures n = 149, controls n = 519, continuous; graft ruptures n = 116, controls n = 913). Mix = mix of different grafts, NR = not reported, Follow-up = years.

BMI – sub group analysis children/adolescents

Author & year	Population	Graft	BMI	Follow-up	Odds ratio	Lower limit	Upper limit
Cruz et al. 2017 [85]	C&A	Mix	Continuous	1.8	1.70	0.55	5.26
Perkins et al. 2019 [75]	C&A	Hamstring	Continuous	2.0	0.62	0.36	1.07
					0.91	0.35	2.33

Statistics for each study; Odds ratio and 95% CI

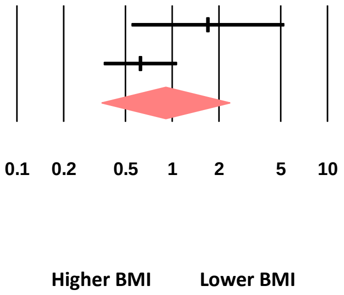

I² statistics: 59%

Figure 29. Meta-analysis on the association between BMI and graft rupture in the sub group of children/adolescents (C&A) (graft ruptures n = 62, controls n = 344). Mix = mix of different grafts, Follow-up = years.

Growth plate status

Author & year	Population	Graft	Follow-up	Odds ratio	Lower limit	Upper limit
				Statistics for each study		
Heath et al. 2019 [64]	C&A	Hamstring	4.5	0.67	0.35	1.27
Ho et al. 2018 [89]	C&A	Mix	1.0	1.62	0.86	3.08
				1.04	0.44	2.48

Odds ratio and 95% CI

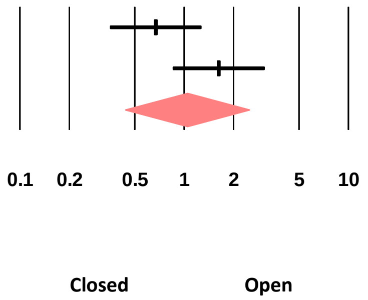

I² statistics: 72%

Figure 30. Meta-analysis on the association between growth plate status and graft rupture in the sub group of children/adolescents (C&A) (graft ruptures n = 113, controls n = 746). Mix = mix of different grafts, Follow-up = years.

References

1. Kaeding CC *et al*: **Change in Anterior Cruciate Ligament Graft Choice and Outcomes Over Time**. *Arthroscopy* 2017, **33**(11):2007-2014.
2. King E *et al*: **Factors Influencing Return to Play and Second Anterior Cruciate Ligament Injury Rates in Level 1 Athletes After Primary Anterior Cruciate Ligament Reconstruction: 2-Year Follow-up on 1432 Reconstructions at a Single Center**. *Am J Sports Med* 2020, **48**(4):812-824.
3. Beischer S *et al*: **Young Athletes Who Return to Sport Before 9 Months After Anterior Cruciate Ligament Reconstruction Have a Rate of New Injury 7 Times That of Those Who Delay Return**. *J Orthop Sports Phys Ther* 2020, **50**(2):83-90.
4. Filbay SR *et al*: **Delaying ACL reconstruction and treating with exercise therapy alone may alter prognostic factors for 5-year outcome: an exploratory analysis of the KANON trial**. *Br J Sports Med* 2017, **51**(22):1622-1629.
5. Fleming BC *et al*: **The effect of initial graft tension after anterior cruciate ligament reconstruction: a randomized clinical trial with 36-month follow-up**. *Am J Sports Med* 2013, **41**(1):25-34.
6. Henle P *et al*: **Patient and surgical characteristics that affect revision risk in dynamic intraligamentary stabilization of the anterior cruciate ligament**. *Knee Surg Sports Traumatol Arthrosc* 2018, **26**(4):1182-1189.

7. Henle P *et al*: **Surgeon experience with dynamic intraligamentary stabilization does not influence risk of failure**. *Knee Surg Sports Traumatol Arthrosc* 2018, **26**(10):2978-2985.
8. Kajetanek C *et al*: **Influence of the tibial slope on recurrent ACL ruptures**. *Journal de Traumatologie du Sport* 2017, **34**(1):37-43.
9. Krismer AM *et al*: **Factors influencing the success of anterior cruciate ligament repair with dynamic intraligamentary stabilisation**. *Knee Surg Sports Traumatol Arthrosc* 2017, **25**(12):3923-3928.
10. Tagesson S *et al*: **Greater fear of re-injury and increased tibial translation in patients who later sustain an ACL graft rupture or a contralateral ACL rupture: a pilot study**. *J Sports Sci* 2016, **34**(2):125-132.
11. Grassi A *et al*: **More Than a 2-Fold Risk of Contralateral Anterior Cruciate Ligament Injuries Compared With Ipsilateral Graft Failure 10 Years After Primary Reconstruction**. *Am J Sports Med* 2020:363546519893711.
12. Runer A *et al*: **Anterior Cruciate Ligament Reconstructions With Quadriceps Tendon Autograft Result in Lower Graft Rupture Rates but Similar Patient-Reported Outcomes as Compared With Hamstring Tendon Autograft: A Comparison of 875 Patients**. *Am J Sports Med* 2020, **48**(9):2195-2204.
13. Leys T *et al*: **Clinical results and risk factors for reinjury 15 years after anterior cruciate ligament reconstruction: a prospective study of hamstring and patellar tendon grafts**. *Am J Sports Med* 2012, **40**(3):595-605.
14. Rahardja R *et al*: **Rates of revision and surgeon-reported graft rupture following ACL reconstruction: early results from the New Zealand ACL Registry**. *Knee Surg Sports Traumatol Arthrosc* 2020, **28**(7):2194-2202.
15. Salmon LJ *et al*: **20-Year Outcomes of Anterior Cruciate Ligament Reconstruction With Hamstring Tendon Autograft: The Catastrophic Effect of Age and Posterior Tibial Slope**. *Am J Sports Med* 2018, **46**(3):531-543.
16. Shelbourne KD *et al*: **Incidence of subsequent injury to either knee within 5 years after anterior cruciate ligament reconstruction with patellar tendon autograft**. *Am J Sports Med* 2009, **37**(2):246-251.
17. Thompson S *et al*: **Twenty-year outcomes of a longitudinal prospective evaluation of isolated endoscopic anterior cruciate ligament reconstruction with patellar tendon autografts**. *Am J Sports Med* 2015, **43**(9):2164-2174.
18. Yabroudi MA *et al*: **Predictors of Revision Surgery After Primary Anterior Cruciate Ligament Reconstruction**. *Orthop J Sports Med* 2016, **4**(9):2325967116666039.
19. Gifstad T *et al*: **Lower risk of revision with patellar tendon autografts compared with hamstring autografts: a registry study based on 45,998 primary ACL reconstructions in Scandinavia**. *Am J Sports Med* 2014, **42**(10):2319-2328.
20. Lee DW *et al*: **Adolescents show a lower healing rate of anterolateral ligament injury and a higher rotational laxity than adults after anterior cruciate ligament reconstruction**. *Knee* 2021, **30**:113-124.
21. Magnussen RA *et al*: **Graft size and patient age are predictors of early revision after anterior cruciate ligament reconstruction with hamstring autograft**. *Arthroscopy* 2012, **28**(4):526-531.
22. Wasserstein D *et al*: **Risk factors for recurrent anterior cruciate ligament reconstruction: a population study in Ontario, Canada, with 5-year follow-up**. *Am J Sports Med* 2013, **41**(9):2099-2107.

23. Webster KE *et al*: **Younger patients are at increased risk for graft rupture and contralateral injury after anterior cruciate ligament reconstruction**. *Am J Sports Med* 2014, **42**(3):641-647.
24. Barrett AM *et al*: **Anterior cruciate ligament graft failure: a comparison of graft type based on age and Tegner activity level**. *Am J Sports Med* 2011, **39**(10):2194-2198.
25. Cooper DE *et al*: **Physiologic Preoperative Knee Hyperextension Is a Predictor of Failure in an Anterior Cruciate Ligament Revision Cohort: A Report From the MARS Group**. *Am J Sports Med* 2018, **46**(12):2836-2841.
26. Kamien PM *et al*: **Age, graft size, and Tegner activity level as predictors of failure in anterior cruciate ligament reconstruction with hamstring autograft**. *Am J Sports Med* 2013, **41**(8):1808-1812.
27. Mardani-Kivi M *et al*: **Contralateral and siblings' knees are at higher risk of ACL tear for patients with a positive history of ACL tear**. *Knee Surg Sports Traumatol Arthrosc* 2019.
28. Annear PT *et al*: **No clinical difference in 10-year outcomes between standard and minimal graft debridement techniques in patients undergoing anterior cruciate ligament reconstruction using autologous hamstrings: a randomized controlled trial**. *Knee Surg Sports Traumatol Arthrosc* 2019, **27**(2):516-523.
29. Benner RW *et al*: **The Degree of Knee Extension Does Not Affect Postoperative Stability or Subsequent Graft Tear Rate After Anterior Cruciate Ligament Reconstruction With Patellar Tendon Autograft**. *Am J Sports Med* 2016, **44**(4):844-849.
30. Gupta R *et al*: **Predictors for Anterior Cruciate Ligament (ACL) Re-injury after Successful Primary ACL Reconstruction (ACLR)**. *Malays Orthop J* 2020, **14**(3):50-56.
31. Ifran NN *et al*: **Tear Rates of the Ipsilateral ACL Graft and the Contralateral Native ACL Are Similar following ACL Reconstruction**. *J Knee Surg* 2020.
32. Kim SH *et al*: **Predictive factors for failure of anterior cruciate ligament reconstruction via the trans-tibial technique**. *Arch Orthop Trauma Surg* 2020, **140**(10):1445-1457.
33. Kyritsis P *et al*: **Likelihood of ACL graft rupture: not meeting six clinical discharge criteria before return to sport is associated with a four times greater risk of rupture**. *Br J Sports Med* 2016, **50**(15):946-951.
34. Laboute E *et al*: **Analysis of return to competition and repeat rupture for 298 anterior cruciate ligament reconstructions with patellar or hamstring tendon autograft in sportspeople**. *Ann Phys Rehabil Med* 2010, **53**(10):598-614.
35. Maletis GB *et al*: **Risk factors associated with revision and contralateral anterior cruciate ligament reconstructions in the Kaiser Permanente ACLR registry**. *Am J Sports Med* 2015, **43**(3):641-647.
36. Okoroha KR *et al*: **Length of Time Between Anterior Cruciate Ligament Reconstruction and Return to Sport Does Not Predict Need for Revision Surgery in National Football League Players**. *Arthroscopy* 2019, **35**(1):158-162.
37. Poston GR *et al*: **Reduced 2-D Frontal Plane Motion During Single-Limb Landing Is Associated With Risk of Future Anterior Cruciate Ligament Graft Rupture After Anterior Cruciate Ligament Reconstruction and Return to Sport: A Pilot Study**. *J Orthop Sports Phys Ther* 2021, **51**(2):82-87.
38. Pullen WM *et al*: **Predictors of Revision Surgery After Anterior Cruciate Ligament Reconstruction**. *Am J Sports Med* 2016, **44**(12):3140-3145.
39. Riff AJ *et al*: **Thirty-Year Experience With ACL Reconstruction Using Patellar Tendon: A Critical Evaluation of Revision and Reoperation**. *Orthop J Sports Med* 2017, **5**(8):2325967117724345.
40. Singhal MC *et al*: **Failure of primary anterior cruciate ligament surgery using anterior tibialis allograft**. *Arthroscopy* 2007, **23**(5):469-475.

41. van Eck CF *et al*: **Prospective analysis of failure rate and predictors of failure after anatomic anterior cruciate ligament reconstruction with allograft**. *Am J Sports Med* 2012, **40**(4):800-807.
42. Christensen JJ *et al*: **Lateral Tibial Posterior Slope Is Increased in Patients With Early Graft Failure After Anterior Cruciate Ligament Reconstruction**. *Am J Sports Med* 2015, **43**(10):2510-2514.
43. Digiacomo JE *et al*: **Examination of Knee Morphology After Secondary Ipsilateral ACL Injury Compared With Those That Have Not Been Reinjured: A Preliminary Study**. *J Sport Rehabil* 2018, **27**(1):73-82.
44. Grassi A *et al*: **Patients With Failed Anterior Cruciate Ligament Reconstruction Have an Increased Posterior Lateral Tibial Plateau Slope: A Case-Controlled Study**. *Arthroscopy* 2019, **35**(4):1172-1182.
45. Jaecker V *et al*: **Increased medial and lateral tibial posterior slopes are independent risk factors for graft failure following ACL reconstruction**. *Arch Orthop Trauma Surg* 2018, **138**(10):1423-1431.
46. Mitchell BC *et al*: **Predictive Value of the Magnetic Resonance Imaging-Based Coronal Lateral Collateral Ligament Sign on Adolescent Anterior Cruciate Ligament Reconstruction Graft Failure**. *Am J Sports Med* 2021, **49**(4):935-940.
47. Sauer S *et al*: **The influence of tibial slope on anterior cruciate ligament graft failure risk is dependent on graft positioning**. *J Orthop Surg (Hong Kong)* 2019, **27**(1):2309499019834674.
48. Webb JM *et al*: **Posterior tibial slope and further anterior cruciate ligament injuries in the anterior cruciate ligament-reconstructed patient**. *Am J Sports Med* 2013, **41**(12):2800-2804.
49. King E *et al*: **Biomechanical but Not Strength or Performance Measures Differentiate Male Athletes Who Experience ACL Reinjury on Return to Level 1 Sports**. *Am J Sports Med* 2021, **49**(4):918-927.
50. McPherson AL *et al*: **Psychological Readiness to Return to Sport Is Associated With Second Anterior Cruciate Ligament Injuries**. *Am J Sports Med* 2019, **47**(4):857-862.
51. Fältstrom A *et al*: **Predictors for additional anterior cruciate ligament reconstruction: data from the Swedish national ACL register**. *Knee Surg Sports Traumatol Arthrosc* 2016, **24**(3):885-894.
52. Allen MM *et al*: **Are Female Soccer Players at an Increased Risk of Second Anterior Cruciate Ligament Injury Compared With Their Athletic Peers?** *Am J Sports Med* 2016, **44**(10):2492-2498.
53. Bodkin SG *et al*: **Predicting ACL Reinjury from Return to Activity Assessments at 6-months Post-Surgery: A Prospective Cohort Study**. *J Athl Train* 2021.
54. Bourke HE *et al*: **Survival of the anterior cruciate ligament graft and the contralateral ACL at a minimum of 15 years**. *Am J Sports Med* 2012, **40**(9):1985-1992.
55. Salmon L *et al*: **Incidence and risk factors for graft rupture and contralateral rupture after anterior cruciate ligament reconstruction**. *Arthroscopy* 2005, **21**(8):948-957.
56. Sandon A *et al*: **High Risk of Further Anterior Cruciate Ligament Injury in a 10-Year Follow-up Study of Anterior Cruciate Ligament-Reconstructed Soccer Players in the Swedish National Knee Ligament Registry**. *Arthroscopy* 2020, **36**(1):189-195.
57. Welling W *et al*: **Passing return to sports tests after ACL reconstruction is associated with greater likelihood for return to sport but fail to identify second injury risk**. *Knee* 2020, **27**(3):949-957.
58. Britt E *et al*: **The Challenges of Treating Female Soccer Players With ACL Injuries: Hamstring Versus Bone-Patellar Tendon-Bone Autograft**. *Orthop J Sports Med* 2020, **8**(11):2325967120964884.

59. Fones L *et al*: **Patient-Reported Outcomes, Return-to-Sport Status, and Reinjury Rates After Anterior Cruciate Ligament Reconstruction in Adolescent Athletes: Minimum 2-Year Follow-up**. *Orthop J Sports Med* 2020, **8**(11):2325967120964471.
60. Morgan MD *et al*: **Fifteen-Year Survival of Endoscopic Anterior Cruciate Ligament Reconstruction in Patients Aged 18 Years and Younger**. *Am J Sports Med* 2016, **44**(2):384-392.
61. Goshima K *et al*: **Familial predisposition to anterior cruciate ligament injury**. *Asia-Pacific Journal of Sports Medicine, Arthroscopy, Rehabilitation and Technology* 2014, **1**(2):62-66.
62. Bram JT *et al*: **Do Pediatric Patients With Anterior Cruciate Ligament Tears Have a Higher Rate of Familial Anterior Cruciate Ligament Injury?** *Orthop J Sports Med* 2020, **8**(10):2325967120959665.
63. Ghosh K *et al*: **Transphyseal anterior cruciate ligament reconstruction using living parental donor hamstring graft: excellent clinical results at 2 years in a cohort of 100 patients**. *Knee Surg Sports Traumatol Arthrosc* 2020, **28**(8):2511-2518.
64. Heath EL *et al*: **5-Year Survival of Pediatric Anterior Cruciate Ligament Reconstruction With Living Donor Hamstring Tendon Grafts**. *Am J Sports Med* 2019, **47**(1):41-51.
65. Everhart JS *et al*: **Cartilage damage at the time of anterior cruciate ligament reconstruction is associated with weaker quadriceps function and lower risk of future ACL injury**. *Knee Surg Sports Traumatol Arthrosc* 2020, **28**(2):576-583.
66. Lord L *et al*: **One sixth of primary anterior cruciate ligament reconstructions may undergo reoperation due to complications or new injuries within 2 years**. *Knee Surg Sports Traumatol Arthrosc* 2020, **28**(8):2478-2485.
67. Borton ZM *et al*: **Mid-bundle positioning of the femoral socket increases graft rupture in anatomic single bundle anterior cruciate ligament reconstruction**. *Knee* 2018, **25**(6):1122-1128.
68. Lee BH *et al*: **Comparison of anterior cruciate ligament volume after anatomic double-bundle anterior cruciate ligament reconstruction**. *Knee* 2017, **24**(3):580-587.
69. Shelbourne KD *et al*: **The relationship between intercondylar notch width of the femur and the incidence of anterior cruciate ligament tears. A prospective study**. *Am J Sports Med* 1998, **26**(3):402-408.
70. Tulloch SJ *et al*: **Primary ACL reconstruction using the LARS device is associated with a high failure rate at minimum of 6-year follow-up**. *Knee Surg Sports Traumatol Arthrosc* 2019, **27**(11):3626-3632.
71. Csapo R *et al*: **Contralateral ACL tears strongly contribute to high rates of secondary ACL injuries in professional ski racers**. *Knee Surg Sports Traumatol Arthrosc* 2021, **29**(6):1805-1812.
72. Larson CM *et al*: **Generalized Hypermobility, Knee Hyperextension, and Outcomes After Anterior Cruciate Ligament Reconstruction: Prospective, Case-Control Study With Mean 6 Years Follow-up**. *Arthroscopy* 2017, **33**(10):1852-1858.
73. Mohtadi N *et al*: **Reruptures, Reinjuries, and Revisions at a Minimum 2-Year Follow-up: A Randomized Clinical Trial Comparing 3 Graft Types for ACL Reconstruction**. *Clin J Sport Med* 2016, **26**(2):96-107.
74. Paterno MV *et al*: **Incidence of contralateral and ipsilateral anterior cruciate ligament (ACL) injury after primary ACL reconstruction and return to sport**. *Clin J Sport Med* 2012, **22**(2):116-121.
75. Perkins CA *et al*: **Allograft Augmentation of Hamstring Anterior Cruciate Ligament Autografts Is Associated With Increased Graft Failure in Children and Adolescents**. *Am J Sports Med* 2019, **47**(7):1576-1582.

76. Pinczewski LA *et al*: **A 10-year comparison of anterior cruciate ligament reconstructions with hamstring tendon and patellar tendon autograft: a controlled, prospective trial**. *Am J Sports Med* 2007, **35**(4):564-574.
77. Rosenstiel N *et al*: **Combined Anterior Cruciate and Anterolateral Ligament Reconstruction in the Professional Athlete: Clinical Outcomes From the Scientific Anterior Cruciate Ligament Network International Study Group in a Series of 70 Patients With a Minimum Follow-Up of 2 Years**. *Arthroscopy* 2019, **35**(3):885-892.
78. Salmon LJ *et al*: **Long-term outcome of endoscopic anterior cruciate ligament reconstruction with patellar tendon autograft: minimum 13-year review**. *Am J Sports Med* 2006, **34**(5):721-732.
79. Sanders TL *et al*: **Long-term rate of graft failure after ACL reconstruction: a geographic population cohort analysis**. *Knee Surg Sports Traumatol Arthrosc* 2017, **25**(1):222-228.
80. Schlumberger M *et al*: **Traumatic graft rupture after primary and revision anterior cruciate ligament reconstruction: retrospective analysis of incidence and risk factors in 2915 cases**. *Knee Surg Sports Traumatol Arthrosc* 2017, **25**(5):1535-1541.
81. Sousa PL *et al*: **Return to sport: Does excellent 6-month strength and function following ACL reconstruction predict midterm outcomes?** *Knee Surg Sports Traumatol Arthrosc* 2017, **25**(5):1356-1363.
82. Webster KE *et al*: **Preservation of the Tibial Stump During Anterior Cruciate Ligament Reconstruction Surgery Did Not Increase the Rate of Surgery for Symptomatic Cyclops Lesions**. *Orthop J Sports Med* 2021, **9**(4):2325967121992517.
83. Wright RW *et al*: **Risk of tearing the intact anterior cruciate ligament in the contralateral knee and rupturing the anterior cruciate ligament graft during the first 2 years after anterior cruciate ligament reconstruction: a prospective MOON cohort study**. *Am J Sports Med* 2007, **35**(7):1131-1134.
84. Bayomy AF *et al*: **The Impact of Transphyseal Anterior Cruciate Ligament Reconstruction on Lower Extremity Growth and Alignment**. *Arthroscopy* 2019, **35**(3):940-949.
85. Cruz AI, Jr. *et al*: **All-Epiphyseal ACL Reconstruction in Children: Review of Safety and Early Complications**. *J Pediatr Orthop* 2017, **37**(3):204-209.
86. Ellis HB *et al*: **Outcomes and revision rate after bone-patellar tendon-bone allograft versus autograft anterior cruciate ligament reconstruction in patients aged 18 years or younger with closed physes**. *Arthroscopy* 2012, **28**(12):1819-1825.
87. Engelman GH *et al*: **Comparison of allograft versus autograft anterior cruciate ligament reconstruction graft survival in an active adolescent cohort**. *Am J Sports Med* 2014, **42**(10):2311-2318.
88. Graziano J *et al*: **Return to Sport for Skeletally Immature Athletes After ACL Reconstruction: Preventing a Second Injury Using a Quality of Movement Assessment and Quantitative Measures to Address Modifiable Risk Factors**. *Orthop J Sports Med* 2017, **5**(4):2325967117700599.
89. Ho B *et al*: **Risk Factors for Early ACL Reconstruction Failure in Pediatric and Adolescent Patients: A Review of 561 Cases**. *J Pediatr Orthop* 2018, **38**(7):388-392.
90. Larson CM *et al*: **Allograft and Autograft Transphyseal Anterior Cruciate Ligament Reconstruction in Skeletally Immature Patients: Outcomes and Complications**. *Arthroscopy* 2016, **32**(5):860-867.
91. Rauck RC *et al*: **Return to Sport After Bone-Patellar Tendon-Bone Autograft ACL Reconstruction in High School-Aged Athletes**. *Orthop J Sports Med* 2021, **9**(6):23259671211011510.

92. Schmale GA *et al*: **High satisfaction yet decreased activity 4 years after transphyseal ACL reconstruction**. *Clin Orthop Relat Res* 2014, **472**(7):2168-2174.
93. Wallace IJ *et al*: **Knee osteoarthritis has doubled in prevalence since the mid-20th century**. *Proc Natl Acad Sci U S A* 2017, **114**(35):9332-9336.
94. Webster KE *et al*: **Second ACL Injury Rates in Younger Athletes Who Were Advised to Delay Return to Sport Until 12 Months After ACL Reconstruction**. *Orthop J Sports Med* 2021, **9**(2):2325967120985636.
95. Grassi A *et al*: **Higher risk of contralateral anterior cruciate ligament (ACL) injury within 2 years after ACL reconstruction in under-18-year-old patients with steep tibial plateau slope**. *Knee Surg Sports Traumatol Arthrosc* 2021, **29**(6):1690-1700.
96. Lee CC *et al*: **Does Posterior Tibial Slope Affect Graft Rupture Following Anterior Cruciate Ligament Reconstruction?** *Arthroscopy* 2018, **34**(7):2152-2155.
97. Su AW *et al*: **Medial Tibial Slope Determined by Plain Radiography Is Not Associated with Primary or Recurrent Anterior Cruciate Ligament Tears**. *J Knee Surg* 2020, **33**(1):22-28.
98. Akhtar MA *et al*: **Generalised ligamentous laxity and revision ACL surgery: Is there a relation?** *Knee* 2016, **23**(6):1148-1153.
99. Vincent YP-h *et al*: **Role of Physiotherapy in Preventing Failure of Primary Anterior Cruciate Ligament Reconstruction**. *Journal of Orthopaedics, Trauma and Rehabilitation* 2017, **22**(1):6-12.
100. Fältström A *et al*: **Poor Validity of Functional Performance Tests to Predict Knee Injury in Female Soccer Players With or Without Anterior Cruciate Ligament Reconstruction**. *Am J Sports Med* 2021, **49**(6):1441-1450.
101. Webster KE *et al*: **Clinical Tests Can Be Used to Screen for Second Anterior Cruciate Ligament Injury in Younger Patients Who Return to Sport**. *Orthop J Sports Med* 2019, **7**(8):2325967119863003.
102. Krosshaug T *et al*: **The Vertical Drop Jump Is a Poor Screening Test for ACL Injuries in Female Elite Soccer and Handball Players: A Prospective Cohort Study of 710 Athletes**. *Am J Sports Med* 2016, **44**(4):874-883.
103. Akada T *et al*: **Partial meniscectomy adversely affects return-to-sport outcome after anatomical double-bundle anterior cruciate ligament reconstruction**. *Knee Surg Sports Traumatol Arthrosc* 2019, **27**(3):912-920.
104. Pfeiffer TR *et al*: **An Increased Lateral Femoral Condyle Ratio Is a Risk Factor for Anterior Cruciate Ligament Injury**. *J Bone Joint Surg Am* 2018, **100**(10):857-864.
105. Ranade SC *et al*: **Validated Pediatric Functional Outcomes of All-epiphyseal ACL Reconstructions: Does Reinjury Affect Outcomes?** *J Pediatr Orthop* 2020, **40**(4):157-161.
