# Supplementary material for: Return to Sports: A Risky Business? A Systematic Review with Meta-Analysis of Risk Factors for Graft Rupture Following ACL Reconstruction
Source: Sports Med. 2022 Aug 24;53(1):91–110. doi: 10.1007/s40279-022-01747-3 (PMC9807539; doi:10.1007/s40279-022-01747-3)
Supplement: Supplementary file 6 — Supplementary file6 (PDF 611 KB) [file 40279_2022_1747_MOESM6_ESM.pdf]

Return to sports - a risky business? A systematic review with meta-analysis of risk factors for graft rupture following ACL reconstruction, Sports Medicine, Anna Cronström; anna.cronstrom@umu.se, Eva Tengman, Charlotte Häger, Umeå University

Online resource 6. Risk of bias for all papers included in the meta-analyses and publication bias for sex, family history, return to sport and concomitant meniscal tear as risk factors for graft rupture

Table 1. Risk of bias assessment using the QUIPS tool

Article	Study participation	Study attrition	Prognostic factor measurement	Outcome measurement	Study confounding	Statistical analysis	RoB
Ahktar et al. 2016	High	Low	Low	Low	High	Low	**High**
Akada et al. 2019	Low	Low	Low	Low	High	Low	**High**
Allen et al. 2016	Moderate	Low	Low	Low	Low	Low	**Low**
Annear et al. 2019	Moderate	Low	Low	Moderate	Moderate	Low	**High**
Barret et al. 2011	Low	Moderate	Low	Low	High	Low	**High**
Bayomy et al. 2019	Moderate	Low	Low	Low	Low	Low	**Low**
Beischer et al. 2020	Moderate	Low	Low	Low	Moderate	Low	**Moderate**
Benner et al. 2016	Moderate	Low	Low	Low	Moderate	Low	**Moderate**
Bodkin et al. 2021	Low	Low	Low	Low	Low	Low	**Low**
Borton et al. 2018	Moderate	Low	Low	Moderate	Moderate	Low	**High**
Bourke et al. 2012	Moderate	Low	Low	Moderate	Low	Low	**Moderate**
Bram et al. 2020	Low	High	Low	Moderate	Low	Low	**High**
Britt et al. 2020	Moderate	Low	Low	Low	Moderate	Low	**Moderate**
Christensen et al. 2015	Moderate	Low	Low	Low	Low	Low	**Low**
Cooper et al. 2018	High	Low	High	Low	Low	Low	**High**
Cruz et al. 2017	Moderate	Low	Low	Low	Low	Low	**Low**
Csapo et al. 2021	Low	Low	Low	Low	Moderate	Low	**Low**
Defranesco et al. 2020	Moderate	Low	Low	Moderate	Moderate	Low	**High**
Della Villa et al. 2021	Low	Low	Low	Low	Low	Low	**Low**
Digiacomo et al. 2018	Moderate	Low	Low	Low	Moderate	Low	**Moderate**
Ellis et al. 2012	Low	Low	Low	Low	Low	Low	**Low**
Engelman et al. 2014	Moderate	High	Low	Low	Low	Low	**High**
Everhart et al. 2020	Moderate	Low	Low	Low	Low	Low	**Low**
Filbay et al. 2017	Moderate	Low	Low	Low	Low	Low	**Low**
Fleming et al. 2013	Moderate	Low	Low	Low	Moderate	Low	**Moderate**
Fones et al. 2020	Low	Moderate	Low	Moderate	Low	Low	**Moderate**
Fältström et al. 2016	Low	Moderate	Low	Low	Low	Low	**Low**
Fältström et al. 2021	Moderate	Low	Low	Low	Moderate	Low	**Moderate**
Gagliardi et al. 2019	Moderate	Low	Low	Low	Low	Low	**Low**

Return to sports - a risky business? A systematic review with meta-analysis of risk factors for graft rupture following ACL reconstruction, Sports Medicine, Anna Cronström; anna.cronstrom@umu.se, Eva Tengman, Charlotte Häger, Umeå University

Gans et al. 2018	High	Low	Low	Moderate	Moderate	Low	**High**
Ghosh et al. 2020	Moderate	Low	Low	Moderate	High	Low	**High**
Gifstad et al. 2014	Low	Low	Low	Low	Low	Low	**Low**
Goncalvez et.al 2017	Moderate	Low	Low	Low	Low	Low	**Low**
Goshima et a. 2014	Moderate	Low	Low	Moderate	Moderate	Low	**High**
Grassi et al. 2020	Low	Low	Low	Low	Low	Low	**Low**
Grassi et al. 2021	Moderate	Low	Low	Low	Low	Low	**Low**
Grassi et al. 2019	Moderate	Low	Low	Low	Low	Low	**Low**
Graziano et al. 2017	Moderate	Low	Low	Moderate	High	Low	**High**
Gupta et al. 2019	Moderate	Low	Low	Moderate	High	Low	**High**
Heath et al. 2019	Low	Low	Low	Low	Low	Low	**Low**
Henle et al. 2018[1]	Low	Low	Low	Low	Low	Low	**Low**
Henle et al. 2018[2]	Moderate	Low	Low	Low	Low	Low	**Low**
Ho et al. 2018	Moderate	Low	Low	Low	Low	Low	**Low**
Ifran et al. 2020	Moderate	Low	Low	Low	Low	Low	**Low**
Jaecker et al. 2018	Moderate	Low	Low	Low	Low	Low	**Low**
Kaeding et al. 2017	Low	Low	Low	Low	Low	Low	**Low**
Kajetanek et al. 2017	Low	Low	Moderate	Low	Moderate	Low	**Moderate**
Kamien et al. 2013	Moderate	Low	Low	Low	High	Low	**High**
Kim et al. 2020	Low	Low	Low	Low	Low	Low	**Low**
King et al. 2020	Low	Low	Low	Low	Low	Low	**Low**
King et al. 2021	Moderate	Low	Moderate	Moderate	Low	Low	**High**
Kinsella et.al 2020	High	Low	Low	Low	Low	Low	**High**
Krismer et.al 2017	Moderate	Low	Moderate	Low	Low	Low	**Moderate**
Krosshaug et al. 2016	Low	Low	Low	Low	Low	Low	**Low**
Kyritsis et al. 2016	Low	Low	Low	Moderate	Low	Low	**Low**
Laboute et al. 2010	Low	Moderate	Low	Moderate	Moderate	Low	**High**
Lai et.al 2018	Moderate	Moderate	Low	Low	High	Low	**High**
Larson et al 2016	Low	Low	Low	Moderate	Low	Low	Low
Larson et al. 2017	High	Low	Moderate	Low	High	Low	**High**
Lee et al. 2018	High	Low	Low	Low	Low	Low	**High**
Lee et al. 2017	Moderate	Low	Low	Moderate	Moderate	Low	**High**
Lee 2021	Low	Low	Low	Low	Low	Low	**Low**
Levins et al. 2016	Moderate	High	Low	Low	Low	Low	High
Leys et al. 2012	Moderate	Low	Low	Moderate	Moderate	Low	**High**
Lord et al. 2020	Moderate	Low	Low	Low	Low	Low	**Low**
Magnussen et al. 2012	Low	High	Low	Low	Moderate	Low	**High**
Magnusson et al. 2018	Low	Low	Moderate	Low	Low	Low	**Low**
Maletis et al. 2015	Moderate	Low	Low	Low	Low	Low	**Low**

Return to sports - a risky business? A systematic review with meta-analysis of risk factors for graft rupture following ACL reconstruction, Sports Medicine, Anna Cronström; anna.cronstrom@umu.se, Eva Tengman, Charlotte Häger, Umeå University

Mardani-Kivi et al. 2019	Low	Moderate	Low	Moderate	High	Low	**High**
McPherson et al. 2019	Low	Low	Low	Low	Moderate	Low	**Low**
Mitchell et al. 2021	Moderate	Low	Low	Low	Low	Low	**Low**
Mohtadi et al. 2016	Low	Low	Low	Low	Low	Low	**Low**
Morgan et al. 2016	Low	Low	Low	Moderate	Low	Low	**Low**
Okoroha et al. 2019	Low	Low	Low	Low	Low	Low	**Low**
Patel et al. 2019	Low	Low	Low	Low	Low	Low	**Low**
Paterno et al. 2012	Moderate	Low	Low	Low	Moderate	Low	**Moderate**
Paterno et al. 2018	High	Low	Low	Moderate	High	Low	**High**
Perkins et al. 2019	Moderate	Low	Low	Low	Low	Low	**Low**
Pfeiffer et al. 2018	Moderate	Low	Low	Low	Moderate	Low	**Moderate**
Pinczewski et al. 2007	Moderate	Low	Moderate	Moderate	Moderate	Low	**High**
Poston et al. 2020	Moderate	Low	Low	Low	Low	Low	**Low**
Pullen et al. 2016	Low	Low	Low	Low	Low	Low	**Low**
Rahardja et al. 2020	Moderate	Low	Low	Low	Low	Low	**Low**
Ranade et al. 2018	High	Low	Low	Moderate	Moderate	Low	**High**
Rauck et al. 2021	Low	High	Moderate	Low	Low	Low	**High**
Riff et al. 2017	Moderate	Low	Low	Low	High	Low	**High**
Rosenstiel et al. 2019	Low	Low	Low	Low	Low	Low	**Low**
Runer et al. 2020	Low	Low	Low	Moderate	Low	Low	**Low**
Salmon et al. 2005	Low	Low	Low	Low	Low	Low	**Low**
Salmon et al. 2006	Low	Low	Low	Low	Low	Low	**Low**
Salmon et al. 2018	Moderate	Low	Low	Low	Low	Low	**Low**
Sanders et al. 2017	Moderate	Low	Low	Low	Low	Low	**Low**
Sandon et al. 2020	Low	High	Low	Moderate	High	Low	**High**
Sauer et al. 2019	Moderate	Low	Low	Low	Low	Low	**Low**
Schlumberger et al. 2017	Moderate	Low	Low	Low	Moderate	Low	**Moderate**
Schmale et al. 2014	Low	High	Low	Low	High	Low	**High**
Shelbourne et al. 2009	Low	Moderate	Low	Moderate	High	Low	**High**
Shelbourne et al. 1998	Low	Low	Low	Moderate	Moderate	Low	**Moderate**
Singhal et al. 2007	Moderate	High	Low	Low	High	Moderate	**High**
Sonnery-Cottet et al. 2017	Low	Low	Low	Low	Low	Low	**Low**
Sousa et al. 2017	Low	Low	Low	Low	Low	Low	**Low**
Su et al. 2020	High	Low	Low	Low	Low	Low	**High**
Tagesson et al. 2016	Moderate	Low	Low	Low	Moderate	Low	**Moderate**
Thompson et al. 2015	Moderate	Low	Low	Low	Low	Low	**Low**

Return to sports - a risky business? A systematic review with meta-analysis of risk factors for graft rupture following ACL reconstruction, Sports Medicine, Anna Cronström; anna.cronstrom@umu.se, Eva Tengman, Charlotte Häger, Umeå University

Tulloch et al. 2019	Low	Low	Low	Low	Low	Low	**Low**
Van Eck et al. 2012	Moderate	Low	Low	Low	Low	Low	**Low**
Vincent et al. 2017	Moderate	Low	Low	Low	Low	Low	**Low**
Wall et al. 2017	Moderate	Moderate	Low	Low	Low	Low	**Moderate**
Wasserstein et al. 2013	Moderate	Low	Low	Low	Low	Low	**Low**
Webb et al. 2013	High	Low	Low	Low	Low	Low	**High**
Welling et al. 2020	Moderate	Low	Low	Moderate	Moderate	Low	**High**
Webster et al. 2014	Moderate	Low	Low	Low	Moderate	Low	**Moderate**
Webster et al. 2019	Moderate	Low	Moderate	Moderate	Moderate	Low	**High**
Webster et al. 2021	Low	Low	Low	Low	High	Low	**High**
Webster et a. 2021	Low	Low	Low	Moderate	Low	Low	**Low**
Wright et al. 2007	Moderate	Low	Low	Low	High	Low	**High**
Yabroudi et al. 2016	Moderate	High	Low	Low	Low	Low	**High**

RoB = risk of bias, Low RoB = all domains are rated as low or at most one rated as moderate, moderate Rob = 4 domains are rated as low and two domains are rated as moderate RoB, high RoB = at least 3 domains are rated as moderate or at least 1 domain rated as high RoB.

Return to sports - a risky business? A systematic review with meta-analysis of risk factors for graft rupture following ACL reconstruction, Sports Medicine, Anna Cronström; anna.cronstrom@umu.se, Eva Tengman, Charlotte Häger, Umeå University

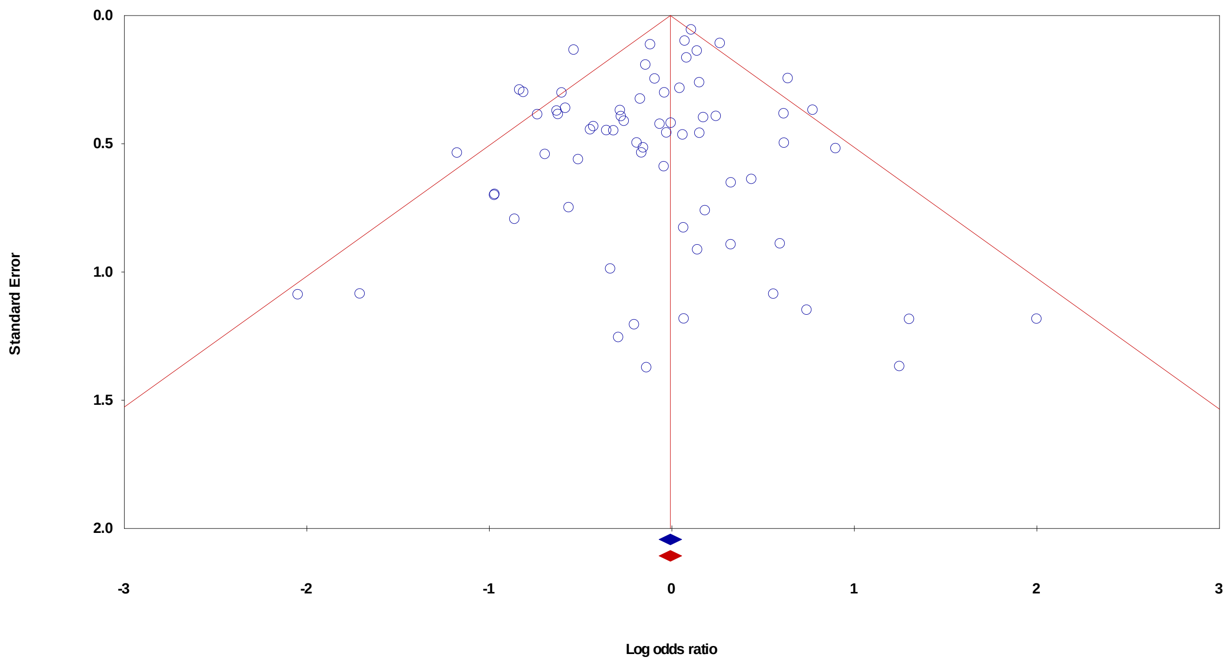

Fig 1. Funnel plot with trim and fill imputations for sex difference as a risk factor for sustaining a graft rupture

Return to sports - a risky business? A systematic review with meta-analysis of risk factors for graft rupture following ACL reconstruction, Sports Medicine, Anna Cronström; anna.cronstrom@umu.se, Eva Tengman, Charlotte Häger, Umeå University

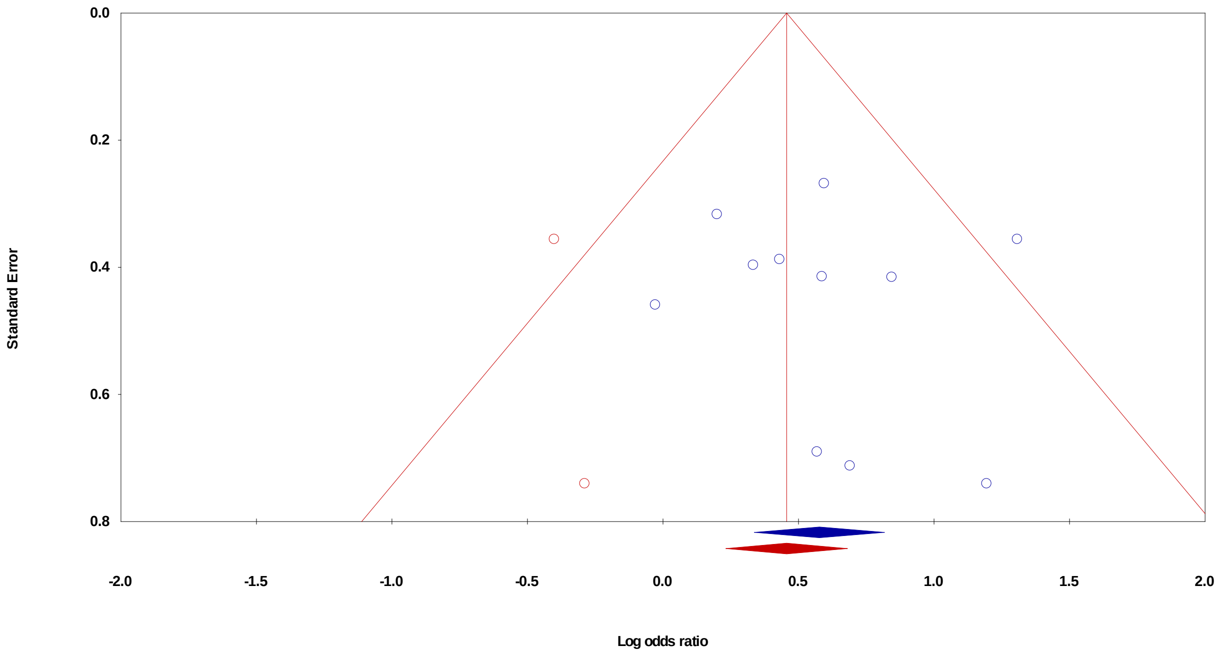

Fig 2. Funnel plot with trim and fill imputations for family history as a risk factor for sustaining a graft rupture

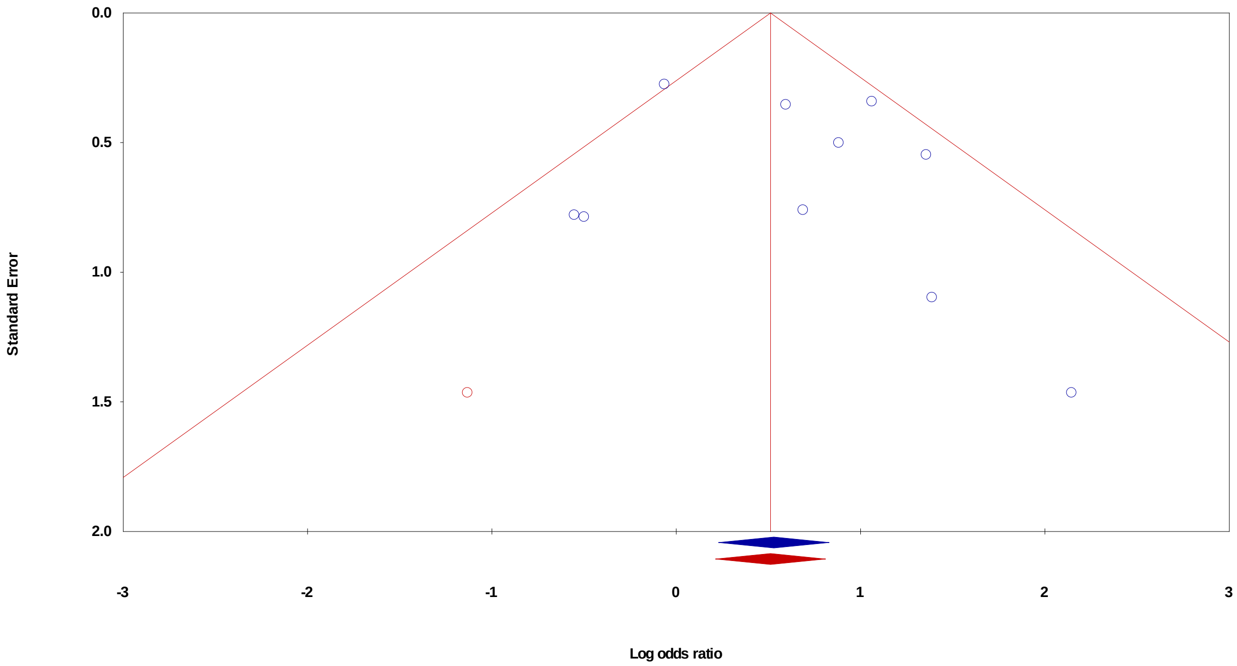

Fig 3. Funnel plot with trim and fill imputations for return to a high activity level as a risk factor for sustaining a graft rupture

Return to sports - a risky business? A systematic review with meta-analysis of risk factors for graft rupture following ACL reconstruction, Sports Medicine, Anna Cronström; anna.cronstrom@umu.se, Eva Tengman, Charlotte Häger, Umeå University

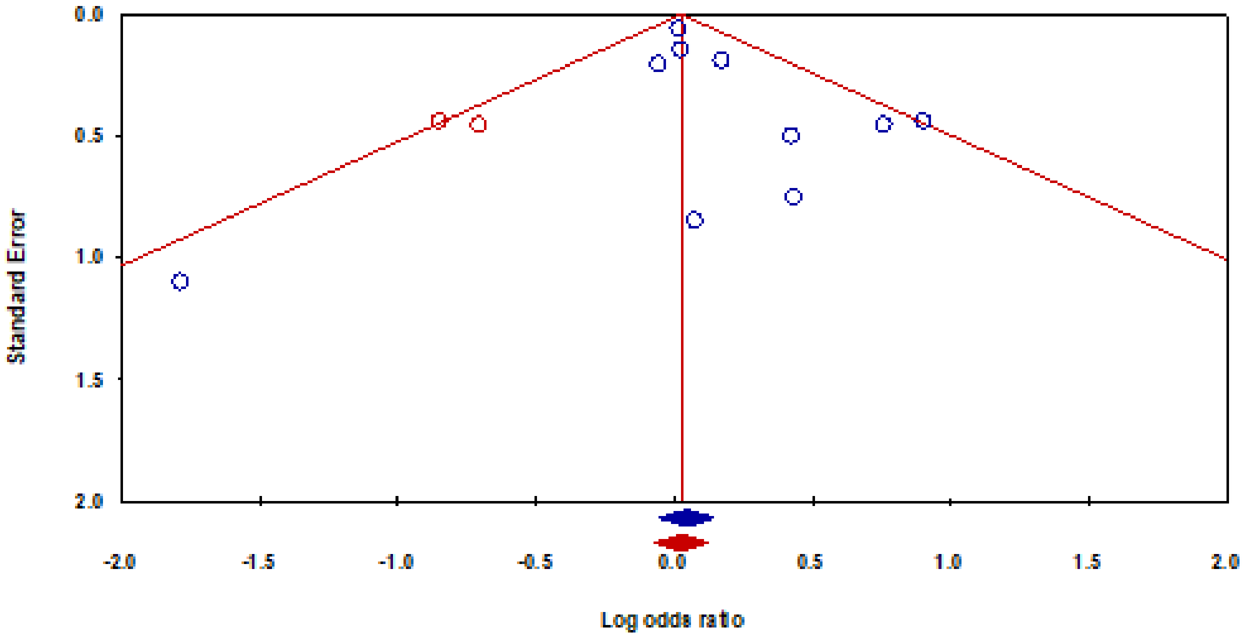

Fig 4. Funnel plot with trim and fill imputations for concomitant meniscal tear as a risk factor for sustaining a graft rupture
