# Supplementary material for: Return to Sports: A Risky Business? A Systematic Review with Meta-Analysis of Risk Factors for Graft Rupture Following ACL Reconstruction
Source: Sports Med. 2022 Aug 24;53(1):91–110. doi: 10.1007/s40279-022-01747-3 (PMC9807539; doi:10.1007/s40279-022-01747-3)
Supplement: Supplementary file 7 — Supplementary file7 (PDF 458 KB) [file 40279_2022_1747_MOESM7_ESM.pdf]

Table 1. Sensitivity analysis of meta-analytic findings with the exclusion of studies with high risk of bias

	**All studies**		**High risk of bias studies excluded**		**Findings with exclusion of studies**
**Risk factor**	Studies (n)	OR (95% CI)	Studies (n)	OR (95% CI)	
Age ≥ 18 vs. < 18	7	3.03 (1.74 – 5.27)	4	2.15 (1.43 – 3.24)	No changes
Age ≥ 20 vs. < 20	5	3.53 (2.29 – 5.45)	4	3.21 (2.14 – 4.82)	No changes
Age ≥ 25 vs. < 25	5	2.86 (1.97 – 4.16)	2	2.76 (1.54 – 4.94)	No changes
Age ≥ 30 vs. < 30	3	2.59 (1.51 – 4.44)	2	3.32 (1.57 – 7.01)	No changes
Age continuous	26	0.47 (0.38 – 0.59)	21	0.46 (0.36 – 0.59)	No changes
Sex	73	0.88 (0.79 – 0.98)	52	0.87 (0.77 – 0.99)	No changes
Marx score at primary injury	2	1.65 (0.53 – 5.16)	No studies removed	NA	No changes
Tegner score at primary injury (continuous)	8	2.29 (1.48 – 3. 55)	No studies removed	NA	No changes
Tegner score at primary injury ≥7 vs. < 7	3	3.91 (1.69 – 9.01)	No studies removed	NA	No changes
Lateral tibial slope	8	2.21 (1.26 – 3.86)	7	2.27 (1.20 – 4.28)	No changes
Medial tibial slope	8	1.31 (0.76 – 2.24)	6	1.11 (0.47 – 2.61)	No changes
Femoral condyle ratio	2	1.19 (0.65 – 2.19)	No studies removed	NA	No changes
Duration between injury and surgery ≥ 12 vs. < 12 months	2	1.87 (1.58 – 2.22)	No studies removed	NA	No changes
Duration between injury and surgery ≥ 6 vs. < 6 months	2	1.57 (0.58 – 3.74)	NA	NA	Insufficient studies retained to perform meta-analyses
Duration between injury and surgery ≥ 3 vs. < 3 months	2	1.40 (0.29 – 6.79)	No studies removed	NA	No changes
Return to pre-injury activity level	11	1.87 (1.21 – 2.91)	8	1.85 (1.12 – 3.07)	No changes
Family history of ACL injury	11	1.76 (1.34 – 2.31)	7	1.99 (1.50 – 2.65)	No changes

KOOS ADL	2	0.89 (0.39 – 2.02)	No studies removed	NA	No changes
KOOS pain	2	0.95 (0.80 – 1.12)	No studies removed	NA	No changes
KOOS QoL	2	1.01 (0.85 – 0.19)	No studies removed	NA	No changes
KOOS sport/recreation	2	0.96 (0.81 – 1.13)	No studies removed	NA	No changes
KOOS symptom	2	0.81 (0.69 – 0.95)	No studies removed	NA	No changes
Psychological readiness to RTS	2	2.18 (1.32 – 2.61)	NA	NA	Insufficient studies retained to perform meta-analyses
Concomitant cartilage injury	9	0.70 (0.62 – 0.79)	No studies removed	NA	No changes
Concomitant meniscal tear	10	1.07 (0.94 – 1.22)	No studies removed	NA	No changes
Concomitant meniscal repair	4	1.00 (0.60 – 1.66)	No studies removed	NA	No changes
Concomitant Meniscectomy	9	1.16 (0.93 – 1.46)	8	1.16 (0.92 – 1.46)	No changes
Concomitant MCL injury	2	0.99 – (0.70 – 1.41)	No studies removed	NA	No changes
BMI ≥ 25 vs. < 25	3	1.22 (0.48 – 3.11)	2	0.70 (0.56 – 0.89)	BMI ≥ 25 associated with lower odds of graft rupture
BMI (continuous)	12	0.87 (0.73 – 1.03)	11	0.85 (0.69 – 1.06)	No changes
Smoking status	5	0.77 (0.45 – 1.31)	No studies removed	NA	No changes
Contact vs non-contact mechanism of primary injury	4	1.70 (0.75 – 3.87)	2	2.12 (0.80 – 5.59)	No changes
General joint laxity	2	3.20 (0.37 – 27.93)	NA	NA	Insufficient studies retained to perform meta-analyses
Growth-plate status	2	1.04 (0.44 – 2.48)	No studies removed	NA	No changes

Number of physical therapy visits	2	1.19 (0.40 – 3.48)	No studies removed	NA	No changes
Timing of RTS ≥ 6 vs. <6 months	2	1.12 (0.68 – 1.83)	NA	NA	Insufficient studies retained to perform meta-analyses
Type of sport (soccer vs other sports)	5	1.32 (0.82 – 2.15)	No studies removed	NA	No changes
Hop performance (SLHD)	4	0.99 (0.65 – 1.50)	2	1.10 (0.38 – 3.18)	No changes
Hop performance (THD)	2	1.15 (0.74 – 1.81)	NA	NA	Insufficient studies retained to perform meta-analyses
Knee abduction	2	0.53 (0.15 – 1.91)	No studies removed	NA	No changes
Q-ceps peak torque	2	1.06 (0.61 – 1.83)	NA	NA	Insufficient studies retained to perform meta-analyses
Hamstring peak torque	2	0.59 (0.34 – 1.02)	NA	NA	Insufficient studies retained to perform meta-analyses

OR = odds ratio, ACL = anterior cruciate ligament, KOOS = Knee injury and Osteoarthritis Outcome Score, ADL = activities of Daily Living, QoL = Quality of Life, RTS = return to sport, MCL = medial collateral ligament, BMI = body mass index, SLHD = single-leg hop for distance, THD = triple hop for distance, NA = not applicable
